# Supplementary material for: Multiple health behaviour change interventions for primary prevention of cardiovascular disease in primary care: systematic review and meta-analysis
Source: BMJ Open. 2017 Jun 15;7(6):e015375. doi: 10.1136/bmjopen-2016-015375 (PMC5734412; doi:10.1136/bmjopen-2016-015375)
Supplement: Supplementary data [file bmjopen-2016-015375supp005.pdf]

Appendix A

Search strategy

CENTRAL search strategy

ID	Search	Hits
#1	MeSH descriptor CARDIOVASCULAR DISEASES this term only	480
#2	MeSH descriptor CORONARY DISEASE explode all trees	356
#3	cardiovascular in All Text	2052
#4	(coronary in All Text near/3 disease* in All Text)	9
#5	(heart in All Text near/3 disease* in All Text)	11
#6	MeSH descriptor HYPERTENSION this term only	643
#7	hypertension in All Text	1781
#8	(atherosclerosis in All Text or arteriosclerosis in All Text)	258
#9	(hyperlipidaemia in All Text or hyperlipidemia in All Text)	224
#10	MeSH descriptor ARTERIOSCLEROSIS explode all trees	79
#11	MeSH descriptor CHOLESTEROL explode trees all trees	209
#12	MeSH descriptor HYPERLIPIDEMIA explode all trees	33
#13	cholesterol in All Text	630
#14	multiple next risk next factor* in All Text	51
#15	coronary next risk next factor* in All Text	30
#16	(#1 or #2 or #3 or #4 or #5 or #6 or #7 or #8 or #9 or #10)	3105
#17	(#11 or #12 or #13 or #14 or #15)	682
#18	(#16 or #17)	3234
#19	MeSH descriptor HEALTH EDUCATION explode all trees	630
#20	MeSH descriptor HEALTH PROMOTION explode all trees	191
#21	MeSH descriptor HEALTH BEHAVIOR explode all trees	215
#22	MeSH descriptor PRIMARY PREVENTION this term only	1021
#23	MeSH descriptor COUNSELLING this term only	237
#24	counsel* in All Text	1186
#25	(health in All Text near/3 educat* in All Text)	31
#26	(patient in All Text near/3 educat* in All Text)	20
#27	(education* in All Text near/3 program* in All Text)	23
#28	(health in All Text near/3 promotion* in All Text)	2
#29	(health in All Text near/3 behaviour* in All Text)	11
#30	(health in All Text near/3 behavior* in All Text)	9
#31	primary next prevention in All Text	379
#32	(multiple next risk in All Text near/3 intervention* in All Text)	6
#33	(multifactor* in All Text near/3 intervention* in All Text)	9
#34	(multifactor* in All Text near/3 prevention in All Text)	1
#35	(risk next factor* in All Text near/3 reduc* in All Text)	10
#36	(risk next factor* in All Text near/3 manag* in All Text)	20
#37	(risk next factor* in All Text near/3 intervent* in All Text)	49

#38 (lifestyle in All Text near/3 intervention* in All Text) 34
#39 (lifestyle in All Text near/3 advice in All Text) 6
#40 (life-style in All Text near/3 intervention* in All Text) 12
#41 (life-style in All Text near/3 advice in All Text) 2
#42 (life-style in All Text near/3 alter* in All Text) 1
#43 (lifestyle in All Text near/3 alter* in All Text) 5
#44 (lifestyle in All Text near/3 educat* in All Text) 15
#45 (life-style in All Text near/3 educat* in All Text) 5
#46 (life-style in All Text near/3 chang* in All Text) 8
#47 (lifestyle in All Text near/3 chang* in All Text) 18
#48 (behavior* in All Text near/3 chang* in All Text) 24
#49 (behaviour* in All Text near/3 chang* in All Text) 37
#50 (health next care in All Text near/3 advice in All Text) 7
#51 (healthcare in All Text near/3 advice in All Text) 8
#52 nonpharmacologic* in All Text 46
#53 non-pharmacologic* in All Text 562
#54 (#19 or #20 or #21 or #22 or #23 or #24 or #25 or #26 or #27 or #28 or #29) 2311
#55 (#30 or #31 or #32 or #33 or #34 or #35 or #36 or #37 or #38 or #39) 451
#56 (#40 or #41 or #42 or #43 or #44 or #45 or #46 or #47 or #48 or #49 or #50 or #51 or #52 or #53) 646
#57 (#54 or #55 or #56) 2915
#58 (#18 and #57) 1293

Embase search strategy

1. cardiovascular disease/
2. exp ischemic heart disease/
3. (Coronary adj3 disease$).tw.
4. heart disease$.tw.
5. Hypertension/
6. hypertension.tw.
7. (cardiovascular adj3 (disease$ or fit of fitness)).tw.
8. exp arteriosclerosis/
9. exp hyperlipidemia/
10. hyperlipid?emia.tw.
11. cholesterol.tw.
12. arteriosclero$.tw.
13. atheroscleo$.tw.
14. coronary risk factor$.tw.
15. multiple risk factor$.tw.
16. cardiovascular risk factor$.tw.
17. or/1-16
18. exp health education/
19. exp health behavior/

20. primary prevention/
21. exp counseling/
22. (multifactor$ adj5 (intervent$ or prevent$)).tw.
23. ((life-style or life style or lifestyle or healthcare or health care) adj3 (intervention$ or educat$ or advice or alter$ or change$)).tw.
24. primary prevention.tw.
25. (risk factor$ adj3 (reduc$ or manage$ or managing or intervent$ or program$)).tw.
26. (educat$ adj3 (program$ or patient$)).tw.
27. (non pharmacologic$ or nonpharmacologic$).tw.
28. (risk factor$ adj3 modif$).tw.
29. ((lifestyle or life-style or life style) adj3 modif$).tw.
30. exp behavior therapy/
31. (behavi?r$ adj3 (intervention$ or program$ or modif$ or change$ or alter$)).tw.
32. (promot$ adj3 (health or healthcare or health care)).tw.
33. or/18-32
34. 17 and 33
35. random$.ti,ab.
36. factorial$.ti,ab.
37. (crossover$ or cross over$ or cross-over$).ti,ab.
38. placebo$.ti,ab.
39. (double$ adj blind$).ti,ab.
40. (singl$ adj blind$).ti,ab.
41. assign$.ti,ab.
42. allocat$.ti,ab.
43. volunteer$.ti,ab.
44. Crossover Procedure/
45. Double Blind Procedure/
46. Randomized Controlled Trial/
47. Single Blind Procedure/
48. or/35-47
49. exp animal/
50. nonhuman/
51. exp animal experiment/
52. or/49-51
53. exp human/
54. 52 not 53
55. 48 not 54
56. 55 and 34
57. limit 56 to yr="2006 -Current"

Medline search strategy
1. Cardiovascular Diseases/
2. exp coronary disease/
3. Hypertension/

4. exp Arteriosclerosis/
5. exp Hyperlipidemia/
6. (cardiovascular adj3 disease$).tw.
7. (cardiovascular adj3 (fit or fitness)).tw.
8. (Coronary adj3 disease$).tw.
9. heart disease$.tw.
10. hypertension.tw.
11. hyperlipid?emia.tw.
12. cholesterol.tw.
13. atherosclerosis.tw.
14. arteriosclerosis.tw.
15. coronary risk factor$.tw.
16. multiple risk factor$.tw.
17. cardiovascular risk factor$.tw.
18. or/1-17
19. health promotion/
20. exp health education/
21. exp health behavior/
22. exp counseling/
23. Primary Prevention/
24. (multifactor$ adj5 (intervent$ or prevent$)).tw.
25. ((lifestyle or life-style) adj3 (intervention$ or educat$ or advice$ or alter$ or change$)).tw.
26. ((lifestye or life-style or behavior?r$) adj3 (intervention$ or educat$ or advice$ or alter$ or change$)).tw.
27. ((healthcare or health care) adj3 advice).tw.
28. primary prevention.tw.
29. (risk factor$ adj3 (reduc$ or manage$ or managing or intervent$ or program$)).tw.
30. (educat$ adj3 (program$ or patient$)).tw.
31. ((health or healthcare or health care) adj3 (educat$ or advice or promot$)).tw.
32. (nonpharmacologic$ or non-pharmacologic$).tw.
33. ((lifestyle or life style or life-style or behavio?r$ or risk factor$) adj3 modif$).tw.
34. or/19-33
35. 18 and 34
36. randomized controlled trial.pt.
37. controlled clinical trial.pt.
38. Randomized controlled trials/
39. random allocation.sh.
40. double blind method.sh.
41. single-blind method.sh.
42. or/36-41
43. clinical trial.pt.
44. exp Clinical trial/
45. (clin$ adj25 trial$).ti,ab.

46. ((singl$ or doubl$ or trebl$ or tripl$) adj (blind$ or mask$)).ti,ab.
47. placebos.sh.
48. placebo$.ti,ab.
49. random$.ti,ab.
50. research design.sh.
51. or/43-50
52. exp animal/ not humans/
53. 42 or 51
54. 53 not 52
55. 54 and 35

PsycINFO search strategy:

1. cardiovascular disease.mp.
2. hypertension.mp.
3. (Coronary adj3 disease$).mp.
4. heart disease$.mp.
5. (cardiovascular adj3 (disease$ or fit of fitness)).mp. [mp=title, abstract, heading word, table of contents, key concepts, original title, tests & measures]
6. exp Arteriosclerosis/
7. hyperlipid?emia.mp.
8. cholesterol.mp.
9. arterioscler o$.mp.
10. atheroscler o$.mp.
11. coronary risk factor$.mp.
12. multiple risk factor$.mp.
13. cardiovascular risk factor$.mp.
14. or/1-13
15. exp health education/
16. exp health education/
17. exp health promotion/
18. exp preventive medicine/
19. exp counseling/
20. primary prevention.mp.
21. (multifactor$ adj5 (intervent$ or prevent$)).mp.
22. behavior change.mp.
23. exp Obesity/ or exp Food Intake/ or diet intervention.mp. or exp Weight Loss/ or exp Diets/ or exp Overweight/ or exp Weight Control/ or exp Nutrition/
24. exp Nicotine/ or exp Tobacco Smoking/ or exp Smoking Cessation/ or cigarette.mp. or exp Drug Dependency/
25. exp Alcohol Drinking Patterns/ or exp Drinking Behavior/ or exp Alcohol Drinking Attitudes/ or exp Binge Drinking/ or drinking.mp.
26. exp Physical Activity/ or exp Intervention/ or exp Exercise/ or exp Physical Fitness/ or exp Motor Performance/ or physical training.mp.
27. 23 and 24
28. 23 and 25

29. 23 and 26
30. 24 and 25
31. 24 and 26
32. 25 and 26
33. ((life-style or life style or lifestyle or healthcare or health care) adj3 (intervention$ or educat$ or advice or alter$ or change$)).mp.
34. primary prevention.mp.
35. (risk factor$ adj3 (reduc$ or manage$ or managing or intervent$ or program$)).sh.
36. (educat$ adj3 (program$ or patient$)).mp.
37. (non pharmacologic$ or nonpharmacologic$).mp.
38. (risk factor$ adj3 modif$).mp.
39. ((lifestyle or life-style or life style) adj3 modif$).mp.
40. (behavi?r$ adj3 (intervention$ or program$ or modif$ or change$ or alter$)).mp.
41. (promot$ adj3 (health or healthcare or health care)).mp.
42. 15 or 16 or 17 or 18 or 19 or 20 or 21 or 22 or 27 or 28 or 29 or 30 or 31 or 32 or 33 or 34 or 35 or 36 or 37 or 38 or 39 or 40 or 41
43. 14 and 42
44. random$.ti,ab.
45. factorial$.ti,ab.
46. (crossover$ or cross over$ or cross-over$).ti,ab.
47. placebo$.ti,ab.
48. (double$ adj blind$).ti,ab.
49. (singl$ adj blind$).ti,ab.
50. assign$.ti,ab.
51. allocat$.ti,ab.
52. volunteer$.ti,ab.
53. ("double-blind" or "random* assigned" or control).mp.
54. treatment effectiveness evaluation.mp.
55. treatment outcome clinical trial$.mp.
56. (controlled trial$ and clinical trial$).mp. [mp=title, abstract, heading word, table of contents, key concepts, original title, tests & measures]
57. 44 or 45 or 46 or 47 or 48 or 49 or 50 or 51 or 52 or 53 or 54 or 55 or 56
58. 43 and 57
