# Supplementary material for: Multiple health behaviour change interventions for primary prevention of cardiovascular disease in primary care: systematic review and meta-analysis
Source: BMJ Open. 2017 Jun 15;7(6):e015375. doi: 10.1136/bmjopen-2016-015375 (PMC5734412; doi:10.1136/bmjopen-2016-015375)
Supplement: Supplementary data [file bmjopen-2016-015375supp001.pdf]

Supplementary table 1: Trial characteristics of included studies.

Study (Year)	Country	Number of Participants	Selection criteria	Targeted behaviours	Follow-up duration	Intervention reported outcomes
**Kranjčević, et al.** [1]	Croatia	1957	Men and women, aged ≥40.	Diet and PA.	18 months	CVD-risk, weight, BP, cholesterol, smoking, alcohol and PA.
**Vetter, et al.** [2]	United States	390	Men and women, aged≥21 years, BMI= 30-50kg/m^2, elevated waist circumference.	Diet and PA.	2 years	Weight, BP and cholesterol.
**Lakerveld, et al.** [3]	Netherlands	622	Men and women, aged: 30-50 years.	Diet, PA and smoking.	12 months	CVD-risk, smoking, diet and PA.
**Hardcastle, et al.** [4]	United Kingdom	334	Men and women, aged 18-65 years and have at least one CVD risk factor.	Diet and PA.	18 months	Weight, BP, cholesterol, diet and PA.
**Tiessen, et al.** [5]	Netherlands	201	Men aged: 50-75 years old and women aged: 55-75 years and CVD-risk (SCORE) ≥ 5%.	PA, diet and smoking.	12 months	CVD-risk, weight, BP, cholesterol, smoking and PA.
**Parra-Medina, et al.** [6]	United States	266	African-American women, aged≥35 years, baseline BP <160/95.	PA and diet.	12 months	Diet and PA.
**Drevenhorn, et al.** [7]	Sweden	153	Hypertensive patients, men and women aged <75 years, elevated BP, BMI ≥ 25, serum cholesterol ≥ 6.5 and/or serum triglycerides ≥ 2.3 and not reporting regular PA.	Smoking, alcohol, weight, PA and stress	2 years	Weight, BP, cholesterol, alcohol and PA.
**Brett, et al.** [8]	Australia	1200	Men and women aged 40-80 years, without a history of CVD.	Diet, PA and smoking.	12 months	CVD-risk, weight, BP and cholesterol.
**Harris, et al.** [9]	Australia	814	Men and women, aged 40-55 years with recorded diagnosis of hypertension and/or hyperlipidaemia or aged 56-64 years.	Diet, PA, smoking and alcohol.	12 months	CVD-risk, weight, BP, cholesterol, smoking, alcohol, diet and PA.
**Mendis, et al.** [10]	China	1209	Men and women aged 30-70 years with SBP in the range (140-179 mmHg).	Smoking cessation, PA and diet.	12 months	Weight, BP, smoking and diet.
	Nigeria	1188				
**Koelewijn-van Loon, et al.** [11]	Netherlands	615	One or more of the following: BP≥ 140 or on treatment for high BP; total cholesterol ≥ 6.5 or on treatment for high cholesterol; smoker aged ≥ 50 years (men) or ≥ 55 years (women); diabetes; a family history of CVD; and obese.	Smoking status, diet, PA and alcohol use.	12 months	CVD-risk, BP, cholesterol, smoking, diet and PA.
**Eriksson, et al.** [12]	Sweden	151	Men and women aged 18–65 years with hypertension, dyslipidaemia, type 2 diabetes or obesity.	Diet and PA.	3 years	Weight, BP, cholesterol, smoking and PA.
**Phelan, et al.** [13]	United States	224	Men and women aged 18–65 years and BMI of 30–45 kg/m^2.	Diet and PA.	12 months	Weight, BP, cholesterol and diet.

**Harting, et al.** [14]	Netherlands	1300	Men and women who have a greater than 20% risk (Framingham) of incurring a CVD event within 10 years.	Diet, PA and smoking.	18 months	Smoking, diet and PA.
**Korhonen, et al.** [15]	Finland	715	Men and women aged 25–74 years, with systolic BP 140–179 and/or diastolic BP 90–109 and/or on treatment for hypertension.	Diet and alcohol (also PA and smoking).	24 months	Weight, BP, cholesterol, alcohol, diet and PA.
**Baron, et al.** [16]	United Kingdom	368	Men and women aged 25 – 60 years.	Diet mainly, but changes in PA, alcohol and smoking were also mentioned.	12 months	Cholesterol and diet.
**Knutsen and Knutsen** [17]	Norway	1373 men, 1143 wives	Men aged 20 – 54 years and women aged 20-49 years, with no known CHD at baseline.	Diet changes, PA and smoking cessation.	6 years	CVD-risk, weight, BP, cholesterol, smoking and PA.
**Nilsson, et al.** [18]	Sweden	86	Men and women, born during the period 1925 – 1952, treated hypertensives.	Diet, smoking, PA and alcohol.	12 months	Weight, BP, cholesterol, smoking and diet.
**Wood, et al.** [19]	United Kingdom	7460 men, 5012 women	Men aged 40-59 and their families.	Smoking, weight, diet, alcohol, and PA.	12 months	CVD-risk, weight, BP, cholesterol and smoking.
**OXCHECK Study Group** [20]	United Kingdom	5559	Men and women aged 35-64.	Diet, smoking and PA.	3 years	CVD-risk, weight, BP, cholesterol, alcohol, diet, PA and smoking.
**Lindholm, et al.** [21]	Sweden	681	Men and women aged 30-59 years, had a moderate hyperlipidaemia, and at least two CVD risk factors.	Diet, smoking and PA.	18 months	CVD-risk, weight, BP, cholesterol, PA and smoking.
**Meland, et al.** [22]	Norway	127	Men aged 30 to 59 years.	Diet, smoking and PA.	12 months	CVD-risk, BP, cholesterol, PA and smoking.
**Avram, et al.** [23]	Romania	253	Men and women under 80 years, without history of CVD but defined as high risk individuals.	Diet and PA.	18 months	Weight, alcohol, diet and PA.
**Steptoe, et al.** [24]	United Kingdom	883	Men and women aged 18 – 69, total cholesterol of 6.5-9; smoker, BMI of 25-35 and lack of regular PA.	Smoking, diet and PA.	12 months	Weight, BP, cholesterol, diet and PA.
**Sartorelli, et al.** [25]	Brazil	104	Men and women aged 30-65 years, body mass index of 24-35 kg/m^2, and non-diabetic.	Diet and PA.	12 months	Weight, BP, cholesterol, diet and PA.
**Ma, et al.** [26]	United States	419	Men and women aged 35 to 85 years, had moderately to severely elevated levels of major modifiable CVD risk factors.	PA, diet and stress reduction.	15 months	CVD-risk, weight, BP and cholesterol.
**Tibblin and Åberg** [27]	Sweden	400	Men and women aged 30 - 69 years, on hypertensive drugs	Diet, PA and stress management.	12 months	Weight, BP and cholesterol.

**Gomez-Huelgas et al (2015)[28]**	Spain	601	Men and women aged 18-80 years, with metabolic syndrome.	Diet and PA.	3 years	Weight, BP, cholesterol, diet and PA.
**Wennehorst et al. [29]**	Germany	83	Men and women aged 18-80 years who had either prediabetes, type 2 diabetes, or were at risk of developing diabetes and/or cardiovascular diseases.	Diet and PA.	12 months	Weight, BP, cholesterol.
**Salisbury et al. [30]**	United Kingdom	641	Men and women aged between 40 and 74 years, had a high risk of a cardiovascular event in the next 10 years, and had one or more of the following modifiable risk factors (systolic blood pressure ≥140 mm Hg, body mass index ≥30, being a current smoker, or any combination of these).	Smoking status, diet, PA and alcohol use.	12 months	CVD-risk, weight, BP, cholesterol, diet, PA and smoking.
**Duncan et al.[31]**	New Zealand	320	Adults aged 35 to 65 years, a 5-year CVD risk of at least 7%, and/or a BMI of at least 33 kg/m2 for participants younger than 50 years	Diet and PA.	12 months	CVD-risk, weight, BP, cholesterol, diet and PA.

Note: BMI: body mass index, PA: physical activity, BP: blood pressure, CVD: cardiovascular diseas

References:

1. Kranjčević K, Marković BB, Lalić DI, et al. Is a targeted and planned GP intervention effective in cardiovascular disease prevention? A randomized controlled trial. Medical science monitor: international medical journal of experimental and clinical research 2014;**20**:1180.
2. Vetter ML, Wadden TA, Chittams J, et al. Effect of lifestyle intervention on cardiometabolic risk factors: results of the POWER-UP trial. International Journal of Obesity 2013;**37**:S19-S24.
3. Lakerveld J, Bot SD, Chinapaw MJ, et al. Motivational interviewing and problem solving treatment to reduce type 2 diabetes and cardiovascular disease risk in real life: a randomized controlled trial. Int J Behav Nutr Phys Act 2013;**10**(47):10.1186.
4. Hardcastle SJ, Taylor AH, Bailey MP, et al. Effectiveness of a motivational interviewing intervention on weight loss, physical activity and cardiovascular disease risk factors: a randomised controlled trial with a 12-month post-intervention follow-up. Int J Behav Nutr Phys Act 2013;**10**(40):1-16.
5. Tiessen AH, Smit AJ, Broer J, et al. Randomized controlled trial on cardiovascular risk management by practice nurses supported by self-monitoring in primary care. BMC family practice 2012;**13**(1):1.
6. Parra-Medina D, Wilcox S, Salinas J, et al. Results of the Heart Healthy and Ethnically Relevant Lifestyle trial: a cardiovascular risk reduction intervention for African American women attending community health centers. American journal of public health 2011;**101**(10):1914-21.
7. Drevenhorn E, Bengtson A, Nilsson PM, et al. Consultation training of nurses for cardiovascular prevention–a randomized study of 2 years duration. Blood pressure 2012;**21**(5):293-99.
8. Brett T, Arnold-Reed D, Phan C, et al. The Fremantle Primary Prevention Study: a multicentre randomised trial of absolute cardiovascular risk reduction. Br J Gen Pract 2012;**62**(594):e22-e28.
9. Harris MF, Fanaian M, Jayasinghe UW, et al. A cluster randomised controlled trial of vascular risk factor management in general practice. Med J Aust 2012;**197**(7):387-93.
10. Mendis S, Johnston SC, Fan W, et al. Cardiovascular risk management and its impact on hypertension control in primary care in low-resource settings: a cluster-randomized trial. Bulletin of the World Health Organization 2010;**88**(6):412-19.
11. Koelewijn-van Loon MS, van der Weijden T, van Steenkiste B, et al. Involving patients in cardiovascular risk management with nurse-led clinics: a cluster randomized controlled trial. Canadian Medical Association Journal 2009;**181**(12):E267-E74.
12. Eriksson MK, Franks PW, Eliasson M. A 3-year randomized trial of lifestyle intervention for cardiovascular risk reduction in the primary care setting: the Swedish Björknäs study. PloS one 2009;**4**(4):e5195.
13. Phelan S, Wadden T, Berkowitz R, et al. Impact of weight loss on the metabolic syndrome. International journal of obesity 2007;**31**(9):1442-48.
14. Harting J, van Assema P, van Limpt P, et al. Cardiovascular prevention in the Hartslag Limburg project: effects of a high-risk approach on behavioral risk factors in a general practice population. Preventive medicine 2006;**43**(5):372-78.
15. Korhonen M, Kastarinen M, Uusitupa M, et al. The effect of intensified diet counseling on the diet of hypertensive subjects in primary health care: a 2-year open randomized controlled trial of lifestyle intervention against hypertension in eastern Finland. Preventive medicine 2003;**36**(1):8-16.

16. Baron JA, Gleason R, Crowe B, et al. Preliminary trial of the effect of general practice based nutritional advice. Br J Gen Pract 1990;**40**(333):137-41.
17. Knutsen SF, Knutsen R. The Tromsø Survey: the Family Intervention study—the effect of intervention on some coronary risk factors and dietary habits, a 6-year follow-up. Preventive medicine 1991;**20**(2):197-212.
18. Nilsson PM, Lindholm LH, Scherstén BF. Life style changes improve insulin resistance in hyperinsulinaemic subjects: a one-year intervention study of hypertensives and normotensives in Dalby. Journal of hypertension 1992;**10**(9):1071-78.
19. Wood D, Kinmonth A, Davies G, et al. Randomised controlled trial evaluating cardiovascular screening and intervention in general practice: principal results of British family heart study. Bmj 1994;**308**(6924):313-20.
20. OXCHECK. Effectiveness of health checks conducted by nurses in primary care: final results of the OXCHECK study. BMJ: British Medical Journal 1995:1099-104.
21. Lindholm LH, Ekbom T, Dash C, et al. The impact of health care advice given in primary care on cardiovascular risk. BMJ 1995;**310**(6987):1105-09.
22. Meland E, Lærum E, Ulvik RJ. Effectiveness of two preventive interventions for coronary heart disease in primary care. Scandinavian journal of primary health care 1997;**15**(1):57-63.
23. Avram C, Iurciuc M, Craciun L, et al. Dietary and physical activity counseling in high-risk asymptomatic patients with metabolic syndrome–A primary care intervention. Journal of Food, Agriculture & Environment 2011;**9**(3&4):16-19.
24. Steptoe A, Day S, Doherty S, et al. Behavioural counselling in general practice for the promotion of healthy behaviour among adults at increased risk of coronary heart disease: randomised trialCommentary: Treatment allocation by the method of minimisation. Bmj 1999;**319**(7215):943-48.
25. Sartorelli DS, Sciarra EC, Franco LJ, et al. Beneficial effects of short-term nutritional counselling at the primary health-care level among Brazilian adults. Public health nutrition 2005;**8**(07):820-25.
26. Ma J, Berra K, Haskell WL, et al. Case management to reduce risk of cardiovascular disease in a county health care system. Archives of internal medicine 2009;**169**(21):1988-95.
27. Tibblin G, Åberg H. NON-PHARMACOLOGICAL TREATMENT OF HYPERTENSION IN TWO STEPS-1 YEAR REPORT FROM EIGHT HEALTH CENTRES. Acta Medica Scandinavica 1986;**220**(S714):105-12.
28. Gomez-Huelgas R, Jansen-Chaparro S, Baca-Osorio AJ, et al. Effects of a long-term lifestyle intervention program with Mediterranean diet and exercise for the management of patients with metabolic syndrome in a primary care setting. European Journal of Internal Medicine 2015;**26**(5):317-23.
29. Wennehorst K, Mildenstein K, Saliger B, et al. A comprehensive lifestyle intervention to prevent type 2 diabetes and cardiovascular diseases: The german chip trial. Prevention Science 2016:No Pagination Specified.
30. Salisbury C, O'Cathain A, Thomas C, et al. Telehealth for patients at high risk of cardiovascular disease: Pragmatic randomised controlled trial. BMJ (Online) 2016;**353 (no pagination)**(i2647).
31. Duncan S, Goodyear-Smith F, McPhee J, et al. Family-centered brief intervention for reducing obesity and cardiovascular disease risk: A randomized controlled trial. Obesity 2016;**24**(11):2311-18.
