# Supplementary material for: Multiple health behaviour change interventions for primary prevention of cardiovascular disease in primary care: systematic review and meta-analysis
Source: BMJ Open. 2017 Jun 15;7(6):e015375. doi: 10.1136/bmjopen-2016-015375 (PMC5734412; doi:10.1136/bmjopen-2016-015375)
Supplement: Supplementary data [file bmjopen-2016-015375supp002.pdf]

Supplementary table 2: Intervention components and behaviour change techniques employed.

Study (Year)	Study groups	Who delivered it	BCTs [1]	Mode of delivery	No. of sessions	Duration of sessions (in mins)
**Kranjčević, et al.** [1]	Intervention	GPs	1.3, 2.1, 9.1	Face to face and written materials	5	Unclear
	Control		Unclear	Unclear	Unclear	Unclear
**Vetter, et al.** [2]	Intervention 1	PCP and lifestyle coach.	1.1, 1.5, 2.3, 8.7, 9.1	Face to face and written materials	32	Visits: 5-7 mins, counselling: 10-15 mins.
	Intervention 2		1.1, 1.5, 2.3, 8.7, 9.1, 11.1	Face to face and written materials	32	Visits: 5-7 mins, counselling: 10-15 mins.
	Control		1.7	Face to face	8	Visits: 5-7 mins.
**Lakerveld, et al.** [3]	Intervention	Nurse	1.2, 1.6	Face to face and phone sessions.	9	Face to face sessions: 30 mins.
	Control		4.1, 5.1	Written materials.	Unclear	Unclear
**Hardcastle, et al.** [4]	Intervention	PA specialist and dietician	1.1, 1.5, 9.2	Face to face.	5	20-30 mins.
	Control		5.1	Written materials.	Unclear	Unclear
**Tiessen, et al.** [5]	Intervention	Practice nurses.	2.2, 2.3, 2.4, 5.1	Face to face.	7	First session: 20 min, other sessions based on patient preference.
	Control		5.1	Face to face and written materials.	One	Unclear.
**Parra-Medina, et al.** [6]	Intervention	PCP, health educators and nurses	1.1, 1.2.	Face to face and telephone sessions and written materials.	Up to 15	First session: 60 mins. Following sessions: 20 mins.
	Control		1.1	Face to face and written materials.	One	5-10 mins.
**Drevenhorn, et al.** [7]	Intervention	Nurses	1.1, 1.5, 5.3, 9.2, 10.4, 11.2	Face to face	Unclear	Unclear
	Control		Unclear	Unclear	Unclear	Unclear
**Brett, et al.** [8]	Intervention	GPs	1.1, 1.3, 2.7	Face to face	5	Unclear
	Control		1.1, 1.3, 2.7	Face to face	2	Unclear
**Harris, et al.** [9]	Intervention	Health practitioner, dietitian or PT	1.1, 1.2, 2.3, 4.1, 6.1, 9.1	Face to face	6	90 mins/ session.
	Control		Unclear	Unclear	Unclear	Unclear
**Mendis, et al.** [10]	Intervention	Health-care workers	2.6, 4.1	Face to face and written materials	4	Unclear
	Control		Unclear	Unclear	Unclear	Unclear
**Koelewijn-van Loon, et al.** [11]	Intervention	Nurses	1.1, 1.2, 1.4, 1.5, 5.1, 9.2	Face to face and telephone sessions	3	Face to face: 10-20 mins, telephone: 10 mins.
	Control		5.1	Face to face	One	Unclear
**Eriksson, et al.** [12]	Intervention	Dietician, PT and assistants.	1.1, 1.2, 1.3, 1.4, 4.1, 5.1, 8.1, 8.7, 9.1, 9.2	Face to face	56	Unclear.
	Control		9.1	Face to face and written materials.	One	Unclear

**Phelan, et al.** [13]	Intervention 1	PCP	2.3, 11.1.	Face to face and written materials.	8	5-10 mins.
	Intervention 2	Psychologist	1.5, 2.3, 9.1	Group sessions.	29	90 mins
	Intervention 3	Psychologist, PCP	1.5, 2.3, 9.1, 11.1	Face to face, group sessions and written material.	37	Face to face: 5-10 mins, group sessions: 90 mins.
	Intervention 4	PCP	1.5, 2.3, 9.1, 11.1	Face to face and written materials.	8	5-10 mins.
**Harting, et al.** [14]	Intervention	Practice assistant and dietician.	1.1, 1.4, 9.1, 11.1	Face to face, telephone sessions and written materials.	Unclear	Unclear
	Control		Unclear	Unclear	Unclear	Unclear
**Korhonen, et al.** [15]	Intervention	Healthcare centre personnel.	1.1, 1.3, 1.4, 2.3, 2.5,4.1, 9.1	Face to face.	7	Unclear
	Control		Unclear	Unclear	Unclear	Unclear
**Baron, et al.** [16]	Intervention	Nurse	5.1, 9.1	Face to face, group sessions and written material.	Unclear	30 mins.
	Control		Unclear	Unclear	Unclear	Unclear
**Knutsen and Knutsen** [17]	Intervention	Physicians and dieticians	1.1, 4.1, 5.1, 6.1, 9.1	Face to face and telephone sessions.	8	Unclear
	Control		Unclear	Unclear	Unclear	Unclear
**Nilsson, et al.** [18]	Intervention	Nurse, dietician or PT.	1.1, 2.2, 3.1, 4.1, 6.1, 9.1, 12.5	Face to face, group sessions and videotapes.	Unclear	Unclear
	Control		2.2, 5.1	Face to face	One	Unclear
**Wood, et al.** [19]	Intervention	Nurses	1.1, 2.7, 5.1, 6.2, 9.1	Face to face and written materials	Unclear	First session: 90 mins.
	Control		9.1	Face to face	One	45 mins
**OXCHECK Study Group** [20]	Intervention	Nurses	1.3, 2.7, 9.1,	Face to face	Unclear	Initial session: 45-60 mins, following sessions:10-20 mins.
	Control		Unclear	None	None	None
**Lindholm, et al.** [21]	Intervention	Doctors and nurses	2.3, 4.1, 5.1, 6.2, 9.1	Face to face, group sessions and written materials	11	Five group sessions: 90 mins, one group session: all day.
	Control		9.1	Face to face and written materials	5	Unclear
**Meland, et al.** [22]	Intervention	GPs	1.8, 2.3, 8.7, 9.1, 11.2	Face to face and written materials	4	Unclear
	Control		9.1	Face to face and written materials	4	Unclear
**Avram, et al.** [23]	Intervention	GPs	1.1, 9.1	Face to face and telephone sessions	21	Face to face sessions:30 mins.
	Control		Unclear	Written materials	None	None
**Steptoe, et al.** [24]	Intervention	Nurses	1.1, 1.4, 9.1, 11.1	Face to face and telephone sessions	2-3	Face to face sessions:20 mins.
	Control		Unclear	Unclear	Unclear	Unclear
**Sartorelli, et al.** [25]	Intervention	Nutritionist	1.1, 1.4, 9.1	Face to face and group sessions and written materials.	4	Unclear
	Control		Unclear	Group session and written materials	1	Unclear

**Ma, et al. [26]**	Intervention	Nurses and dietitians	1.1, 1.2, 1.7, 9.1, 11.1, 11.2	Face to face	8-10	30-60 mins
	Control		Unclear	Unclear	Unclear	Unclear
**Tibblin and Åberg [27]**	Intervention	Nurses and physicians	2.5, 6.1, 9.1	Face to face, group sessions and videotapes and audiotapes.	15	Unclear
	Control		2.5, 9.1	Face to face	15	Unclear
**Gomez-Huelgas et al. [28]**	Intervention	Nurses and physicians	1.3, 1.4, 2.5, 4.1, 9.1	Face to face, group sessions and written materials.	27	Health assessment: 15 mins, nursing visits: 30 mins.
	Control		2.5, 9.1	Face to face and written materials	24	10 mins.
**Wennehorst et al. [29]**	Intervention	Physician and nutritionist	1.4, 3.1, 4.1, 9.1, 11.2.	Face to face, group sessions and written materials.	16	2.5 hrs/ session.
	Control		Unclear	Unclear	Unclear	Unclear
**Salisbury et al. [30]**	Intervention	Health advisors	1.1, 1.6, 2.4, 5.1, 9.1, 11.1.	Computerised behavioural management programme and telephone sessions.	12	Telephone sessions: an average of 18 mins/session.
	Control		Unclear	Unclear	Unclear	Unclear
**Duncan et al. [31]**	Intervention	Trained health promoter	1.1, 1.4, 1.5, 2.3, 2.6, 3.1, 8.7.	Face to face group sessions and written materials.	5	60mins/ session.
	Control		2.6	Face to face	unclear	Unclear

[1] as coded in Michie, Richardson et al.[32] taxonomy of behaviour change technique

Note: 1.1 Goal setting (behaviour); 1.2 Problem solving; 1.3 Goal setting (outcome); 1.4 Action planning; 1.5 Review behaviour goals(s); 1.6 Discrepancy between current behaviour and goal; 1.7 Review outcome goal(s); 1.8 Behavioural contract; 2.1 Monitoring of behaviour by others without feedback; 2.2 Feedback on behaviour; 2.3 self-monitoring of behaviour; 2.4 Self-monitoring of outcome(s) of behaviour; 2.5 Monitoring of outcomes of behaviour without feedback; 2.6 Biofeedback; 2.7 Feedback on outcome(s) of behaviour; 3.1 Social support (unspecified); 4.1 Instructions on how to perform a behaviour; 5.1 Information about health consequences; 5.3 Information about social and environmental consequences; 6.1 Demonstration of the behaviour; 6.2 Social comparison; 8.1 Behavioural practice/rehearsal; 8.7 Graded tasks; 9.1 Credible source; 9.2 Pros and cons; 10.4 Social reward; 11.1 Pharmacological support; 11.2 Reduce negative emotions; 12.5 Adding objects to the environment; PT Physiotherapist, PA Physical activity

References:

1. Kranjčević K, Marković BB, Lalić DI, et al. Is a targeted and planned GP intervention effective in cardiovascular disease prevention? A randomized controlled trial. Medical science monitor: international medical journal of experimental and clinical research 2014;**20**:1180.
2. Vetter ML, Wadden TA, Chittams J, et al. Effect of lifestyle intervention on cardiometabolic risk factors: results of the POWER-UP trial. International Journal of Obesity 2013;**37**:S19-S24.
3. Lakerveld J, Bot SD, Chinapaw MJ, et al. Motivational interviewing and problem solving treatment to reduce type 2 diabetes and cardiovascular disease risk in real life: a randomized controlled trial. Int J Behav Nutr Phys Act 2013;**10**(47):10.1186.
4. Hardcastle SJ, Taylor AH, Bailey MP, et al. Effectiveness of a motivational interviewing intervention on weight loss, physical activity and cardiovascular disease risk factors: a randomised controlled trial with a 12-month post-intervention follow-up. Int J Behav Nutr Phys Act 2013;**10**(40):1-16.
5. Tiessen AH, Smit AJ, Broer J, et al. Randomized controlled trial on cardiovascular risk management by practice nurses supported by self-monitoring in primary care. BMC family practice 2012;**13**(1):1.
6. Parra-Medina D, Wilcox S, Salinas J, et al. Results of the Heart Healthy and Ethnically Relevant Lifestyle trial: a cardiovascular risk reduction intervention for African American women attending community health centers. American journal of public health 2011;**101**(10):1914-21.
7. Drevenhorn E, Bengtson A, Nilsson PM, et al. Consultation training of nurses for cardiovascular prevention–a randomized study of 2 years duration. Blood pressure 2012;**21**(5):293-99.
8. Brett T, Arnold-Reed D, Phan C, et al. The Fremantle Primary Prevention Study: a multicentre randomised trial of absolute cardiovascular risk reduction. Br J Gen Pract 2012;**62**(594):e22-e28.
9. Harris MF, Fanaian M, Jayasinghe UW, et al. A cluster randomised controlled trial of vascular risk factor management in general practice. Med J Aust 2012;**197**(7):387-93.
10. Mendis S, Johnston SC, Fan W, et al. Cardiovascular risk management and its impact on hypertension control in primary care in low-resource settings: a cluster-randomized trial. Bulletin of the World Health Organization 2010;**88**(6):412-19.
11. Koelewijn-van Loon MS, van der Weijden T, van Steenkiste B, et al. Involving patients in cardiovascular risk management with nurse-led clinics: a cluster randomized controlled trial. Canadian Medical Association Journal 2009;**181**(12):E267-E74.
12. Eriksson MK, Franks PW, Eliasson M. A 3-year randomized trial of lifestyle intervention for cardiovascular risk reduction in the primary care setting: the Swedish Björknäs study. PloS one 2009;**4**(4):e5195.
13. Phelan S, Wadden T, Berkowitz R, et al. Impact of weight loss on the metabolic syndrome. International journal of obesity 2007;**31**(9):1442-48.
14. Harting J, van Assema P, van Limpt P, et al. Cardiovascular prevention in the Hartslag Limburg project: effects of a high-risk approach on behavioral risk factors in a general practice population. Preventive medicine 2006;**43**(5):372-78.
15. Korhonen M, Kastarinen M, Uusitupa M, et al. The effect of intensified diet counseling on the diet of hypertensive subjects in primary health care: a 2-year open randomized controlled trial of lifestyle intervention against hypertension in eastern Finland. Preventive medicine 2003;**36**(1):8-16.
16. Baron JA, Gleason R, Crowe B, et al. Preliminary trial of the effect of general practice based nutritional advice. Br J Gen Pract 1990;**40**(333):137-41.

17. Knutsen SF, Knutsen R. The Tromsø Survey: the Family Intervention study—the effect of intervention on some coronary risk factors and dietary habits, a 6-year follow-up. Preventive medicine 1991;**20**(2):197-212.
18. Nilsson PM, Lindholm LH, Scherstén BF. Life style changes improve insulin resistance in hyperinsulinaemic subjects: a one-year intervention study of hypertensives and normotensives in Dalby. Journal of hypertension 1992;**10**(9):1071-78.
19. Wood D, Kinmonth A, Davies G, et al. Randomised controlled trial evaluating cardiovascular screening and intervention in general practice: principal results of British family heart study. Bmj 1994;**308**(6924):313-20.
20. OXCHECK. Effectiveness of health checks conducted by nurses in primary care: final results of the OXCHECK study. BMJ: British Medical Journal 1995:1099-104.
21. Lindholm LH, Ekbom T, Dash C, et al. The impact of health care advice given in primary care on cardiovascular risk. BMJ 1995;**310**(6987):1105-09.
22. Meland E, Lærum E, Ulvik RJ. Effectiveness of two preventive interventions for coronary heart disease in primary care. Scandinavian journal of primary health care 1997;**15**(1):57-63.
23. Avram C, Iurciuc M, Craciun L, et al. Dietary and physical activity counseling in high-risk asymptomatic patients with metabolic syndrome–A primary care intervention. Journal of Food, Agriculture & Environment 2011;**9**(3&4):16-19.
24. Steptoe A, Day S, Doherty S, et al. Behavioural counselling in general practice for the promotion of healthy behaviour among adults at increased risk of coronary heart disease: randomised trialCommentary: Treatment allocation by the method of minimisation. Bmj 1999;**319**(7215):943-48.
25. Sartorelli DS, Sciarra EC, Franco LJ, et al. Beneficial effects of short-term nutritional counselling at the primary health-care level among Brazilian adults. Public health nutrition 2005;**8**(07):820-25.
26. Ma J, Berra K, Haskell WL, et al. Case management to reduce risk of cardiovascular disease in a county health care system. Archives of internal medicine 2009;**169**(21):1988-95.
27. Tibblin G, Åberg H. NON-PHARMACOLOGICAL TREATMENT OF HYPERTENSION IN TWO STEPS-1 YEAR REPORT FROM EIGHT HEALTH CENTRES. Acta Medica Scandinavica 1986;**220**(S714):105-12.
28. Gomez-Huelgas R, Jansen-Chaparro S, Baca-Osorio AJ, et al. Effects of a long-term lifestyle intervention program with Mediterranean diet and exercise for the management of patients with metabolic syndrome in a primary care setting. European Journal of Internal Medicine 2015;**26**(5):317-23.
29. Wennehorst K, Mildenstein K, Saliger B, et al. A comprehensive lifestyle intervention to prevent type 2 diabetes and cardiovascular diseases: The german chip trial. Prevention Science 2016:No Pagination Specified.
30. Salisbury C, O'Cathain A, Thomas C, et al. Telehealth for patients at high risk of cardiovascular disease: Pragmatic randomised controlled trial. BMJ (Online) 2016;**353 (no pagination)**(i2647).
31. Duncan S, Goodyear-Smith F, McPhee J, et al. Family-centered brief intervention for reducing obesity and cardiovascular disease risk: A randomized controlled trial. Obesity 2016;**24**(11):2311-18.
32. Michie S, Richardson M, Johnston M, et al. The behavior change technique taxonomy (v1) of 93 hierarchically clustered techniques: building an international consensus for the reporting of behavior change interventions. Annals of behavioral medicine 2013;**46**(1):81-95.
