# Supplementary material for: Multiple health behaviour change interventions for primary prevention of cardiovascular disease in primary care: systematic review and meta-analysis
Source: BMJ Open. 2017 Jun 15;7(6):e015375. doi: 10.1136/bmjopen-2016-015375 (PMC5734412; doi:10.1136/bmjopen-2016-015375)
Supplement: Supplementary data [file bmjopen-2016-015375supp003.pdf]

Supplementary table 3: Risk of bias assessment.

Study (Year)	Risk of bias					
	Sequence generation (randomisation methods) [a]	Allocation concealment [b]	Blinding of participants and personnel to study group allocation [c]	Blinding of outcome assessors [d]	Incomplete outcome data [e]	Selective reporting [f]
Kranjčević, K. et al (2014)	Unclear	Unclear	Unclear	Unclear	High	Low
Vetter et al. (2013)	Low	Low	High	Low	High	Low
Lakerveld et al. (2012)	Low	Low	High	High	High	Low
Hardcastle et al. (2013)	Low	Low	Low	Low	High	Low
Tiessen et al. (2012)	Low	Low	High	High	Unclear	Low
Parra-Medina et al. (2011)	Unclear	Unclear	Low	Low	High	High
Drevenhorn et al. (2012)	Unclear	Unclear	Unclear	Unclear	High	Low
Brett et al. (2012)	Low	High	High	High	Low	High
Harris et al. (2012)	Low	Low	High	Low	Low	Low
Mendis et al. (2010)	Unclear	Unclear	High	High	Low	Unclear
Koelewijn-van Loon et al. (2009)	Low	Low	High	High	High	Low
Eriksson et al. (2009)	Low	Low	High	High	Unclear	Low
Phelan et al. (2007)	Unclear	Unclear	Unclear	Unclear	Unclear	Unclear
Harting et al. (2006)	Unclear	Unclear	Unclear	Unclear	Unclear	Unclear
Korhonen et al. (2003)	High	High	High	Unclear	High	Low
Baron et al. (1990)	Unclear	Unclear	Unclear	Unclear	Unclear	Unclear
Knutsen and Knutsen (1991)	Low	Low	Unclear	Low	Low	Low
Nilsson et al. (1992)	Unclear	Unclear	Unclear	Low	Low	Low
Wood et al. (1994)	Unclear	Unclear	Unclear	Unclear	Unclear	Low
OXCHECK Study group (1995)	Unclear	Unclear	High	Low	Unclear	Low
Lindholm et al. (1995)	Unclear	Unclear	High	Unclear	Low	Low
Meland et al. (1997)	Unclear	Unclear	Unclear	Unclear	Unclear	Unclear
Avram et al. (2011)	Unclear	Unclear	Low	Low	Low	Unclear

Steptoe et al. (1999)	Low	Low	High	High	High	Low
Sartorelli et al. (2005)	Low	Low	Low	High	High	Unclear
Ma et al. (2009)	Low	Low	Low	Low	Unclear	Low
Åberg and Tibblin (1989)	Low	Low	Unclear	Unclear	Low	Unclear
Gomez-Huelgas et al. (2015)	Unclear	Unclear	High	High	High	Unclear
Wennehorst et al. (2016)	Low	Low	Unclear	Unclear	High	Unclear
Salisbury et al. (2016)	Low	Low	High	Low	Low	Low
Duncan et al. (2016)	Low	Low	High	Low	High	Low

[a] Assessment of whether or not methods used to generate the allocation sequence should produce comparable groups.
[b] Assessment of whether or not the method used to conceal allocation sequence is sufficient or not.
[c] Assessment of the methods used to blind study participants and personnel from knowing intervention allocation.
[d] Assessment of the methods used to blind study outcome assessors from knowing intervention allocation, and whether or not this method of blinding is sufficient.
[e] Assessment of whether incomplete outcome data were adequately dealt with. Studies missing outcome data for >20% of participants who underwent randomization were considered at high risk of bias, while studies missing <10% of participants who underwent randomization were considered at low risk of bias.
[f] Assessment of whether all outcome measures described in the introduction and methods section of the paper (and published protocols) were reported.
