# Supplementary material for: Multiple health behaviour change interventions for primary prevention of cardiovascular disease in primary care: systematic review and meta-analysis
Source: BMJ Open. 2017 Jun 15;7(6):e015375. doi: 10.1136/bmjopen-2016-015375 (PMC5734412; doi:10.1136/bmjopen-2016-015375)
Supplement: Supplementary data [file bmjopen-2016-015375supp006.pdf]

Appendix B

Forest plots of pooled effect of multiple behaviour interventions on intervention outcomes.

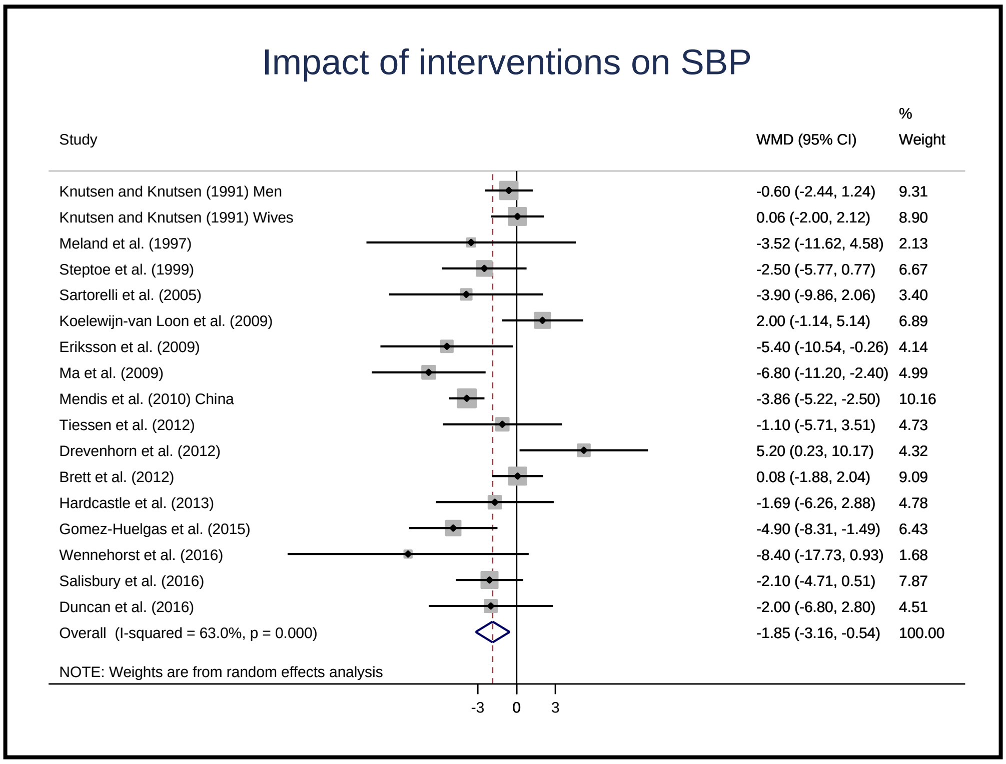

Pooled effect of multiple behaviour interventions on systolic blood pressure (mmHg). Random effects model used. MWD= mean weighted difference. 95% CI = 95% confidence intervals.

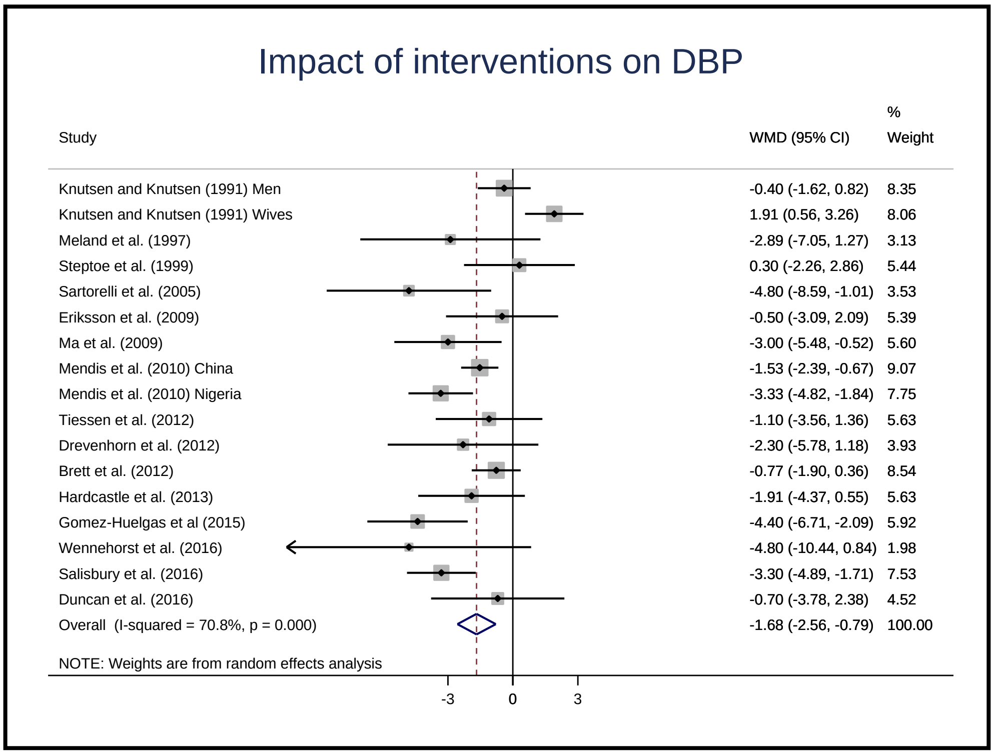

Pooled effect of multiple behaviour interventions on diastolic blood pressure (mmHg). Random effects model used. MWD= mean weighted difference. 95% CI = 95% confidence intervals.

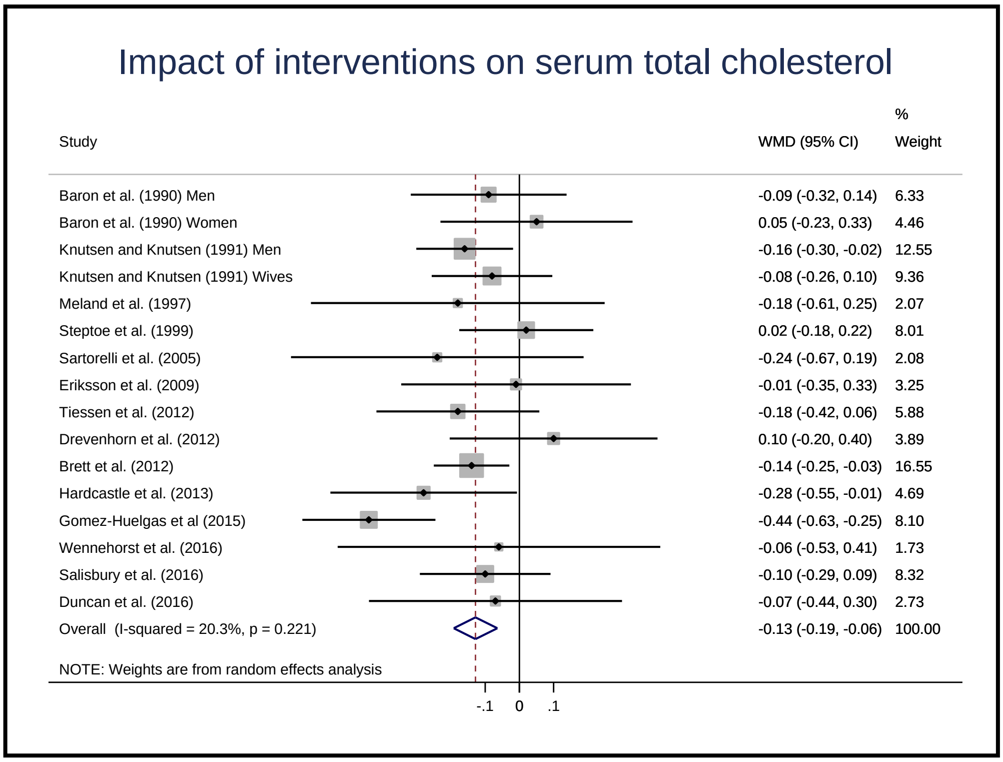

Pooled effect of multiple behaviour interventions on serum total cholesterol (mmol/L). Random effects model used. MWD= mean weighted difference. 95% CI = 95% confidence intervals.

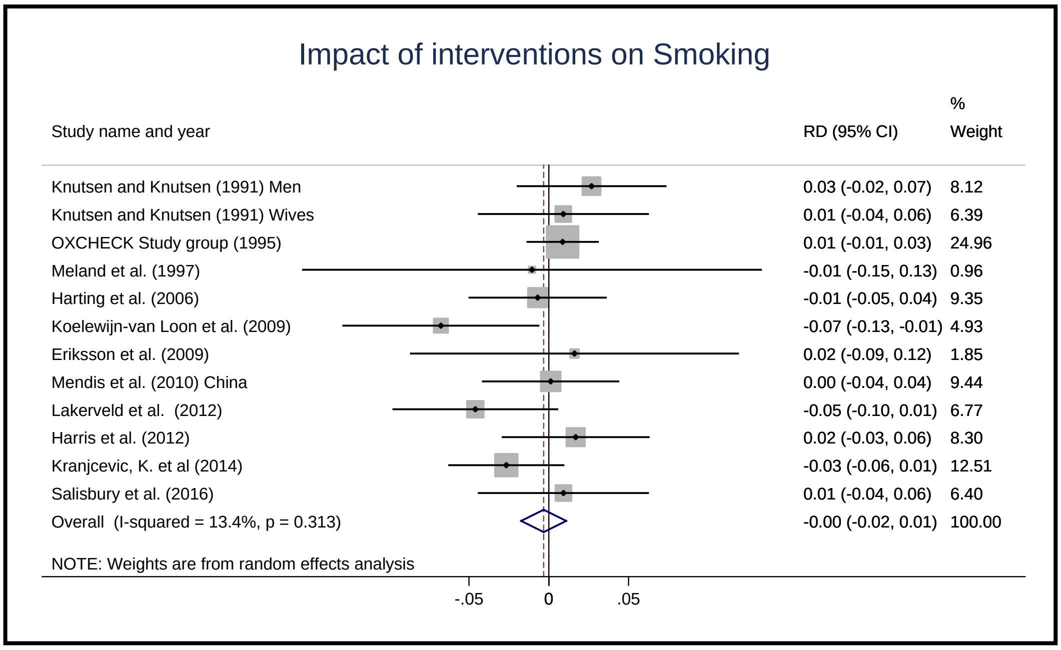

Pooled effect of multiple behaviour interventions on smoking prevalence. Random effects models used. RD= risk difference. 95% CI = 95% confidence intervals.

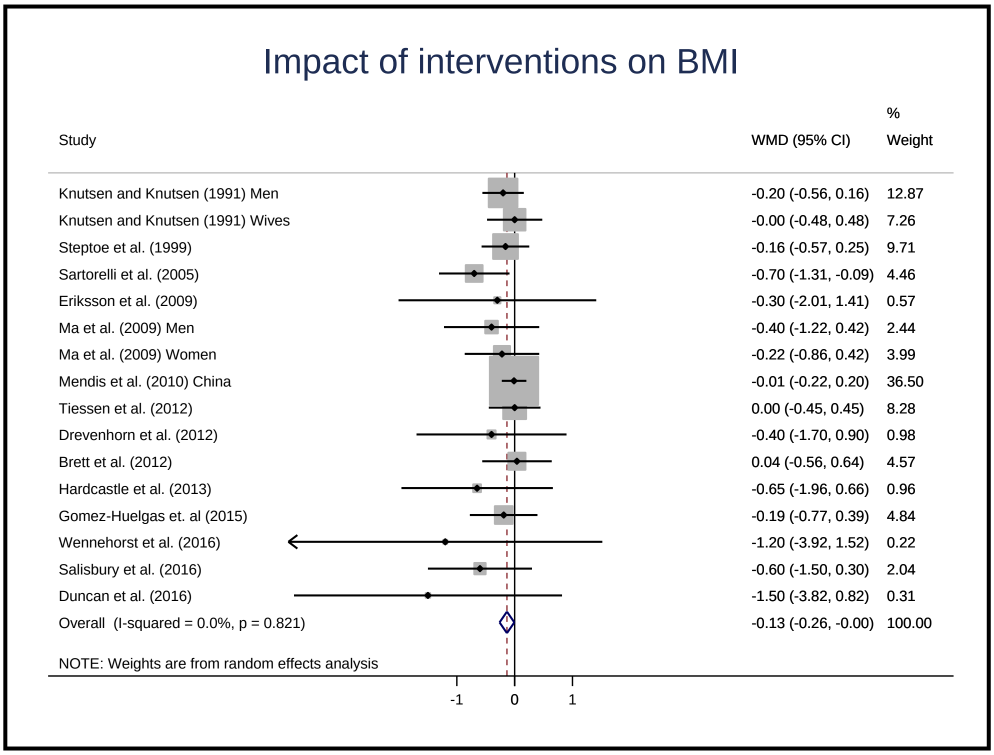

Pooled effect of multiple behaviour interventions on body mass index (Kg/m^2). Random effects model used. MWD= mean weighted difference. 95% CI = 95% confidence intervals.

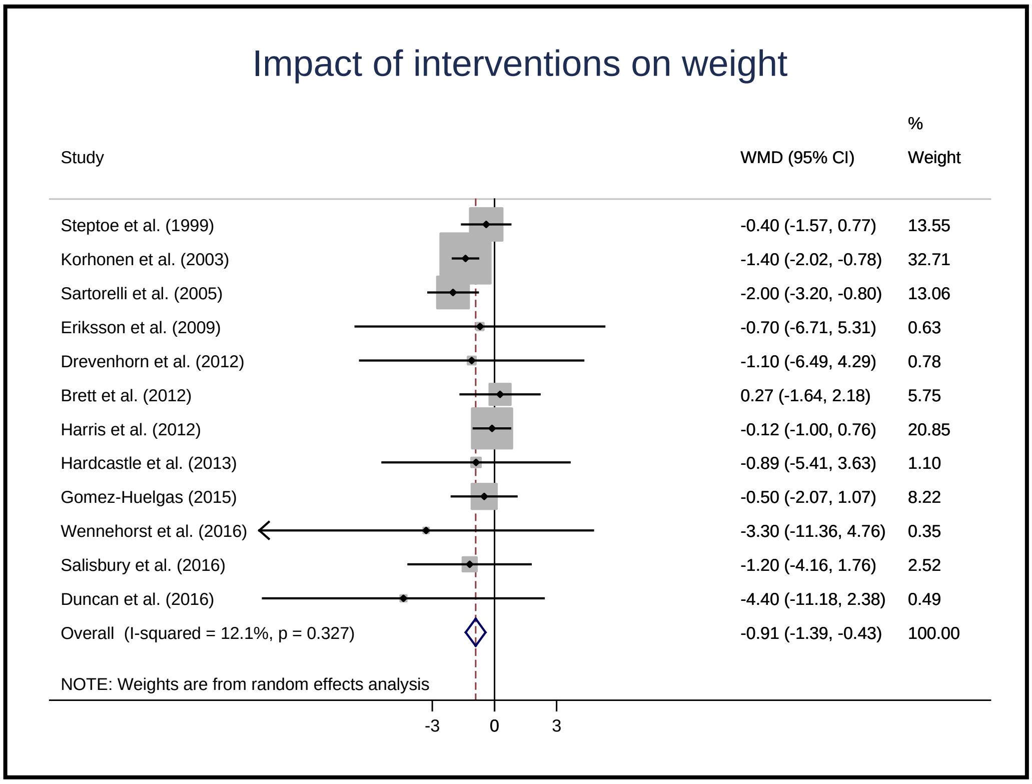

Pooled effect of multiple behaviour interventions on weight (Kg). Random effects model used. MWD= mean weighted difference. 95% CI = 95% confidence intervals.

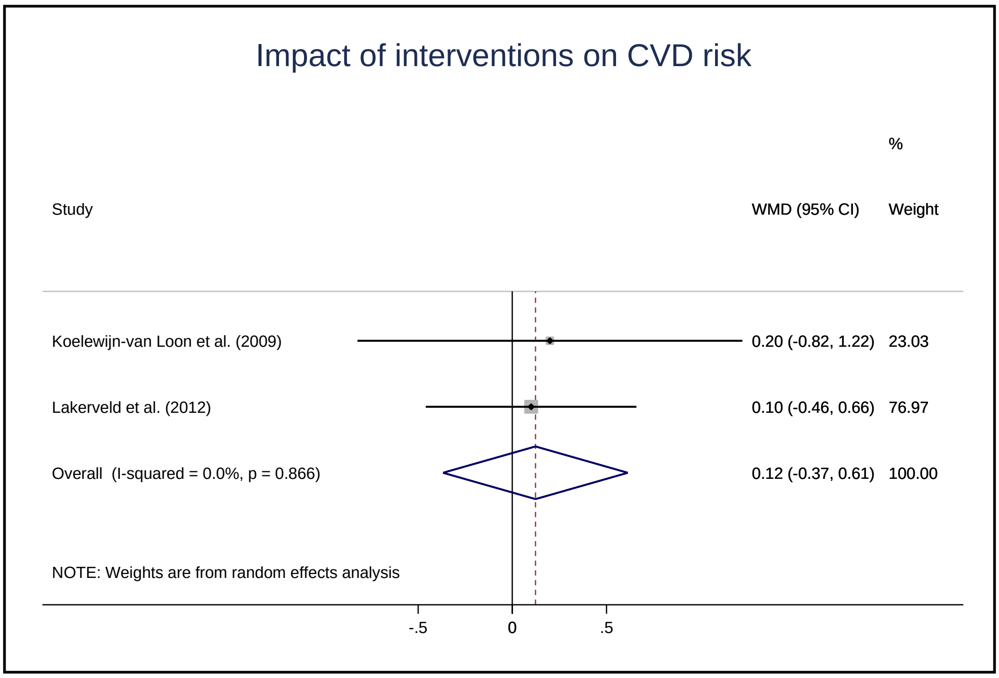

Pooled effect of multiple behaviour interventions on cardiovascular risk (SCORE). Random effect models used. MWD= mean weighted difference. 95% CI = 95% confidence intervals.
