# Supplementary material for: Multiple health behaviour change interventions for primary prevention of cardiovascular disease in primary care: systematic review and meta-analysis
Source: BMJ Open. 2017 Jun 15;7(6):e015375. doi: 10.1136/bmjopen-2016-015375 (PMC5734412; doi:10.1136/bmjopen-2016-015375)
Supplement: Supplementary data [file bmjopen-2016-015375supp004.pdf]

Supplementary table 4: Theory use evaluation using Theory Coding Scheme.

Study	Theory used	Item 1	Item 2	Item 3	Item 4	Item 5	Item 6	Item 7	Item 8	Item 9	Item 10	Item 11	Item 12	Item 13	Item 14	Item 15	Item 16	Item 17	Item 18	Item 19
Kranjčević, K. et al (2014)	Theory use is not reported	N/A	N/A	N/A	N/A	N/A	N/A	N/A	N/A	N/A	N/A	N/A	N/A	N/A	N/A	N/A	N/A	N/A	N/A	N/A
Vetter et al. (2013)	Social cognitive and behavioural self-management theory	Yes	Yes	No	No	Yes	No	No	Yes	Yes	No	Yes	No	N/A	A, B,C and D	No	No	No	No	No
Lakerveld et al. (2012)	Theory of planned behaviour and theory of self-regulation.	Yes	Yes	No	No	Yes	No	Yes	No	Yes	No	Yes	B	A	A and B	Yes	No	Yes	No	No
Hardcastle et al. (2013)	Theory use is not reported	N/A	N/A	N/A	N/A	N/A	N/A	N/A	N/A	N/A	N/A	N/A	N/A	N/A	N/A	N/A	N/A	N/A	N/A	N/A
Tiessen et al. (2012)	Stages of change	Yes	No	Yes	Yes	Do not know	No	No	No	No	No	No	No	N/A	A, B,C and D	Do not now	No	No	Do not know	No
Parra-Medina et al. (2011)	Trans-theoretical model and social cognitive theory	Yes	Yes	No	No	Yes	Yes	Yes	No	Yes	No	Yes	No	No	A	Do not know	No	No	Do not know	No
Drevenhorn et al. (2012)	Stages of changes model	Yes	Yes	Yes	No	Yes	Yes	Yes	No	Yes	Yes	No	No	N/A	A	Do not know	No	No	Do not know	No
Brett et al. (2012)	Theory use is not reported	N/A	N/A	N/A	N/A	N/A	N/A	N/A	N/A	N/A	N/A	N/A	N/A	N/A	N/A	N/A	N/A	N/A	N/A	N/A

Harris et al. (2012)	Stages of changes model	Yes	Yes	Yes	No	No	Yes	No	No	No	No	No	No	N/A	A and B	No	No	No	No	No
Mendis et al. (2010)	Theory use is not reported	N/A	N/A	N/A	N/A	N/A	N/A	N/A	N/A	N/A	N/A	N/A	N/A	N/A	N/A	N/A	N/A	N/A	N/A	N/A
Koelewijn-van Loon et al. (2009)	Theory use is not reported	N/A	N/A	N/A	N/A	N/A	N/A	N/A	N/A	N/A	N/A	N/A	N/A	N/A	N/A	N/A	N/A	N/A	N/A	N/A
Eriksson et al. (2009)	Stages of change model	Yes	Yes	Yes	No	No	No	No	No	No	No	No	No	N/A	No	No	No	No	No	No
Phelan et al. (2007)	Theory use is not reported	N/A	N/A	N/A	N/A	N/A	N/A	N/A	N/A	N/A	N/A	N/A	N/A	N/A	N/A	N/A	N/A	N/A	N/A	N/A
Harting et al. (2006)	Theory use is not reported	N/A	N/A	N/A	N/A	N/A	N/A	N/A	N/A	N/A	N/A	N/A	N/A	N/A	N/A	N/A	N/A	N/A	N/A	N/A
Korhonen et al. (2003)	Theory use is not reported	N/A	N/A	N/A	N/A	N/A	N/A	N/A	N/A	N/A	N/A	N/A	N/A	N/A	N/A	N/A	N/A	N/A	N/A	N/A
Baron et al. (1990)	Theory use is not reported	N/A	N/A	N/A	N/A	N/A	N/A	N/A	N/A	N/A	N/A	N/A	N/A	N/A	N/A	N/A	N/A	N/A	N/A	N/A
Knutsen and Knutsen (1991)	Theory use is not reported	N/A	N/A	N/A	N/A	N/A	N/A	N/A	N/A	N/A	N/A	N/A	N/A	N/A	N/A	N/A	N/A	N/A	N/A	N/A
Nilsson et al. (1992)	Theory use is not reported	N/A	N/A	N/A	N/A	N/A	N/A	N/A	N/A	N/A	N/A	N/A	N/A	N/A	N/A	N/A	N/A	N/A	N/A	N/A
Wood et al. (1994)	Theory use is not reported	N/A	N/A	N/A	N/A	N/A	N/A	N/A	N/A	N/A	N/A	N/A	N/A	N/A	N/A	N/A	N/A	N/A	N/A	N/A
OXCHECK Study group (1995)	Theory use is not reported	N/A	N/A	N/A	N/A	N/A	N/A	N/A	N/A	N/A	N/A	N/A	N/A	N/A	N/A	N/A	N/A	N/A	N/A	N/A

Lindholm et al. (1995)	Theory use is not reported	N/A	N/A	N/A	N/A	N/A	N/A	N/A	N/A	N/A	N/A	N/A	N/A	N/A	N/A	N/A	N/A	N/A	N/A	N/A
Meland et al. (1997)	Theory use is not reported	N/A	N/A	N/A	N/A	N/A	N/A	N/A	N/A	N/A	N/A	N/A	N/A	N/A	N/A	N/A	N/A	N/A	N/A	N/A
Avram et al. (2011)	Theory use is not reported	N/A	N/A	N/A	N/A	N/A	N/A	N/A	N/A	N/A	N/A	N/A	N/A	N/A	N/A	N/A	N/A	N/A	N/A	N/A
Steptoe et al. (1999)	Stages of change model	Yes	Yes	Yes	No	No	No	No	No	No	No	No	Yes	C and F	N/A	N/A	N/A	N/A	N/A	N/A
Sartorelli et al. (2005)	Theory use is not reported	N/A	N/A	N/A	N/A	N/A	N/A	N/A	N/A	N/A	N/A	N/A	N/A	N/A	N/A	N/A	N/A	N/A	N/A	N/A
Ma et al. (2009)	Social cognitive theory and trans-theoretical model	Yes	No	No	No	No	No	No	No	No	No	No	No	N/A	N/A	N/A	N/A	No	No	No
Åberg and Tibblin (1989)	Theory use is not reported	N/A	N/A	N/A	N/A	N/A	N/A	N/A	N/A	N/A	N/A	N/A	N/A	N/A	N/A	N/A	N/A	N/A	N/A	N/A
Gomez-Huelgas et al. (2015)	Theory use is not reported	N/A	N/A	N/A	N/A	N/A	N/A	N/A	N/A	N/A	N/A	N/A	N/A	N/A	N/A	N/A	N/A	N/A	N/A	N/A
Wennehorst et al. (2016)	Theory use is not reported	N/A	N/A	N/A	N/A	N/A	N/A	N/A	N/A	N/A	N/A	N/A	N/A	N/A	N/A	N/A	N/A	N/A	N/A	N/A
Salisbury et al. (2016)	Theory use is not reported	N/A	N/A	N/A	N/A	N/A	N/A	N/A	N/A	N/A	N/A	N/A	N/A	N/A	N/A	N/A	N/A	N/A	N/A	N/A
Duncan et al. (2016)	Theory use is not reported	N/A	N/A	N/A	N/A	N/A	N/A	N/A	N/A	N/A	N/A	N/A	N/A	N/A	N/A	N/A	N/A	N/A	N/A	N/A

Item 1) Theory/ model of behaviour mentioned

Item 2) Targeted construct mentioned

Item 3) Intervention based on single theory

Item 4) Theory used to select recipients

Item 5) Theory used to select intervention techniques

Item 6) Theory used to tailor intervention techniques to recipients

Item 7) All intervention techniques are explicitly linked to theory construct

Item 8) At least one of the intervention techniques are explicitly linked to theory construct

Item 9) Group of techniques are linked to a group of constructs

Item 10) All theory relevant constructs are explicitly linked to at least one intervention technique.

Item 11) At least one of the theory relevant constructs are explicitly linked to at least one intervention technique.

Item 12) theory-relevant constructs are measured

Item 13) Quality of measures

Item 14) Randomization of participants' condition

Item 15) Changes in measured theory-relevant constructs

Item 16) Mediational analysis of constructs

Item 17) Results discussed in relation to theory

Item 18) Appropriate support for theory

Item 19) Results used to refine theory
