# Supplementary material for: Multiple health behaviour change interventions for primary prevention of cardiovascular disease in primary care: systematic review and meta-analysis
Source: BMJ Open. 2017 Jun 15;7(6):e015375. doi: 10.1136/bmjopen-2016-015375 (PMC5734412; doi:10.1136/bmjopen-2016-015375)
Supplement: Author's manuscript [file bmjopen-2016-015375.draft_revisions.pdf]

BMJ Open

Multiple health behaviour change interventions for primary prevention of cardiovascular disease in primary care: systematic review and meta-analysis

Journal:	*BMJ Open*
Manuscript ID	bmjopen-2016-015375
Article Type:	Research
Date Submitted by the Author:	05-Dec-2016
Complete List of Authors:	Alageel, Samah; King's College London, Primary Care and Public Health<br>Wright, Alison; King's College London, Primary care and public health<br>McDermott, Lisa; King's College London, Primary Care and Public Health Sciences<br>Gulliford, Martin; King's College London, UK
**Primary Subject Heading**:	Public health
Secondary Subject Heading:	Cardiovascular medicine
Keywords:	cardiovascular diseases, health behaviour, PRIMARY CARE, primary prevention, meta-analysis

SCHOLARONE™
Manuscripts

Multiple health behaviour change interventions for primary prevention of cardiovascular disease in primary care: systematic review and meta-analysis

Samah Alageel MPH, Alison J. Wright PhD, Lisa McDermott PhD, Martin C Gulliford FFPH

Department of Primary Care and Public Health Sciences, King's College London

Correspondence: **Samah Alageel**
Department of Primary Care and Public Health Sciences,
Faculty of Life Sciences and Medicine.
King's College London, Addison House, Guy's Campus,
London SE1 1UL, United Kingdom
Email: samah.alageel@kcl.ac.uk
Tel: 0207 848 6631
Fax: 0207 848 6620

Word count:

Abstract	232
Text	4113
Tables	4
Figures	1

For peer review only

ABSTRACT

Objectives: It is uncertain whether multiple health behaviour change interventions (MHBC) are effective at reducing cardiovascular disease (CVD) risk in primary care. A systematic review and meta-analysis were performed to evaluate the effectiveness of MHBC interventions on CVD-risk; the study also evaluated associations of theoretical frameworks and intervention components with intervention effectiveness.

Methods: The search included randomised controlled trials of MHBC interventions aimed at reducing CVD-risk in primary care up to 2015. Theoretical frameworks and intervention components were evaluated using standardised methods. Meta-analysis with stratification and meta-regression were used to evaluate intervention effects.

Results: We identified 27 trials (34,839 participants) with a minimum duration of 12 months follow-up. Pooled net change in systolic blood pressure (12 trials) was -1.45 (95% confidence interval -2.98 to 0.09, P= 0.06) mm Hg, diastolic blood pressure (11 trials) -1.01 (-1.91 to -0.11, P= 0.03) mm Hg, body mass index (10 trials) -0.11 (-0.25 to 0.02, P= 0.10) Kg/m^2 and serum total cholesterol (10 trials) -0.11 (-0.17 to -0.05, P <0.001) mmol/L. There was no significant association between interventions with a reported theoretical basis and intervention outcomes, except for body weight (β 1.14, 0.06 to 2.22, P= 0.04). No association was observed between intervention intensity (number of sessions and intervention duration) and intervention outcomes.

Conclusions: MHBC interventions delivered to participants in primary care did not appear to have quantitatively important effects on CVD-risk and CVD risk factors.

Key words: Cardiovascular Diseases, Health Behaviour, Primary Health Care, Meta-analysis, Primary Prevention, Risk Factors.

For peer review only

Strengths and limitations:

- The review includes all 27 published randomised controlled trials of MHBC interventions and cardiovascular risk with follow-up for 12 months or longer
- The study employed standardised instruments to evaluate the impact of theory use and behaviour change techniques in MHBC interventions.
- The majority of trials included were conducted in Europe and United States and only English language publications were included
- Not all studies evaluated all outcomes of interest and some lacked detail concerning intervention design and delivery

For peer review only

INTRODUCTION

Cardiovascular disease (CVD) is the leading cause of death worldwide, accounting for over 30% of global mortality [1]. CVD is mediated by several antecedent behavioural risk factors, and its onset might be prevented or delayed by altering one or several risk factors [1]. Risk factors for CVD are inter-related and often coexist [2-4]. This observation has informed the development of multiple health behaviour change (MHBC) interventions for reduction of CVD-risk. Identifying individuals at high-risk of CVD in primary care, and encouraging lifestyle change to reduce risk factors, represents a widely used strategy for CVD prevention. Randomised controlled trials have been conducted in primary care to evaluate the effectiveness of MHBC interventions using lifestyle modification techniques instead of, or in addition to, pharmacological treatment to modify CVD risk factors. These trials have generally provided only equivocal evidence for reduction of CVD incidence through MHBC but the degree of effectiveness might be associated with level of risk [5-7]. Results from Ebrahim et al.'s [5] systematic review suggested that MHBC interventions have negligible effect on mortality in unselected populations, with a pooled odds ratio for coronary heart disease mortality of 0.99 (95% CI 0.92 to 1.07). Evidence of benefit was found in studies in high-risk populations including people with hypertension (OR 0.78, 0.68 to 0.89) or diabetes (OR 0.71, 0.61 to 0.83) [5]. However, general health checks were not found to reduce all cause-mortality, nor CVD- or cancer-related morbidity and mortality [8].

Previous reviews have assessed the effectiveness of MHBC interventions in reducing CVD morbidity and mortality [5 6 8], less is known about the effectiveness of these interventions in reducing CVD-risk and risk factor values in primary care.

For peer review only

In recent years, there has been growing appreciation of the role of employing psychological theory in behaviour change intervention design, and studying the impact of specific behaviour change techniques (BCT) on intervention outcomes [9]. Theories of the psychological determinants of behaviour can inform the development and evaluation of behaviour change interventions [10]. Interventions that systematically target psychological constructs, that evidence shows are more predictive of behaviour, are likely to be more effective [11]. A review of internet-based interventions suggested that more intensive use of theory was associated with greater behaviour change [12], but another review found little evidence of an association between theory use and intervention effects on healthy eating or physical activity [13]. This equivocal evidence could arise if a high proportion of behaviour change interventions are not based on a theory or the theory is not applied extensively [14].

Behaviour change techniques (BCT) are 'the active components of an intervention designed to change behaviour' [15]. Identifying specific BCTs associated with greater impact on intervention effectiveness is essential for future intervention design [16]. Previous reviews suggested that interventions using the BCTs "provision of instructions," "self-monitoring of behaviour," "relapse prevention," and "prompt practice" led to greater reductions in weight among obese individuals [17], while interventions designed to modify physical activity and/or diet were more effective when they included self-monitoring and particularly when they combined self-monitoring with another BCT associated with control theory [18]. Identifying BCTs associated with greater intervention effectiveness and exploring the impact of applying theory will contribute to the design of future MHBC interventions targeting CVD risk in primary care.

For peer review only

Objectives

This systematic review had three objectives: first, to assess the effectiveness of MHBC interventions, directed at changing two or more behaviours, at reducing CVD-risk and CVD risk factors in adults without existing cardiovascular conditions; secondly, to evaluate whether using theory to develop interventions is associated with intervention effectiveness; and thirdly, to evaluate the association between behaviour change techniques employed and intervention effects.

For peer review only

METHODS

Studies were selected according to the following criteria:

Participants

Trials that recruited an adult population free of CVD were included. Following previous reviews [5], we included trials with less than 20% participants with CVD. Studies of patient populations with established disease, such as diabetes, were excluded.

Interventions

We included studies that evaluated behaviour change interventions aimed at reducing CVD-risk by intervening on two or more risk behaviours at the same time. Risk behaviours included: physical activity, diet, alcohol consumption, use of stress management and smoking. Comparators were usual care or less intensive interventions.

Settings

Interventions where participants were recruited, and interventions were delivered by trained healthcare professionals or primary care staff, in primary care premises (including general practice, family practice or primary care clinic).

Study design

Controlled trials, with individual or cluster randomisation, providing ≥12 month follow-up for outcome evaluation.

For peer review only

Outcome measures

Long term outcomes of MHBC interventions including CVD mortality and clinical events have been reported previously [5 6] and were not included in this systematic review. Primary outcomes were changes in CVD-risk scores, body mass index (BMI) or body weight, blood pressure, and serum total cholesterol levels. Secondary outcomes were changes in physical activity, diet, smoking and alcohol consumption.

Language

Studies reported in English.

Search strategy

Multiple sources of ascertainment were used, including electronic databases (Medline, EMBASE, PsycINFO and CENTRAL) and searching reference lists of included papers. The search results and search terms of the previous review [5] were used with searching extended from 2006 until May 2015. Search strategies are displayed in appendix A. Titles were screened by one reviewer (SA) and a second reviewer (MG) checked a random set of studies, approximately 10% of the search results. The selection process is displayed in Figure 1.

Methodologic quality

Studies were evaluated using the Cochrane risk of bias tool [19]. This assesses six domains of bias including selection bias, performance bias, detection bias, attrition bias, reporting bias and other biases [19].

For peer review only

Data extraction

Interventions were coded by country, target behaviours, participant and intervention characteristics, mode of delivery and intervention outcomes. In addition, Michie and Prestwich's [20] method of assessing the application of theory in the development and evaluation of behaviour change interventions was used. The Theory Coding Scheme (TCS) consists of 19 items that cover different aspects that may be informed by theory [20]. We used three measures to reflect the extent of theory use as reported in previous reviews [12 13]: whether the theory was used to develop intervention's BCTs (item 5 of TCS); the degree to which BCTs were linked to a theory-relevant construct (items 7-9); and the extent to which theory-relevant constructs were explicitly targeted by BCTs (items 9-11).

The theory-based taxonomy of 93 behaviour change techniques developed by Michie, Richardson et al.[9] was used to identify intervention techniques. The assessment was completed by two researchers (LM and SA) with good agreement for intervention groups (77.8% agreement) and control groups (92.6% agreement). Discrepancies were discussed and resolved to reach full agreement. Intervention characteristics and BCTs were also extracted from descriptions of the control group, because the chosen nature of the control group can influence the apparent effectiveness of interventions [21]. We attempted to contact study authors to provide additional information where necessary. However, when information was not available, we assumed missing outcome data to occur at random.

Data analysis

Outcome data were combined in random effects meta-analyses using 'metan' commands in STATA. We quantified statistical heterogeneity using I^2 statistic. Random effects models were chosen due to the considerable heterogeneity for certain outcomes. For continuous outcomes we used mean changes in each trial arm to calculate net effects. We expressed

For peer review only

effects for binary variables as risk differences. Meta-regression were used to examine the effect of number of interventions' sessions, intervention duration, types of BCTs used on intervention outcomes. Intervention duration was calculated by multiplying the number of sessions and the sessions' duration. Publication bias was assessed using Egger's regression test [22] using 'metabias' and 'metafunnel' commands in STATA. Mendis et al [23] Nigeria site's study had unusually high summary estimates, and heterogeneity diminished substantially after this study was excluded. This study was therefore treated as an outlier and results were reported with the exclusion of this study.

For peer review only

RESULTS

The initial search identified 21,290 references, with 55 relevant trials identified from the previous systematic review [5]. After removing duplicates, 16,793 titles were screened. A total of 27 trials were included in this review (Figure 1).

For peer review only

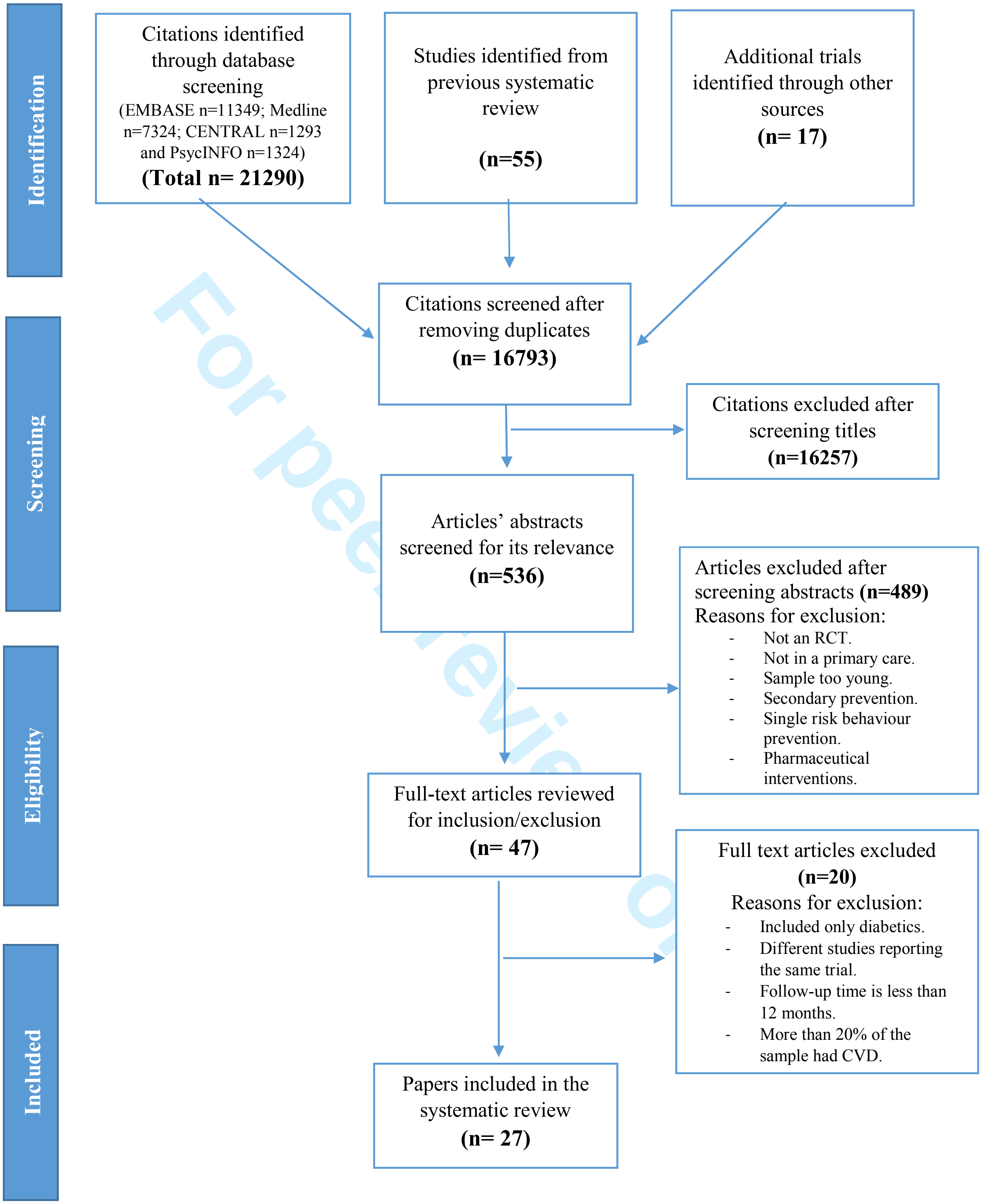

Figure 1: PRISMA flow diagram outlining the systematic review process.

For peer review only

Included studies

We identified a total of 27 trials of MHBC intervention in primary care with 34839 participants. The duration of follow-up ranged from 12 months to 6 years (median 12 months). Intervention duration ranged from three months up to three years (median 12 months). Summary of included studies characteristics are presented in table 1 and supplementary table 1.

For peer review only

Table 1: Summary of characteristics of 27 trials included in the review. Figures are frequencies (column percent).

Characteristics		Freq. (%)
**Total**		27 (100)
**Country**	UK	5 (18.5)
	USA	4 (14.8)
	Europe	14 (51.8)
	Others	4 (14.8)
**Number of participants**	Median (IQR)	419 (224-1200)
**Gender**	Male only	1 (3.7)
	Female only	1 (3.7)
	Both	25 (92.6)
**Age**	Minimum age, median (IQR)	30 (21-40)
	Maximum age, median (IQR)	65 (59-70)
**Intervention outcomes**	CVD risk	12 (44.4)
	Body weight	21 (77.7)
	Blood pressure	22 (81.5)
	Serum cholesterol	22 (81.5)
	Diet	15 (55.5)
	Physical activity	18 (66.7)
	Alcohol	6 (22.2)
	Smoking	14 (51.8)
**Number of targeted behaviours**	2 behaviours	8 (29.6)
	3 behaviours	12 (44.4)
	4 behaviours	6 (22.2)
	5 behaviours	1 (3.7)
**Follow-up duration**	12 months	15 (55.5)
	>12 months	12 (44.4)

CVD, cardiovascular disease; IQR, interquartile range

For peer review only

Study characteristics

Diet and physical activity were targeted in eight trials, with nine trials targeting diet, physical activity and smoking. Diet, physical activity, smoking and alcohol consumption were targeted in six interventions and two interventions targeted diet, physical activity and stress management. Only one intervention targeted diet, physical activity, stress and alcohol consumption and one intervention targeted all five behaviours. A wide range of intervention modalities was investigated (Table 2 and supplementary table 2), including individual and group sessions, telephone conversations and provision of written materials. The majority of the included trials reported offering "usual care" to the control group, with few details provided. Six trials offered face-to-face sessions and six trials offered face-to-face sessions and written materials. Written materials alone were offered in three trials and no intervention was offered to the control group in three interventions.

For peer review only

Table 2: Summary of interventions characteristics for 27 trials included in the review. Figures are frequencies (column percents).

		Intervention N (%)	Control N (%)
**Type of staff delivering intervention**	GPs and physicians	7 (25.9)	
	Nurses	13 (48.1)	
	Dietitian	6 (22.2)	
	Others	10 (37.0)	
**Mode of intervention delivery**	Face to face sessions	27 (100)	11 (40.7)
	Group sessions	6 (22.2)	1 (3.7)
	Written materials	12 (44.4)	6 (22.2)
	Telephone sessions	7 (25.9)	-
	Unclear	-	11 (40.7)
**Number of intervention sessions**	1-4 sessions	5 (18.5)	9 (33.3)
	5-9 sessions	10 (37.0)	2 (7.4)
	10-15 sessions	3 (11.1)	1 (3.7)
	>15 sessions	3 (11.1)	-
	Unclear	6 (22.2)	15 (55.5)
**Number of behaviour change techniques (BCT)**	1-2 BCTs	5 (18.5)	12 (44.4)
	3-4 BCTs	10 (37.0)	1 (3.7)
	5-6 BCTs	9 (33.3)	-
	7-9 BCTs	2 (7.4)	-
	10 BCTs	1 (3.7)	-
	Unclear	-	14 (51.8)
**Frequently used behaviour change techniques**	Credible source (9.1)	19 (70.4)	5 (18.5)
	Goal setting (behaviour) (1.1)	17 (62.9)	2 (7.4)
	Information about health consequences (5.1)	8 (29.6)	5 (18.5)
	Self-monitoring of behaviour (2.3)	7 (25.9)	-
	Instruction on how to perform a behaviour (4.1)	7 (25.9)	1 (3.7)
	Action planning (1.4)	6 (22.2)	-

For peer review only

Risk of bias in included studies

Risk of bias assessment is presented in supplementary table 3. Almost half of the included trials (n=13) reported using intention-to-treat (ITT) analysis, while 14 studies did not state ITT procedures. Loss to follow-up ranged from 1.5% to 43%. Not all trials reported sufficient detail to assess risk of bias and these were rated as 'unclear'.

Treatment fidelity

Few studies reported using fidelity checks [24-28] to confirm that interventions were delivered as intended and this raises a question of whether the interventions were delivered as planned, and in a consistent manner.

Effect of interventions

Pooled effect sizes for all outcomes are presented in Table 3 and forest plots are presented in Appendix B.

For peer review only

Table 3: Pooled effects from meta-analysis of multiple health behaviour interventions on CVD-risk and CVD risk factors.

Outcome	N	Pooled effect size		95% confidence interval	P value	I^2 (%)
Systolic blood pressure (mmHg)	12		-1.45	-2.98, 0.09	0.06	68.3
Systolic blood pressure (mmHg) by medication use	6	Medication	-2.03	-4.84, 0.77	0.16	79.9
	6	None	-0.55	-1.69, 0.59	0.35	3.4
Diastolic blood pressure (mmHg)	11		-1.01	-1.91, -0.11	0.03	62.4
Diastolic blood pressure (mmHg) by medication use	6	Medication	-1.34	-1.95, -0.73	<0.001	0.0
	5	None	-0.78	-2.50, 0.93	0.37	73.0
Serum total cholesterol (mmol/L)	10		-0.11	-0.17, -0.05	<0.001	0.0
Serum total cholesterol (mmol/L) by medication use	4	Medication	-0.11	-0.21, -0.02	0.01	0.0
	6	None	-0.11	-0.18, -0.03	0.01	0.0
Smoking (%)	10		-0.01	-0.02, 0.01	0.57	20.2
Body mass index (Kg/m^2)	10		-0.11	-0.25, 0.02	0.10	0.0
Body weight (Kg)	8		-0.87	-1.50, -0.24	0.01	35.4
CVD-risk using SCORE (%)	2		0.12	-0.37, 0.61	0.62	0.0
Systolic blood pressure (mmHg) by theory use	5	Theory	-2.18	-5.92, 1.56	0.25	72.3
	7	None	-1.07	-2.77, 0.63	0.22	69.2
Diastolic blood pressure (mmHg) by theory use	5	Theory	-1.25	-2.43, -0.06	0.04	0.4
	6	None	-0.94	-2.17, 0.29	0.13	75.7
Serum total cholesterol (mmol/L) by theory use	4	Theory	-0.03	-0.15, 0.10	0.68	0.0
	6	None	-0.13	-0.20, -0.07	<0.001	0.0
Body mass index(Kg/m^2) by theory use	5	Theory	-0.15	-0.41, 0.10	0.24	0.0
	5	None	-0.12	-0.30, 0.07	0.22	13.1
Body weight by (Kg) theory use	4	Theory	-0.24	-0.94, 0.45	0.49	0.0
	4	None	-1.33	-2.08, -0.59	<0.001	23.9

N, number of trials; I^2, index of heterogeneity

For peer review only

Changes in CVD risk factors

Blood pressure: twelve trials [23 25 29-38] reported changes in participants' systolic blood pressure (SBP) with no evidence of publication bias (Egger's test, P= 0.82). The weighted mean difference in SBP was -1.45 mm Hg (95% CI -2.98 to 0.09 mm Hg; P= 0.06). Diastolic blood pressure (DBP) was reported in 11 trials [23 25 29-31 33-38], with no evidence of publication bias (Egger's test, P= 0.38). Weighted mean difference in DBP was -1.01 mmHg (-1.91 to -0.11 mm Hg; P= 0.03). Out of the 12 interventions that evaluated blood pressure, four reported that participants in all study groups were taking antihypertensive medications and two reported they were taking unspecified medications. In the subgroup of trials that reported use of medications there was a greater effect on SBP (-2.03 vs. -0.55 mmHg) and DBP (-1.34 vs. -0.78 mmHg) compared to trials that did not report using medications.

Serum total cholesterol: Ten trials [25 29-31 33-35 37-39] evaluated serum total cholesterol and provided sufficient data for analysis (Egger's test, P= 0.53). Serum total cholesterol levels showed a small decrease in favour of intervention (-0.11 mmol/L; 95% -0.17 to -0.05; P<0.001). Three of the trials included in the analysis reported the use of lipid lowering medication and one reported the use of unspecified medication by all study groups. The weighted mean difference for total cholesterol was not different between trials that reported using medication and trials that have not stated using medications (Table 3).

Smoking: Ten studies [23 24 27-30 32 35 40 41] reported smoking prevalence following the intervention. The pooled analysis showed no evidence of reductions in smoking behaviour (RD -0.01 %; 95% CI -0.02 to 0.01; P=0.57). All studies included in the analysis relied on self-reported smoking status and only one [27] reported using smoking cessation medication. There was no evidence of publication bias (P=0.55).

For peer review only

Weight and body mass index (BMI): Ten studies [23 25 29 31 33-38] reported on BMI as an outcome. Egger's test suggested possible publication bias (P=0.049). The weighted mean change was -0.11 kg/m^2 (95% CI -0.25 to 0.02; P=0.10). Fewer studies (n=8) [25 31 33-35 38 41 42] reported on weight changes, showing a reduction of -0.87 kg (CI -1.50 to -0.24 kg; P= 0.01) with no evidence of publication bias (P=0.62).

Dietary behaviour: Thirteen trials [23-28 31 32 39 41-44] reported dietary behaviours as an outcome of the interventions. Outcomes of dietary interventions were measured using diverse methods, therefore, a meta-analysis was not conducted. Trials used a range of dietary self-report instruments to assess dietary behaviour, and none have used additional objective measures. Fruit and vegetable consumption was reported either as portions per day [23-25 41 44], or proportion of participants who met the recommendation for fruits and vegetable intake [24 31 32]. There was no positive effect of the intervention on fruits and vegetable consumption in most of the trials [24 25 32 41], and some trials did report improvement following the intervention [31 44], Fat intake was commonly measured as a dietary outcome either in terms of fat intake per day, [25 31 42 43] or as a fat score [27 32]. All the trials reported reductions in fat intake after the intervention, except Koelewijn-van Loon et al.[32] trial, where there was no significant difference between the intervention and control group.

Physical activity behaviour: Seventeen trials reported changes in physical activity [24-32 34 35 37 38 40 41 44 45]. Physical activity was assessed via self-report. Due to the variety of measurements used, meta-analysis was not feasible. Some trials reported physical activity as the proportion of participants who are physically active [27 29 37 40 44]. Other studies measured physical activity as the number of minutes per week, [25 32] or classified participants based on their weekly exercise [24 26 44]. Eight of these trials [25 27 28 34 35 38 41 44] resulted in an

For peer review only

increase in reported physical activity following the intervention, and nine [24 26 29-31 37 40 42 45] trials concluded that the intervention had no impact on physical activity.

Alcohol consumption: Alcohol consumption was reported as an outcome in seven trials [28 32 38 40-42 44]. However, it was measured differently, which did not allow for pooled effect analysis. Two trials [38 40] reported reductions in alcohol consumption following the interventions, whereas the majority of the studies [28 32 41 42 44] did not find significant reductions in alcohol intake.

Cardiovascular disease risk: Studies used different risk scores to examine the effect of interventions on CVD-risk. Two studies [36 45] used the Framingham risk equation [46] and two studies [28 47] used the Dundee risk score [48]. These trials reported larger CVD-risk reductions in the intervention group compared to the control group. All of these trials had missing data making it not possible to analyse the pooled effect. Four studies [24 32 37 40] used the SCORE risk equation [49], however because of missing data we only included two studies [24 32] in the analysis, both conducted in the Netherlands. There was a non-significant increase in weighted mean difference of 0.12% CVD-risk (95% CI -0.37 to 0.61; P= 0.62).

Study characteristics:

Intervention time and number of sessions: The number of sessions was reported in 20 trials, ranging from three to 56 sessions (median=6 sessions). No significant associations were detected between the number of sessions and SBP (β= -0.16, P=0.67), DBP (β= 0.15, P= 0.59), serum total cholesterol (β=-0.01, P= 0.59), BMI (β= -0.01, P=0.72) and weight (β= -0.13, P=0.72). Ten of the included trials provided enough details to calculate intervention delivery duration, which ranged from 45 to 630 mins (median=285 mins). No significant

associations were detected between intervention duration and SBP (β=-0.02, P=0.17), DBP (β=-0.01, P= 0.36), BMI (β= -0.00, P= 0.79) and weight (β= 0.00, P=0.75). Hence, more sessions and longer intervention duration were not necessarily associated with greater intervention effectiveness.

Theory use: Of the 27 trials included, nine reported using psychological theory (or a combination of two theories) to underpin the intervention. The Transtheoretical Model (TTM) [50] was used in eight trials [25 26 34-38 41], while Social Cognitive Theory (SCT) [51] was used in four [25 26 36 52] interventions.

We tested the extent of theory use using Theory Coding Scheme (TCS) [20] in three ways (supplementary table 4). The first method was based on the use of theory in developing intervention techniques (item 5 in TCS). Only four of nine trials were coded yes for this item. The second method was used to reflect the extent to which each BCT was linked to a theory-relevant construct (items 7 to 9). Only four out of nine trials were coded yes to at least one of these items. The third method was used to reflect the extent to which theory-relevant constructs were targeted by BCTs (items 9 to 11). Only four out of nine trials were coded yes to at least one of these items. We were not able to examine the impact of differing levels of theory use on intervention outcomes due to the small number of trials using theory extensively. However, we were able to test whether studies that merely reported using a theory had greater impact on outcomes using meta-regression. There was no significant association between studies which stated using a theory and SBP (β= -1.15, P= 0.54), DBP (β= -0.37, P= 0.73), serum total cholesterol (β= 0.11, P= 0.17) and BMI (β= -0.06, P= 0.72). Studies that reported using a theory had increased weight outcomes (β= 1.14, P= 0.04, CI= 0.06, 2.22) compared to studies that did not report using a theory.

Effectiveness of specific behaviour change techniques: The number of behaviour change techniques (BCTs) in the intervention group varied, ranging from two to ten BCTs

For peer review only

(median= 4). Behaviour change techniques in the control group were generally poorly described as the majority of trials (n= 14) did not appear to offer any BCTs.

Twenty nine different BCTs were identified from the included trials (supplementary table 2). The most commonly used BCTs in the intervention group were 'credible source' and 'Goal setting (behaviour)', which were used in 19 and 17 trials respectively. In the control group, 'Information about health consequences' and 'Credible source' were most commonly used, which were each used in five interventions.

We tested the potential impact of using specific BCTs on intervention outcomes (table 4). For SBP, one BCT had a significant influence on effect sizes. Interventions employing 'Review of behaviour goal(s)' resulted in an increase in SBP (β=3.96, P= 0.05) than those not using this BCT. For DBP, using 'Information about health consequences' was associated with less change in DBP (β=1.87, P= 0.04). For total cholesterol, there were no BCTs significantly associated with the effectiveness of the interventions. The same was the case for BMI, but for weight, interventions that included 'Action planning' resulted in greater reductions than those that did not (β= -1.22, P= 0.04).

For peer review only

Table 4: Meta-regression results of intervention effects for studies using or not using particular behaviour change techniques.

		BCT included			BCT not included					
**Outcome**	**BCT**	MD	CI	N	MD	CI	N	**β**	**CI**	**P**
**Systolic blood pressure**	1.1 Goal setting (behaviour).	-0.98	-2.58 to 0.63	9	-3.63	-4.93 to -2.34	3	2.16	-2.03 to 6.36	0.28
	1.2 Problem solving.	-3.19	-9.21 to 2.83	3	-1.14	-2.70 to 0.42	9	-1.63	-6.16 to 2.90	0.45
	1.4 Action planning.	-1.99	-5.38 to 1.39	4	-1.29	-3.12 to 0.53	8	-0.60	-4.83 to 3.62	0.76
	**1.5 Review behaviour goal(s)**	**1.76**	**-1.66 to 5.19**	**3**	**-2.19**	**-3.73 to -0.66**	**9**	**3.96**	**0.02 to 7.90**	**0.05**
	5.1 Info. about consequences.	-0.38	-1.99 to 1.23	4	-2.08	-4.31 to 0.14	8	1.54	-2.09 to 5.16	0.37
	9.1 Credible source.	-2.37	-4.28 to -0.46	6	-0.19	-2.85 to 2.47	6	-2.37	-6.10 to 1.35	0.19
	9.2 Pros and cons.	0.16	-3.89 to 4.20	4	-1.98	-3.56 to -0.40	8	2.31	-1.68 to 6.31	0.23
	11.2 Reduce negative emotions.	-1.65	-9.80 to 6.51	3	-1.38	-2.84 0.08	9	-0.22	-5.48 to 5.03	0.93
**Diastolic blood pressure**	1.1 Goal setting (behaviour).	-0.88	-2.04 to 0.28	8	-1.54	-2.33 to -0.74	3	0.77	-1.71 to 3.25	0.50
	1.4 Action planning.	-1.33	-3.99 to 1.33	3	-0.97	-1.98 to 0.03	8	-0.21	-3.02 to 2.59	0.87
	4.1 Instruction on how to perform the behaviour.	-0.15	-1.78 to 1.49	3	-1.53	-2.48 to -0.58	8	1.54	-0.49 to 3.58	0.12
	**5.1 Information about health consequences.**	**0.15**	**-1.32 to 1.63**	**3**	**-1.53**	**-2.29 to -0.78**	**8**	**1.87**	**0.11 to 3.63**	**0.04**
	9.1 Credible source.	-0.91	-2.51 to 0.70	6	-1.32	-1.94 to -0.69	5	0.70	-1.44 to 2.84	0.48
	9.2 Pros and cons.	-1.46	-3.05 to 0.13	3	-0.93	-2.01 to 0.15	8	-0.58	-3.31 to 2.16	0.65
	11.2 Reduced negative emotions.	-2.79	-4.60 to -0.97	3	-0.68	-1.66 to 0.30	8	-2.10	-4.81 to 0.61	0.12
**Serum total cholesterol**	1.1 Goal setting (behaviour).	-0.11	-0.18 to -0.05	7	-0.10	-0.23 to 0.04	3	-0.02	-0.19 to 0.15	0.83
	1.4 Action planning.	-0.02	-0.18 to 0.14	3	-0.12	-0.19 to -0.06	7	0.10	-0.09 to 0.29	0.27
	5.1 Information about health consequences.	-0.11	-0.19 to -0.02	4	-0.11	-0.21 to -0.01	6	0.01	-0.13 to 0.14	0.94
	9.1 Credible source.	-0.09	-0.16 to -0.01	6	-0.14	-0.25 to -0.03	4	0.06	-0.08 to 0.19	0.39
	9.2 Pros and cons.	-0.07	-0.31 to 0.16	3	-0.11	-0.18 to -0.05	7	0.03	-0.18 to 0.24	0.77
**Body mass index**	1.4 Action planning.	-0.33	-0.68 to 0.01	3	-0.07	-0.22 to 0.07	7	-0.26	-0.67 to 0.16	0.20
	5.1 Information about health consequences.	-0.10	-0.33 to 0.14	3	-0.12	-0.28 to 0.04	7	0.03	-0.30 to 0.35	0.86
	9.1 Credible source.	-0.23	-0.43 to -0.02	5	-0.02	-0.20 to 0.16	5	-0.20	-0.51 to 0.10	0.17
	9.2 Pros and cons.	-0.47	-1.29 to 0.34	3	-0.10	-0.24 to 0.03	7	-0.37	-1.31 to 0.57	0.39
**Weight**	1.3 Goal setting (outcome)	-0.97	-2.06 to 0.12	3	-0.78	-1.67 to 0.10	5	-0.18	-1.95 to 1.59	0.81
	**1.4 Action planning.**	**-1.30**	**-1.92 to -0.67**	**4**	**-0.10**	**-0.88 to 0.68**	**4**	**-1.22**	**-2.37 to -0.07**	**0.04**
	4.1 Instruction on how to perform the behaviour.	-0.81	-1.91 to 0.30	3	-0.89	-1.86 to 0.08	5	0.06	-1.77 to 1.89	0.94
	9.1 Credible source.	-0.97	-1.73 to -0.21	5	-0.02	-1.70 to 1.65	3	-0.91	-3.35 to 1.53	0.40
	9.2 Pros and cons.	-0.91	-3.91 to 2.09	3	-0.84	-1.61 to -0.07	5	-0.05	-3.98 to 3.88	0.98

Note: BCT, behaviour change technique; MD, mean difference; CI, 95% confidence interval; N, number of trials; β, meta-regression coefficient

For peer review only

DISCUSSION

This systematic review is among the first to evaluate the impact of theory use and BCTs in MHBC interventions for reducing CVD-risk. The results of this systematic review suggest that MHBC interventions evaluated to date for the primary prevention of CVD may generally have very limited effects in reducing CVD-risk and CVD risk factors in primary care populations.

Previous systematic reviews have investigated the effectiveness of interventions aimed at individual risk factors including diet, physical activity and body weight [6 53]. These reviews generally find that behaviour change interventions in primary care have minor impact on risk factors values. The Cochrane review up to 2011 reported modest reductions in CVD risk factors following MHBC interventions that were wlgithly greater than we report [5]. However, the Cochrane review did not restrict the intervention setting to primary care.

Estimated changes in CVD risk factors should be viewed with caution. The observed effects were heterogeneous, therefore pooled estimates might be questionable. In the present set of trials, the average duration of follow-up was 12 months and changes in risk factors observed may be unlikely to reflect changes occurring over a longer periods. This review found reductions in blood pressure and total cholesterol following intervention, but in some instances this might be mediated by pharmacological treatment. There are clear benefits of drug treatments in lowering blood pressure and cholesterol in primary prevention populations [54 55].

Although this review focused on interventions for the primary prevention population, we also included trials that recruited a small minority of participants with some evidence of CVD.

For peer review only - http://bmjopen.bmj.com/site/about/guidelines.xhtml

Including these trials might have biased the results, as health promotion interventions might have more positive effects in people with established cardiovascular disease [56-58].

In order to account for heterogeneity, we focused on trial level covariates and identified characteristics that might be associated with more favourable outcomes. When coding BCTs, we were limited by the lack of detail provided in reports. We only coded what was explicitly referred to in intervention descriptions and could be fitted to BCT taxonomy definitions.

This review suggested no association between the number of intervention sessions or intervention duration and improved outcomes. Quantity of sessions would not necessarily have a beneficial impact on outcomes unless additional sessions deliver BCTs that effectively influence behaviours. Fewer reports provided sufficient information to permit calculating duration for analysis. Increasing use of the TIDieR checklist [59], requiring intervention reports to detail the number and duration of sessions offered to participants, will be helpful for future reviews.

Our analyses suggested that using certain BCTs has a moderator effect on intervention outcomes. In terms of biomarkers of CVD risk, no BCTs were identified as being particularly likely to influence cholesterol levels, while including review of behaviour goals or information about health consequences appeared to be associated with slightly worse blood pressure outcomes.

“Action planning” was associated with greater weight loss, while “instruction on how to perform the behaviour” was not. Both of these findings differ to those of a previous review [17], perhaps because it focused only on interventions for obese individuals. The previous review

For peer review only

also identified the BCTs of self-monitoring, relapse prevention/problem solving and prompt practice as beneficial to weight loss, but too few of the interventions included in the present review incorporated these BCTs for it to be possible to test their influence. A review of interventions promoting healthy eating and exercise also found that including the BCT of self-monitoring was associated with bigger changes in these behaviours [18]. Therefore, one explanation for the relatively limited effectiveness of the interventions reviewed in the present review is that they failed to include BCTs that were more likely to lead to health-promoting changes. A second possibility is that not all BCTs were delivered as the intervention designers intended. This cannot be ruled out as monitoring of treatment fidelity was rarely described in the included studies.

This review showed no association between the use of psychological theory and improved intervention outcomes. However, only a limited range of theories were employed – mostly TTM and SCT. A previous review also found that interventions based on these theories were not significantly more effective than interventions not explicitly based on theory [13]. A second issue is that the links between the psychological determinants specified by a theory and the BCTs employed in interventions were sometimes poorly articulated, with little evidence cited to justify choice of BCTs to change specific constructs. Furthermore, it was not always clear which BCTs were being used to target which behaviours as part of the MHBC interventions. Both this and previous reviews [13 60] found that reported theory use in intervention design was not as extensive as it could be. It is possible that interventions based on other theories or that more explicitly link theoretical constructs to select BCTs might be more effective.

For peer review only

Limitations

The results of this review must be viewed with caution because of several limitations. First, the majority of trials included were undertaken in Europe (70%) and the United States (14.8%). Declines in CVD mortality and CVD-risk have been observed in these countries, and the results should be considered in the context of these trends. Groups of BCTs may have synergistic effects on behaviour [16]. However, due to the relatively small numbers of studies and under-description of the BCTs used in interventions, it was not possible to explore the impact of clusters of BCTs on CVD risk factors, as too few studies used the same clusters of BCTs and measured the same outcome. Behavioural risk factors were assessed by self-report and so values were subject to social desirability and recall biases. Finally, as this review involved testing for the impact of MHBC interventions and intervention characteristics on intervention outcomes, we are aware of the need to adjust p-values based on the number of testes being made [61]. However, tests were examining independent hypotheses, therefore the p-values were not adjusted.

CONCLUSION

Existing multiple health behaviour change interventions delivered to individual participants in primary care appear to have limited effectiveness at reducing CVD-risk and CVD risk factors over twelve months or longer. Trial reports needs to provide explicit explanation of the intervention theory, content and delivery, including fidelity, in order to understand why an intervention may or may not prove effective. This is essential for future development of effective CVD prevention interventions.

For peer review only

Funding:

MG was supported by the National Institute for Health Research (NIHR) Biomedical Research Centre at Guy's and St Thomas' NHS Foundation Trust and King's College London. SA was supported by the Government of Saudi Arabia.

Contributorship statement:

SA, AW and MG conceptualised and designed the study. SA and MG performed the paper search. SA and LM performed the coding. SA wrote the first draft and all authors have read and made improvements of the contents and the wording.

Competing interests:

There are no competing interests.

Data sharing statement:

No additional data are available.

For peer review only

References

1. WHO. Cardiovascular diseases (CVDs). Accessed August 15, 2016. http://www.who.int/mediacentre/factsheets/fs317/en/.

2. Poortinga W. The prevalence and clustering of four major lifestyle risk factors in an English adult population. *Preventive medicine* 2007;44(2):124-28. doi.org/10.1016/j.ypmed.2006.10.006.

3. Cairney J, Leatherdale ST, Faulkner GE. A longitudinal examination of the interrelationship of multiple health behaviors. *American journal of preventive medicine* 2014;47(3):283-89. doi.org/10.1016/j.amepre.2014.04.019.

4. Khaw K-T, Wareham N, Bingham S, et al. Combined impact of health behaviours and mortality in men and women: the EPIC-Norfolk prospective population study. *PLoS medicine* 2008;5(1):e12. doi.org/10.1371/journal.pmed.0050012.

5. Ebrahim S, Taylor F, Ward K, et al. Multiple risk factor interventions for primary prevention of coronary heart disease. *The Cochrane Library 2011.* doi.org/10.1002/14651858.CD001561.pub3.

6. Fleming P, Godwin M. Lifestyle interventions in primary care Systematic review of randomized controlled trials. *Canadian family physician* 2008;54(12):1706-13.

7. Álvarez-Bueno C, Cavero-Redondo I, Martínez-Andrés M, et al. Effectiveness of multifactorial interventions in primary health care settings for primary prevention of cardiovascular disease: a systematic review of systematic reviews. *Preventive medicine* 2015;76:S68-S75. doi.org/10.1016/j.ypmed.2014.11.028.

8. Krogsbøll LT, Jørgensen KJ, Larsen CG, et al. General health checks in adults for reducing morbidity and mortality from disease: Cochrane systematic review and meta-analysis. *BMJ* 2012;345:e7191. doi.org/10.1136/bmj.e7191.

9. Michie S, Richardson M, Johnston M, et al. The behavior change technique taxonomy (v1) of 93 hierarchically clustered techniques: building an international consensus for the reporting of behavior change interventions. *Annals of behavioral medicine* 2013;46(1):81-95. doi.org/10.1007/s12160-013-9486-6.

For peer review only

10. Campbell M, Fitzpatrick R, Haines A, et al. Framework for design and evaluation of complex interventions to improve health. *BMJ* 2000;321(7262):694-96. doi.org/10.1136/bmj.321.7262.694.

11. Michie S, Johnston M, Francis J, et al. From theory to intervention: mapping theoretically derived behavioural determinants to behaviour change techniques. *Applied psychology* 2008;57(4):660-80. doi.org/10.1111/j.1464-0597.2008.00341.x.

12. Webb T, Joseph J, Yardley L, et al. Using the internet to promote health behavior change: a systematic review and meta-analysis of the impact of theoretical basis, use of behavior change techniques, and mode of delivery on efficacy. *Journal of medical Internet research* 2010;12(1):e4. doi.org/10.2196/jmir.1376.

13. Prestwich A, Sniehotta FF, Whittington C, et al. Does theory influence the effectiveness of health behavior interventions? Meta-analysis. *Health Psychology* 2014;33(5):465. doi.org/10.1037/a0032853.

14. Prestwich A, Webb TL, Conner M. Using theory to develop and test interventions to promote changes in health behaviour: evidence, issues, and recommendations. *Current Opinion in Psychology* 2015;5:1-5. doi.org/10.1016/j.copsyc.2015.02.011.

15. Michie S, Atkins L, West R. The behaviour change wheel: a guide to designing interventions. Great Britain: Silverback Publishing 2015.

16. Michie S, Fixsen D, Grimshaw JM, et al. Specifying and reporting complex behaviour change interventions: the need for a scientific method. *Implement Sci* 2009;4(40):1-6. doi.org/10.1186/1748-5908-4-40.

17. Dombrowski SU, Sniehotta FF, Avenell A, et al. Identifying active ingredients in complex behavioural interventions for obese adults with obesity-related co-morbidities or additional risk factors for co-morbidities: a systematic review. *Health Psychology Review* 2012;6(1):7-32. doi.org/10.1080/17437199.2010.513298. doi.org/10.1080/17437199.2010.513298.

18. Michie S, Abraham C, Whittington C, et al. Effective techniques in healthy eating and physical activity interventions: a meta-regression. *Health Psychology* 2009;28(6):690. doi.org/10.1037/a0016136.

For peer review only

19. Higgins JP, Altman DG, Gøtzsche PC, et al. The Cochrane Collaboration's tool for assessing risk of bias in randomised trials. *BMJ* 2011;343. doi.org/10.1136/bmj.d5928.

20. Michie S, Prestwich A. Are interventions theory-based? Development of a theory coding scheme. *Health Psychology* 2010;29(1):1. doi.org/10.1037/a0016939.

21. Bishop FL, Fenge-Davies AL, Kirby S, et al. Context effects and behaviour change techniques in randomised trials: A systematic review using the example of trials to increase adherence to physical activity in musculoskeletal pain. *Psychology & health* 2015;30(1):104-21. doi.org/10.1080/08870446.2014.953529.

22. Egger M, Smith GD, Schneider M, et al. Bias in meta-analysis detected by a simple, graphical test. *BMJ* 1997;315(7109):629-34.

23. Mendis S, Johnston SC, Fan W, et al. Cardiovascular risk management and its impact on hypertension control in primary care in low-resource settings: a cluster-randomized trial. *Bulletin of the World Health Organization* 2010;88(6):412-19.

24. Lakerveld J, Bot SD, Chinapaw MJ, et al. Motivational interviewing and problem solving treatment to reduce type 2 diabetes and cardiovascular disease risk in real life: a randomized controlled trial. *Int J Behav Nutr Phys Act* 2013;10(47):10.1186. doi.org/10.1186/1479-5868-10-47.

25. Hardcastle SJ, Taylor AH, Bailey MP, et al. Effectiveness of a motivational interviewing intervention on weight loss, physical activity and cardiovascular disease risk factors: a randomised controlled trial with a 12-month post-intervention follow-up. *Int J Behav Nutr Phys Act* 2013;10(40):1-16. doi.org/10.1186/1479-5868-10-40.

26. Parra-Medina D, Wilcox S, Salinas J, et al. Results of the Heart Healthy and Ethnically Relevant Lifestyle trial: a cardiovascular risk reduction intervention for African American women attending community health centers. *American journal of public health* 2011;101(10):1914-21. doi.org/ 10.2105/AJPH.2011.300151.

27. Harting J, van Assema P, van Limpt P, et al. Cardiovascular prevention in the Hartslag Limburg project: effects of a high-risk approach on behavioral risk factors in a general practice population. *Preventive medicine* 2006;43(5):372-78. doi.org/10.1016/j.ypmed.2006.06.016.

For peer review only

28. OXCHECK. Effectiveness of health checks conducted by nurses in primary care: final results of the OXCHECK study. *BMJ* 1995:1099-104. doi.org/10.1136/bmj.310.6987.1099.

29. Knutsen SF, Knutsen R. The Tromsø Survey: the Family Intervention study—the effect of intervention on some coronary risk factors and dietary habits, a 6-year follow-up. Preventive medicine 1991;20(2):197-212. doi.org/10.1016/0091-7435(91)90020-5.

30. Meland E, Lærum E, Ulvik RJ. Effectiveness of two preventive interventions for coronary heart disease in primary care. *Scandinavian journal of primary health care* 1997;15(1):57-63. doi.org/10.3109/02813439709043432.

31. Sartorelli DS, Sciarra EC, Franco LJ, et al. Beneficial effects of short-term nutritional counselling at the primary health-care level among Brazilian adults. *Public health nutrition* 2005;8(07):820-25. doi.org/10.1079/PHN2005737

32. Koelewijn-van Loon MS, van der Weijden T, van Steenkiste B, et al. Involving patients in cardiovascular risk management with nurse-led clinics: a cluster randomized controlled trial. *Canadian Medical Association Journal* 2009;181(12):E267-E74. doi.org/10.1503/cmaj.081591.

33. Brett T, Arnold-Reed D, Phan C, et al. The Fremantle Primary Prevention Study: a multicentre randomised trial of absolute cardiovascular risk reduction. *Br J Gen Pract* 2012;62(594):e22-e28. doi.org/ 10.3399/bjgp12X616337.

34. Steptoe A, Day S, Doherty S, et al. Behavioural counselling in general practice for the promotion of healthy behaviour among adults at increased risk of coronary heart disease: randomised trialCommentary: Treatment allocation by the method of minimisation. *BMJ* 1999;319(7215):943-48.

35. Eriksson MK, Franks PW, Eliasson M. A 3-year randomized trial of lifestyle intervention for cardiovascular risk reduction in the primary care setting: the Swedish Björknäs study. *PloS one* 2009;4(4):e5195. doi.org/10.1371/journal.pone.0005195.

36. Ma J, Berra K, Haskell WL, et al. Case management to reduce risk of cardiovascular disease in a county health care system. *Archives of internal medicine* 2009;169(21):1988-95. doi.org//10.1001_archinternmed.2009.381.

For peer review only

37. Tiessen AH, Smit AJ, Broer J, et al. Randomized controlled trial on cardiovascular risk management by practice nurses supported by self-monitoring in primary care. *BMC family practice* 2012;13(1):1. doi.org//10.1186/1471-2296-13-90.

38. Drevenhorn E, Bengtson A, Nilsson PM, et al. Consultation training of nurses for cardiovascular prevention–a randomized study of 2 years duration. *Blood pressure* 2012;21(5):293-99. doi.org/10.3109/08037051.2012.680734.

39. Baron JA, Gleason R, Crowe B, et al. Preliminary trial of the effect of general practice based nutritional advice. *Br J Gen Pract* 1990;40(333):137-41.

40. Kranjčević K, Marković BB, Lalić DI, et al. Is a targeted and planned GP intervention effective in cardiovascular disease prevention? A randomized controlled trial. *Medical science monitor: international medical journal of experimental and clinical research* 2014;20:1180. doi.org/10.12659/MSM.890242.

41. Harris MF, Fanaian M, Jayasinghe UW, et al. A cluster randomised controlled trial of vascular risk factor management in general practice. *Med J Aust* 2012;197(7):387-93. doi.org/10.5694/mja12.10313.

42. Korhonen M, Kastarinen M, Uusitupa M, et al. The effect of intensified diet counseling on the diet of hypertensive subjects in primary health care: a 2-year open randomized controlled trial of lifestyle intervention against hypertension in eastern Finland. *Preventive medicine* 2003;36(1):8-16. doi.org/10.1006/pmed.2002.1120.

43. Nilsson PM, Lindholm LH, Scherstén BF. Life style changes improve insulin resistance in hyperinsulinaemic subjects: a one-year intervention study of hypertensives and normotensives in Dalby. *Journal of hypertension* 1992;10(9):1071-78.

44. Avram C, Iurciuc M, Craciun L, et al. Dietary and physical activity counseling in high-risk asymptomatic patients with metabolic syndrome–A primary care intervention. *Journal of Food, Agriculture & Environment* 2011;9(3&4):16-19.

45. Lindholm LH, Ekbom T, Dash C, et al. The impact of health care advice given in primary care on cardiovascular risk. *BMJ* 1995;310(6987):1105-09.

46. Kannel WB, McGee D, Gordon T. A general cardiovascular risk profile: the Framingham Study. *The American journal of cardiology* 1976;38(1):46-51.

For peer review only

47. Wood D, Kinmonth A, Davies G, et al. Randomised controlled trial evaluating cardiovascular screening and intervention in general practice: principal results of British family heart study. *BMJ* 1994;308(6924):313-20. doi.org/10.1136/bmj.308.6924.313

48. Tunstall-Pedoe H. The Dundee coronary risk-disk for management of change in risk factors. *BMJ* 1991;303(6805):744-47.

49. Conroy R, Pyörälä K, Fitzgerald Ae, et al. Estimation of ten-year risk of fatal cardiovascular disease in Europe: the SCORE project. *European heart journal* 2003;24(11):987-1003. http://dx.doi.org/10.1016/S0195-668X(03)00114-3.

50. Prochaska JO, Norcross JC. Stages of change. *Psychotherapy: Theory, research, practice, training* 2001;38(4):443.

51. Bandura A. *Social foundations of thought and action: A social cognitive theory*: Prentice-Hall, Inc, 1986.

52. Vetter ML, Wadden TA, Chittams J, et al. Effect of lifestyle intervention on cardiometabolic risk factors: results of the POWER-UP trial. *International Journal of Obesity* 2013;37:S19-S24. doi.org/10.1038/ijo.2013.92.

53. Booth HP, Prevost TA, Wright AJ, et al. Effectiveness of behavioural weight loss interventions delivered in a primary care setting: a systematic review and meta-analysis. *Family practice* 2014;31(6):643-53. doi.org/10.1093/fampra/cmu064.

54. Trialists CT. Efficacy and safety of cholesterol-lowering treatment: prospective meta-analysis of data from 90 056 participants in 14 randomised trials of statins. *The Lancet* 2005;366(9493):1267-78. doi.org/10.1016/S0140-6736(05)67394-1.

55. Taylor F, Huffman MD, Macedo AF, et al. Statins for the primary prevention of cardiovascular disease. *The Cochrane Library* 2013. doi.org/10.1002/14651858.CD004816.pub4.

56. Oldridge NB, Guyatt GH, Fischer ME, et al. Cardiac rehabilitation after myocardial infarction: combined experience of randomized clinical trials. *JAMA* 1988;260(7):945-50. doi.org/10.1001/jama.1988.03410070073031.

57. Taylor RS, Brown A, Ebrahim S, et al. Exercise-based rehabilitation for patients with coronary heart disease: systematic review and meta-analysis of randomized

controlled trials. *The American journal of medicine* 2004;116(10):682-92. doi.org/10.1016/j.amjmed.2004.01.009.

58. Mullen PD, Mains DA, Velez R. A meta-analysis of controlled trials of cardiac patient education. *Patient education and counseling* 1992;19(2):143-62. .doi.org/10.1016/0738-3991(92)90194-N.

59. Hoffmann TC, Glasziou PP, Boutron I, et al. Better reporting of interventions: template for intervention description and replication (TIDieR) checklist and guide. *BMJ* 2014;348:g1687. doi.org/10.1136/bmj.g1687.

60. Michie S, Jochelson K, Markham WA, et al. Low income groups and behaviour change interventions: a review of intervention content, effectiveness and theoretical frameworks. *Journal of Epidemiology and Community Health* 2009:jech. 2008.078725. doi.org/10.1136/jech.2008.078725.

61. Bland JM, Altman DG. Multiple significance tests: the Bonferroni method. *BMJ* 1995;310(6973):170. doi.org/10.1136/bmj.310.6973.170.

For peer review only

PRISMA 2009 Checklist

Section/topic	#	Checklist item	Reported on page #
**TITLE**			
Title	1	Identify the report as a systematic review, meta-analysis, or both.	1
**ABSTRACT**			
Structured summary	2	Provide a structured summary including, as applicable: background; objectives; data sources; study eligibility criteria, participants, and interventions; study appraisal and synthesis methods; results; limitations; conclusions and implications of key findings; systematic review registration number.	2
**INTRODUCTION**			
Rationale	3	Describe the rationale for the review in the context of what is already known.	3 & 4
Objectives	4	Provide an explicit statement of questions being addressed with reference to participants, interventions, comparisons, outcomes, and study design (PICOS).	4 - 6
**METHODS**			
Protocol and registration	5	Indicate if a review protocol exists, if and where it can be accessed (e.g., Web address), and, if available, provide registration information including registration number.	N/A
Eligibility criteria	6	Specify study characteristics (e.g., PICOS, length of follow-up) and report characteristics (e.g., years considered, language, publication status) used as criteria for eligibility, giving rationale.	6 & 7
Information sources	7	Describe all information sources (e.g., databases with dates of coverage, contact with study authors to identify additional studies) in the search and date last searched.	7
Search	8	Present full electronic search strategy for at least one database, including any limits used, such that it could be repeated.	Appendix A
Study selection	9	State the process for selecting studies (i.e., screening, eligibility, included in systematic review, and, if applicable, included in the meta-analysis).	7
Data collection process	10	Describe method of data extraction from reports (e.g., piloted forms, independently, in duplicate) and any processes for obtaining and confirming data from investigators.	7 & 8
Data items	11	List and define all variables for which data were sought (e.g., PICOS, funding sources) and any assumptions and simplifications made.	7 & 8
Risk of bias in individual studies	12	Describe methods used for assessing risk of bias of individual studies (including specification of whether this was done at the study or outcome level), and how this information is to be used in any data synthesis.	7 & 14

For peer review only

Summary measures	13	State the principal summary measures (e.g., risk ratio, difference in means).	8 & 9
Synthesis of results	14	Describe the methods of handling data and combining results of studies, if done, including measures of consistency (e.g., I^2) for each meta-analysis.	8 & 9
Risk of bias across studies	15	Specify any assessment of risk of bias that may affect the cumulative evidence (e.g., publication bias, selective reporting within studies).	9
Additional analyses	16	Describe methods of additional analyses (e.g., sensitivity or subgroup analyses, meta-regression), if done, indicating which were pre-specified.	8 & 9
**RESULTS**			
Study selection	17	Give numbers of studies screened, assessed for eligibility, and included in the review, with reasons for exclusions at each stage, ideally with a flow diagram.	10 and figure 1
Study characteristics	18	For each study, present characteristics for which data were extracted (e.g., study size, PICOS, follow-up period) and provide the citations.	Supplementary table 1&2
Risk of bias within studies	19	Present data on risk of bias of each study and, if available, any outcome level assessment (see item 12).	Supplementary table 3
Results of individual studies	20	For all outcomes considered (benefits or harms), present, for each study: (a) simple summary data for each intervention group (b) effect estimates and confidence intervals, ideally with a forest plot.	Appendix B
Synthesis of results	21	Present results of each meta-analysis done, including confidence intervals and measures of consistency.	15
Risk of bias across studies	22	Present results of any assessment of risk of bias across studies (see Item 15).	16-20
Additional analysis	23	Give results of additional analyses, if done (e.g., sensitivity or subgroup analyses, meta-regression [see Item 16]).	19 - 21
**DISCUSSION**			
Summary of evidence	24	Summarize the main findings including the strength of evidence for each main outcome; consider their relevance to key groups (e.g., healthcare providers, users, and policy makers).	22
Limitations	25	Discuss limitations at study and outcome level (e.g., risk of bias), and at review-level (e.g., incomplete retrieval of identified research, reporting bias).	25
Conclusions	26	Provide a general interpretation of the results in the context of other evidence, and implications for future research.	25
**FUNDING**			
Funding	27	Describe sources of funding for the systematic review and other support (e.g., supply of data); role of funders for the systematic review.	25

For peer review only

From: Moher D, Liberati A, Tetzlaff J, Altman DG, The PRISMA Group (2009). Preferred Reporting Items for Systematic Reviews and Meta-Analyses: The PRISMA Statement. PLoS Med 6(7): e1000097. doi:10.1371/journal.pmed100009

For peer review only

For peer review only

Appendix A

Search strategy

CENTRAL search strategy

ID	Search	Hits
#1	MeSH descriptor CARDIOVASCULAR DISEASES this term only	480
#2	MeSH descriptor CORONARY DISEASE explode all trees	356
#3	cardiovascular in All Text	2052
#4	(coronary in All Text near/3 disease* in All Text)	9
#5	(heart in All Text near/3 disease* in All Text)	11
#6	MeSH descriptor HYPERTENSION this term only	643
#7	hypertension in All Text	1781
#8	(atherosclerosis in All Text or arteriosclerosis in All Text)	258
#9	(hyperlipidaemia in All Text or hyperlipidemia in All Text)	224
#10	MeSH descriptor ARTERIOSCLEROSIS explode all trees	79
#11	MeSH descriptor CHOLESTEROL explode trees all trees	209
#12	MeSH descriptor HYPERLIPIDEMIA explode all trees	33
#13	cholesterol in All Text	630
#14	multiple next risk next factor* in All Text	51
#15	coronary next risk next factor* in All Text	30
#16	(#1 or #2 or #3 or #4 or #5 or #6 or #7 or #8 or #9 or #10)	3105
#17	(#11 or #12 or #13 or #14 or #15)	682
#18	(#16 or #17)	3234
#19	MeSH descriptor HEALTH EDUCATION explode all trees	630
#20	MeSH descriptor HEALTH PROMOTION explode all trees	191
#21	MeSH descriptor HEALTH BEHAVIOR explode all trees	215
#22	MeSH descriptor PRIMARY PREVENTION this term only	1021
#23	MeSH descriptor COUNSELLING this term only	237
#24	counsel* in All Text	1186
#25	(health in All Text near/3 educat* in All Text)	31
#26	(patient in All Text near/3 educat* in All Text)	20
#27	(education* in All Text near/3 program* in All Text)	23
#28	(health in All Text near/3 promotion* in All Text)	2
#29	(health in All Text near/3 behaviour* in All Text)	11
#30	(health in All Text near/3 behavior* in All Text)	9
#31	primary next prevention in All Text	379
#32	(multiple next risk in All Text near/3 intervention* in All Text)	6
#33	(multifactor* in All Text near/3 intervention* in All Text)	9
#34	(multifactor* in All Text near/3 prevention in All Text)	1
#35	(risk next factor* in All Text near/3 reduc* in All Text)	10
#36	(risk next factor* in All Text near/3 manag* in All Text)	20
#37	(risk next factor* in All Text near/3 intervent* in All Text)	49

For peer review only

#38 (lifestyle in All Text near/3 intervention* in All Text) 34
#39 (lifestyle in All Text near/3 advice in All Text) 6
#40 (life-style in All Text near/3 intervention* in All Text) 12
#41 (life-style in All Text near/3 advice in All Text) 2
#42 (life-style in All Text near/3 alter* in All Text) 1
#43 (lifestyle in All Text near/3 alter* in All Text) 5
#44 (lifestyle in All Text near/3 educat* in All Text) 15
#45 (life-style in All Text near/3 educat* in All Text) 5
#46 (life-style in All Text near/3 chang* in All Text) 8
#47 (lifestyle in All Text near/3 chang* in All Text) 18
#48 (behavior* in All Text near/3 chang* in All Text) 24
#49 (behaviour* in All Text near/3 chang* in All Text) 37
#50 (health next care in All Text near/3 advice in All Text) 7
#51 (healthcare in All Text near/3 advice in All Text) 8
#52 nonpharmacologic* in All Text 46
#53 non-pharmacologic* in All Text 562
#54 (#19 or #20 or #21 or #22 or #23 or #24 or #25 or #26 or #27 or #28 or #29) 2311
#55 (#30 or #31 or #32 or #33 or #34 or #35 or #36 or #37 or #38 or #39) 451
#56 (#40 or #41 or #42 or #43 or #44 or #45 or #46 or #47 or #48 or #49 or #50 or #51 or #52 or #53) 646
#57 (#54 or #55 or #56) 2915
#58 (#18 and #57) 1293

Embase search strategy

1. cardiovascular disease/
2. exp ischemic heart disease/
3. (Coronary adj3 disease$).tw.
4. heart disease$.tw.
5. Hypertension/
6. hypertension.tw.
7. (cardiovascular adj3 (disease$ or fit of fitness)).tw.
8. exp arteriosclerosis/
9. exp hyperlipidemia/
10. hyperlipid?emia.tw.
11. cholesterol.tw.
12. arteriosclero$.tw.
13. atherosclero$.tw.
14. coronary risk factor$.tw.
15. multiple risk factor$.tw.
16. cardiovascular risk factor$.tw.
17. or/1-16
18. exp health education/
19. exp health behavior/

For peer review only

20. primary prevention/
21. exp counseling/
22. (multifactor$ adj5 (intervent$ or prevent$)).tw.
23. ((life-style or life style or lifestyle or healthcare or health care) adj3 (intervention$ or educat$ or advice or alter$ or change$)).tw.
24. primary prevention.tw.
25. (risk factor$ adj3 (reduc$ or manage$ or managing or intervent$ or program$)).tw.
26. (educat$ adj3 (program$ or patient$)).tw.
27. (non pharmacologic$ or nonpharmacologic$).tw.
28. (risk factor$ adj3 modif$).tw.
29. ((lifestyle or life-style or life style) adj3 modif$).tw.
30. exp behavior therapy/
31. (behavi?r$ adj3 (intervention$ or program$ or modif$ or change$ or alter$)).tw.
32. (promot$ adj3 (health or healthcare or health care)).tw.
33. or/18-32
34. 17 and 33
35. random$.ti,ab.
36. factorial$.ti,ab.
37. (crossover$ or cross over$ or cross-over$).ti,ab.
38. placebo$.ti,ab.
39. (double$ adj blind$).ti,ab.
40. (singl$ adj blind$).ti,ab.
41. assign$.ti,ab.
42. allocat$.ti,ab.
43. volunteer$.ti,ab.
44. Crossover Procedure/
45. Double Blind Procedure/
46. Randomized Controlled Trial/
47. Single Blind Procedure/
48. or/35-47
49. exp animal/
50. nonhuman/
51. exp animal experiment/
52. or/49-51
53. exp human/
54. 52 not 53
55. 48 not 54
56. 55 and 34
57. limit 56 to yr="2006 -Current"

Medline search strategy

1. Cardiovascular Diseases/
2. exp coronary disease/
3. Hypertension/

For peer review only

4. exp Arteriosclerosis/
5. exp Hyperlipidemia/
6. (cardiovascular adj3 disease$).tw.
7. (cardiovascular adj3 (fit or fitness)).tw.
8. (Coronary adj3 disease$).tw.
9. heart disease$.tw.
10. hypertension.tw.
11. hyperlipid?emia.tw.
12. cholesterol.tw.
13. atherosclerosis.tw.
14. arteriosclerosis.tw.
15. coronary risk factor$.tw.
16. multiple risk factor$.tw.
17. cardiovascular risk factor$.tw.
18. or/1-17
19. health promotion/
20. exp health education/
21. exp health behavior/
22. exp counseling/
23. Primary Prevention/
24. (multifactor$ adj5 (intervent$ or prevent$)).tw.
25. ((lifestyle or life-style) adj3 (intervention$ or educat$ or advice$ or alter$ or change$)).tw.
26. ((lifestye or life-style or behavior?r$) adj3 (intervention$ or educat$ or advice$ or alter$ or change$)).tw.
27. ((healthcare or health care) adj3 advice).tw.
28. primary prevention.tw.
29. (risk factor$ adj3 (reduc$ or manage$ or managing or intervent$ or program$)).tw.
30. (educat$ adj3 (program$ or patient$)).tw.
31. ((health or healthcare or health care) adj3 (educat$ or advice or promot$)).tw.
32. (nonpharmacologic$ or non-pharmacologic$).tw.
33. ((lifestyle or life style or life-style or behavio?r$ or risk factor$) adj3 modif$).tw.
34. or/19-33
35. 18 and 34
36. randomized controlled trial.pt.
37. controlled clinical trial.pt.
38. Randomized controlled trials/
39. random allocation.sh.
40. double blind method.sh.
41. single-blind method.sh.
42. or/36-41
43. clinical trial.pt.
44. exp Clinical trial/
45. (clin$ adj25 trial$).ti,ab.

For peer review only

46. ((singl$ or doubl$ or trebl$ or tripl$) adj (blind$ or mask$)).ti,ab.
47. placebos.sh.
48. placebo$.ti,ab.
49. random$.ti,ab.
50. research design.sh.
51. or/43-50
52. exp animal/ not humans/
53. 42 or 51
54. 53 not 52
55. 54 and 35

PsycINFO search strategy:

1. cardiovascular disease.mp.
2. hypertension.mp.
3. (Coronary adj3 disease$).mp.
4. heart disease$.mp.
5. (cardiovascular adj3 (disease$ or fit of fitness)).mp. [mp=title, abstract, heading word, table of contents, key concepts, original title, tests & measures]
6. exp Arteriosclerosis/
7. hyperlipid?emia.mp.
8. cholesterol.mp.
9. arteriosclero$.mp.
10. atherosclero$.mp.
11. coronary risk factor$.mp.
12. multiple risk factor$.mp.
13. cardiovascular risk factor$.mp.
14. or/1-13
15. exp health education/
16. exp health education/
17. exp health promotion/
18. exp preventive medicine/
19. exp counseling/
20. primary prevention.mp.
21. (multifactor$ adj5 (intervent$ or prevent$)).mp.
22. behavior change.mp.
23. exp Obesity/ or exp Food Intake/ or diet intervention.mp. or exp Weight Loss/ or exp Diets/ or exp Overweight/ or exp Weight Control/ or exp Nutrition/
24. exp Nicotine/ or exp Tobacco Smoking/ or exp Smoking Cessation/ or cigarette.mp. or exp Drug Dependency/
25. exp Alcohol Drinking Patterns/ or exp Drinking Behavior/ or exp Alcohol Drinking Attitudes/ or exp Binge Drinking/ or drinking.mp.
26. exp Physical Activity/ or exp Intervention/ or exp Exercise/ or exp Physical Fitness/ or exp Motor Performance/ or physical training.mp.
27. 23 and 24
28. 23 and 25

For peer review only

29. 23 and 26
30. 24 and 25
31. 24 and 26
32. 25 and 26
33. ((life-style or life style or lifestyle or healthcare or health care) adj3 (intervention$ or educat$ or advice or alter$ or change$)).mp.
34. primary prevention.mp.
35. (risk factor$ adj3 (reduc$ or manage$ or managing or intervent$ or program$)).sh.
36. (educat$ adj3 (program$ or patient$)).mp.
37. (non pharmacologic$ or nonpharmacologic$).mp.
38. (risk factor$ adj3 modif$).mp.
39. ((lifestyle or life-style or life style) adj3 modif$).mp.
40. (behavi?r$ adj3 (intervention$ or program$ or modif$ or change$ or alter$)).mp.
41. (promot$ adj3 (health or healthcare or health care)).mp.
42. 15 or 16 or 17 or 18 or 19 or 20 or 21 or 22 or 27 or 28 or 29 or 30 or 31 or 32 or 33 or 34 or 35 or 36 or 37 or 38 or 39 or 40 or 41
43. 14 and 42
44. random$.ti,ab.
45. factorial$.ti,ab.
46. (crossover$ or cross over$ or cross-over$).ti,ab.
47. placebo$.ti,ab.
48. (double$ adj blind$).ti,ab.
49. (singl$ adj blind$).ti,ab.
50. assign$.ti,ab.
51. allocat$.ti,ab.
52. volunteer$.ti,ab.
53. ("double-blind" or "random* assigned" or control).mp.
54. treatment effectiveness evaluation.mp.
55. treatment outcome clinical trial$.mp.
56. (controlled trial$ and clinical trial$).mp. [mp=title, abstract, heading word, table of contents, key concepts, original title, tests & measures]
57. 44 or 45 or 46 or 47 or 48 or 49 or 50 or 51 or 52 or 53 or 54 or 55 or 56
58. 43 and 57

For peer review only

Appendix B

Forest plots of pooled effect of multiple behaviour interventions on intervention outcomes.

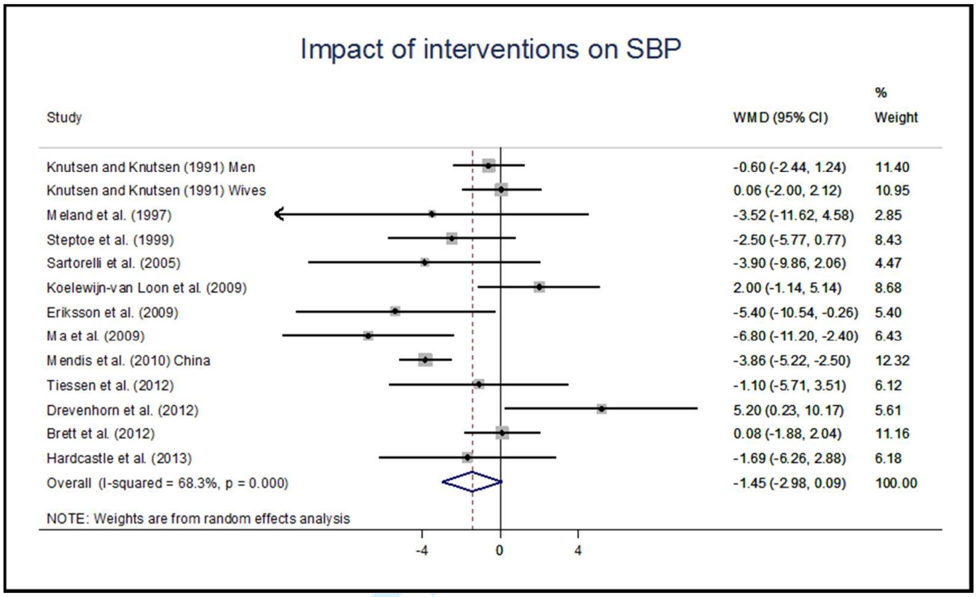

Pooled effect of multiple behaviour interventions on systolic blood pressure (mmHg). Random effects model used. MWD= mean weighted difference. 95% CI = 95% confidence intervals.

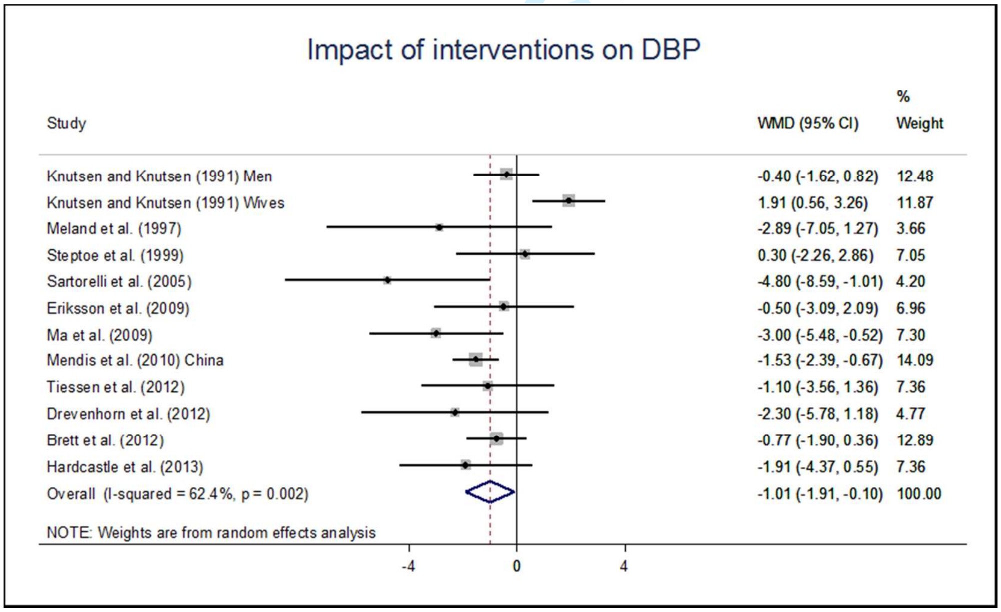

Pooled effect of multiple behaviour interventions on diastolic blood pressure (mmHg). Random effects model used. MWD= mean weighted difference. 95% CI = 95% confidence intervals.

For peer review only - http://bmjopen.bmj.com/site/about/guidelines.xhtml

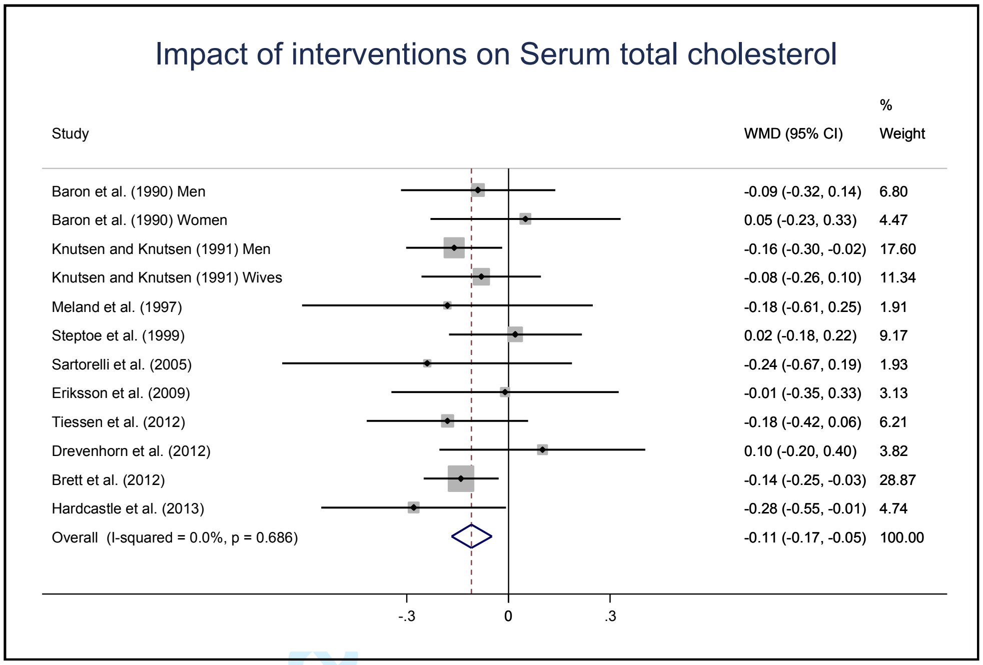

Pooled effect of multiple behaviour interventions on serum total cholesterol (mmol/L). Random effects model used. MWD= mean weighted difference. 95% CI = 95% confidence intervals.

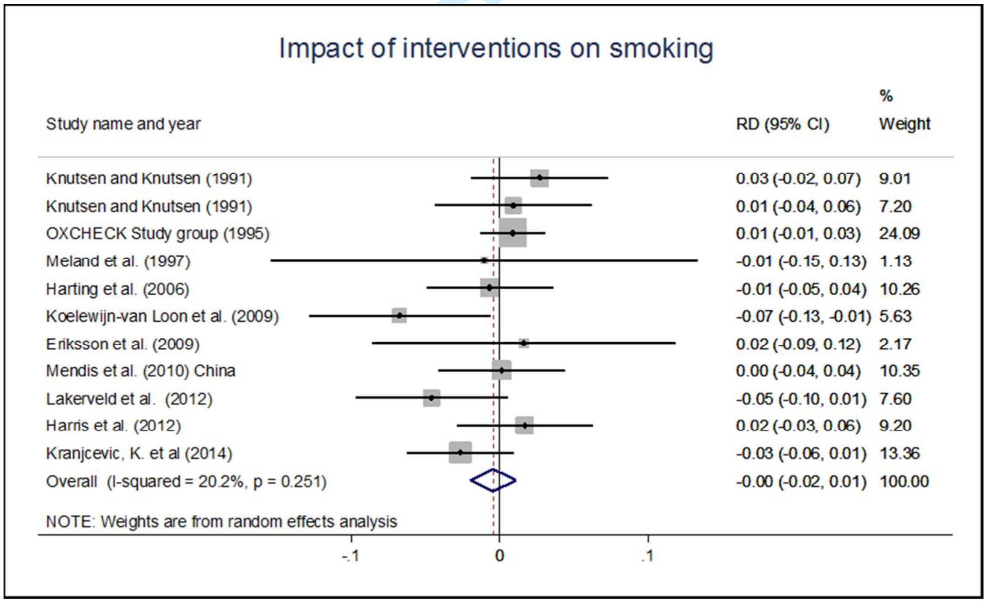

Pooled effect of multiple behaviour interventions on smoking prevalence. Random effects models used. RD= risk difference. 95% CI = 95% confidence intervals.

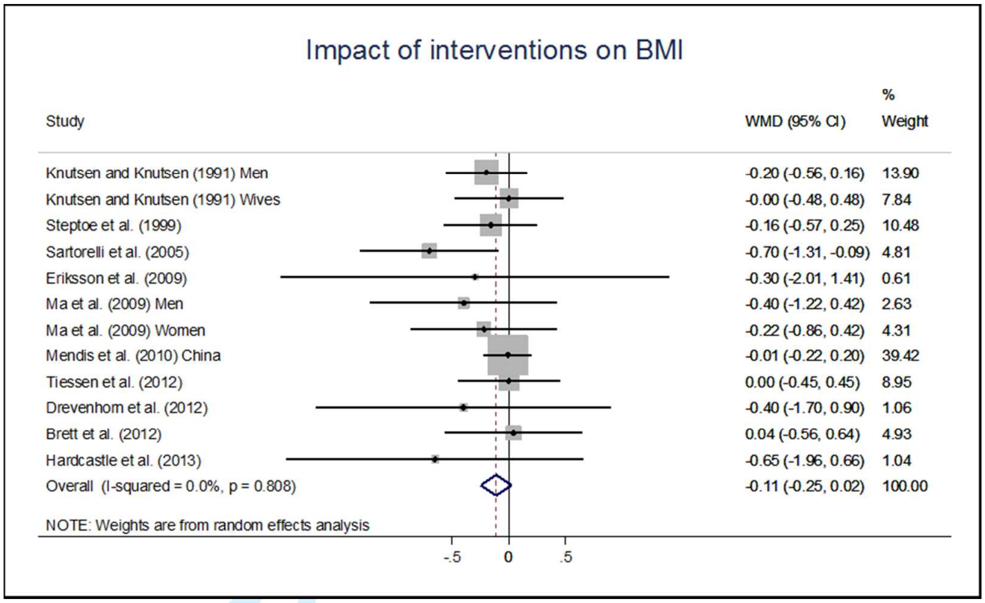

Pooled effect of multiple behaviour interventions on body mass index (Kg/m^2). Random effects model used. MWD= mean weighted difference. 95% CI = 95% confidence intervals.

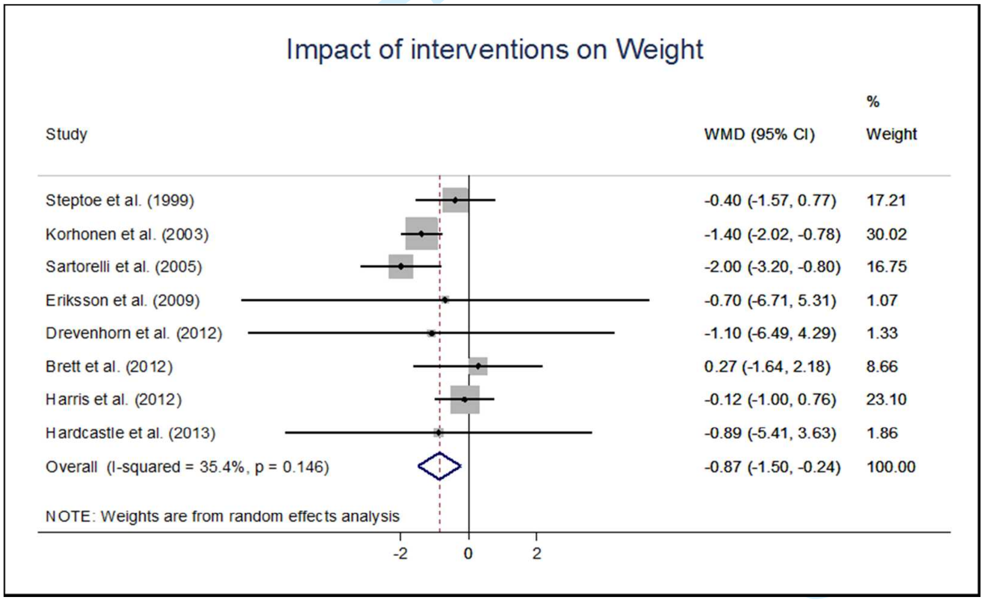

Pooled effect of multiple behaviour interventions on weight (Kg). Random effects model used. MWD= mean weighted difference. 95% CI = 95% confidence intervals.

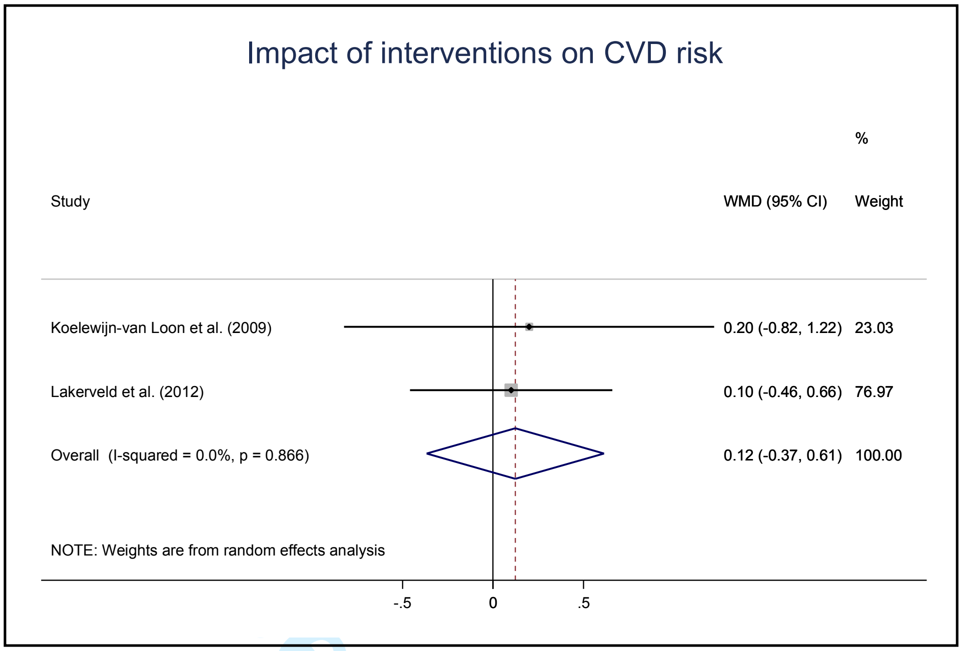

Pooled effect of multiple behaviour interventions on cardiovascular risk (SCORE). Random effect models used. MWD= mean weighted difference. 95% CI = 95% confidence intervals.

For peer review only

Supplementary table 1: Trial characteristics of included studies.

Study (Year)	Country	Number of Participants	Selection criteria	Targeted behaviours	Follow-up duration	Intervention reported outcomes
**Kranjčević, et al.** [1]	Croatia	1957	Men and women, aged ≥40.	Diet and PA.	18 months	CVD-risk, weight, BP, cholesterol, smoking, alcohol and PA.
**Vetter, et al.** [2]	United States	390	Men and women, aged≥21 years, BMI= 30-50kg/m^2, elevated waist circumference.	Diet and PA.	2 years	Weight, BP and cholesterol.
**Lakerveld, et al.** [3]	Netherlands	622	Men and women, aged: 30-50 years.	Diet, PA and smoking.	12 months	CVD-risk, smoking, diet and PA.
**Hardcastle, et al.** [4]	United Kingdom	334	Men and women, aged 18-65 years and have at least one CVD risk factor.	Diet and PA.	18 months	Weight, BP, cholesterol, diet and PA.
**Tiessen, et al.** [5]	Netherlands	201	Men aged: 50-75 years old and women aged: 55-75 years and CVD-risk (SCORE) ≥ 5%.	PA, diet and smoking.	12 months	CVD-risk, weight, BP, cholesterol, smoking and PA.
**Parra-Medina, et al.** [6]	United States	266	African-American women, aged≥35 years, baseline BP <160/95.	PA and diet.	12 months	Diet and PA.
**Drevenhorn, et al.** [7]	Sweden	153	Hypertensive patients, men and women aged <75 years, elevated BP, BMI ≥ 25, serum cholesterol ≥ 6.5 and/or serum triglycerides ≥ 2.3 and not reporting regular PA.	Smoking, alcohol, weight, PA and stress	2 years	Weight, BP, cholesterol, alcohol and PA.
**Brett, et al.** [8]	Australia	1200	Men and women aged 40-80 years, without a history of CVD.	Diet, PA and smoking.	12 months	CVD-risk, weight, BP and cholesterol.
**Harris, et al.** [9]	Australia	814	Men and women, aged 40-55 years with recorded diagnosis of hypertension and/or hyperlipidaemia or aged 56-64 years.	Diet, PA, smoking and alcohol.	12 months	CVD-risk, weight, BP, cholesterol, smoking, alcohol, diet and PA.
**Mendis, et al.** [10]	China	1209	Men and women aged 30-70 years with SBP in the range (140-179 mmHg).	Smoking cessation, PA and diet.	12 months	Weight, BP, smoking and diet.
	Nigeria	1188				
**Koelewijn-van Loon, et al.** [11]	Netherlands	615	One or more of the following: BP≥ 140 or on treatment for high BP; total cholesterol ≥ 6.5 or on treatment for high cholesterol; smoker aged ≥ 50 years (men) or ≥ 55 years (women); diabetes; a family history of CVD; and obese.	Smoking status, diet, PA and alcohol use.	12 months	CVD-risk, BP, cholesterol, smoking, diet and PA.
**Eriksson, et al.** [12]	Sweden	151	Men and women aged 18–65 years with hypertension, dyslipidaemia, type 2 diabetes or obesity.	Diet and PA.	3 years	Weight, BP, cholesterol, smoking and PA.
**Phelan, et al.** [13]	United States	224	Men and women aged 18–65 years and BMI of 30–45 kg/m^2.	Diet and PA.	12 months	Weight, BP, cholesterol and diet.

For peer review only

**Harting, et al.** [14]	Netherlands	1300	Men and women who have a greater than 20% risk (Framingham) of incurring a CVD event within 10 years.	Diet, PA and smoking.	18 months	Smoking, diet and PA.
**Korhonen, et al.** [15]	Finland	715	Men and women aged 25–74 years, with systolic BP 140–179 and/or diastolic BP 90–109 and/or on treatment for hypertension.	Diet and alcohol (also PA and smoking).	24 months	Weight, BP, cholesterol, alcohol, diet and PA.
**Baron, et al.** [16]	United Kingdom	368	Men and women aged 25 – 60 years.	Diet mainly, but changes in PA, alcohol and smoking were also mentioned.	12 months	Cholesterol and diet.
**Knutsen and Knutsen** [17]	Norway	1373 men, 1143 wives	Men aged 20 – 54 years and women aged 20-49 years, with no known CHD at baseline.	Diet changes, PA and smoking cessation.	6 years	CVD-risk, weight, BP, cholesterol, smoking and PA.
**Nilsson, et al.** [18]	Sweden	86	Men and women, born during the period 1925 – 1952, treated hypertensives.	Diet, smoking, PA and alcohol.	12 months	Weight, BP, cholesterol, smoking and diet.
**Wood, et al.** [19]	United Kingdom	7460 men, 5012 women	Men aged 40-59 and their families.	Smoking, weight, diet, alcohol, and PA.	12 months	CVD-risk, weight, BP, cholesterol and smoking.
**OXCHECK Study Group** [20]	United Kingdom	5559	Men and women aged 35-64.	Diet, smoking and PA.	3 years	CVD-risk, weight, BP, cholesterol, alcohol, diet, PA and smoking.
**Lindholm, et al.** [21]	Sweden	681	Men and women aged 30-59 years, had a moderate hyperlipidaemia, and at least two CVD risk factors.	Diet, smoking and PA.	18 months	CVD-risk, weight, BP, cholesterol, PA and smoking.
**Meland, et al.** [22]	Norway	127	Men aged 30 to 59 years.	Diet, smoking and PA.	12 months	CVD-risk, BP, cholesterol, PA and smoking.
**Avram, et al.** [23]	Romania	253	Men and women under 80 years, without history of CVD but defined as high risk individuals.	Diet and PA.	18 months	Weight, alcohol, diet and PA.
**Steptoe, et al.** [24]	United Kingdom	883	Men and women aged 18 – 69, total cholesterol of 6.5-9; smoker, BMI of 25-35 and lack of regular PA.	Smoking, diet and PA.	12 months	Weight, BP, cholesterol, diet and PA.
**Sartorelli, et al.** [25]	Brazil	104	Men and women aged 30-65 years, body mass index of 24-35 kg/m^2, and non-diabetic.	Diet and PA.	12 months	Weight, BP, cholesterol, diet and PA.
**Ma, et al.** [26]	United States	419	Men and women aged 35 to 85 years, had moderately to severely elevated levels of major modifiable CVD risk factors.	PA, diet and stress reduction.	15 months	CVD-risk, weight, BP and cholesterol.
**Tibblin and Åberg** [27]	Sweden	400	Men and women aged 30 - 69 years, on hypertensive drugs	Diet, PA and stress management.	12 months	Weight, BP and cholesterol.

Note: BMI: body mass index, PA: physical activity, BP: blood pressure, CVD: cardiovascular disease

For peer review only

References:

1. Kranjčević K, Marković BB, Lalić DI, et al. Is a targeted and planned GP intervention effective in cardiovascular disease prevention? A randomized controlled trial. Medical science monitor: international medical journal of experimental and clinical research 2014;**20**:1180.

2. Vetter ML, Wadden TA, Chittams J, et al. Effect of lifestyle intervention on cardiometabolic risk factors: results of the POWER-UP trial. International Journal of Obesity 2013;**37**:S19-S24.

3. Lakerveld J, Bot SD, Chinapaw MJ, et al. Motivational interviewing and problem solving treatment to reduce type 2 diabetes and cardiovascular disease risk in real life: a randomized controlled trial. Int J Behav Nutr Phys Act 2013;**10**(47):10.1186.

4. Hardcastle SJ, Taylor AH, Bailey MP, et al. Effectiveness of a motivational interviewing intervention on weight loss, physical activity and cardiovascular disease risk factors: a randomised controlled trial with a 12-month post-intervention follow-up. Int J Behav Nutr Phys Act 2013;**10**(40):1-16.

5. Tiessen AH, Smit AJ, Broer J, et al. Randomized controlled trial on cardiovascular risk management by practice nurses supported by self-monitoring in primary care. BMC family practice 2012;**13**(1):1.

6. Parra-Medina D, Wilcox S, Salinas J, et al. Results of the Heart Healthy and Ethnically Relevant Lifestyle trial: a cardiovascular risk reduction intervention for African American women attending community health centers. American journal of public health 2011;**101**(10):1914-21.

7. Drevenhorn E, Bengtson A, Nilsson PM, et al. Consultation training of nurses for cardiovascular prevention–a randomized study of 2 years duration. Blood pressure 2012;**21**(5):293-99.

8. Brett T, Arnold-Reed D, Phan C, et al. The Fremantle Primary Prevention Study: a multicentre randomised trial of absolute cardiovascular risk reduction. Br J Gen Pract 2012;**62**(594):e22-e28.

9. Harris MF, Fanaian M, Jayasinghe UW, et al. A cluster randomised controlled trial of vascular risk factor management in general practice. Med J Aust 2012;**197**(7):387-93.

10. Mendis S, Johnston SC, Fan W, et al. Cardiovascular risk management and its impact on hypertension control in primary care in low-resource settings: a cluster-randomized trial. Bulletin of the World Health Organization 2010;**88**(6):412-19.

For peer review only

11. Koelewijn-van Loon MS, van der Weijden T, van Steenkiste B, et al. Involving patients in cardiovascular risk management with nurse-led clinics: a cluster randomized controlled trial. Canadian Medical Association Journal 2009;**181**(12):E267-E74.
12. Eriksson MK, Franks PW, Eliasson M. A 3-year randomized trial of lifestyle intervention for cardiovascular risk reduction in the primary care setting: the Swedish Björknäs study. PloS one 2009;**4**(4):e5195.
13. Phelan S, Wadden T, Berkowitz R, et al. Impact of weight loss on the metabolic syndrome. International journal of obesity 2007;**31**(9):1442-48.
14. Harting J, van Assema P, van Limpt P, et al. Cardiovascular prevention in the Hartslag Limburg project: effects of a high-risk approach on behavioral risk factors in a general practice population. Preventive medicine 2006;**43**(5):372-78.
15. Korhonen M, Kastarinen M, Uusitupa M, et al. The effect of intensified diet counseling on the diet of hypertensive subjects in primary health care: a 2-year open randomized controlled trial of lifestyle intervention against hypertension in eastern Finland. Preventive medicine 2003;**36**(1):8-16.
16. Baron JA, Gleason R, Crowe B, et al. Preliminary trial of the effect of general practice based nutritional advice. Br J Gen Pract 1990;**40**(333):137-41.
17. Knutsen SF, Knutsen R. The Tromsø Survey: the Family Intervention study—the effect of intervention on some coronary risk factors and dietary habits, a 6-year follow-up. Preventive medicine 1991;**20**(2):197-212.
18. Nilsson PM, Lindholm LH, Scherstén BF. Life style changes improve insulin resistance in hyperinsulinaemic subjects: a one-year intervention study of hypertensives and normotensives in Dalby. Journal of hypertension 1992;**10**(9):1071-78.
19. Wood D, Kinmonth A, Davies G, et al. Randomised controlled trial evaluating cardiovascular screening and intervention in general practice: principal results of British family heart study. Bmj 1994;**308**(6924):313-20.
20. OXCHECK. Effectiveness of health checks conducted by nurses in primary care: final results of the OXCHECK study. BMJ: British Medical Journal 1995:1099-104.
21. Lindholm LH, Ekbom T, Dash C, et al. The impact of health care advice given in primary care on cardiovascular risk. BMJ 1995;**310**(6987):1105-09.

For peer review only

22. Meland E, Lærum E, Ulvik RJ. Effectiveness of two preventive interventions for coronary heart disease in primary care. Scandinavian journal of primary health care 1997;**15**(1):57-63.

23. Avram C, Iurciuc M, Craciun L, et al. Dietary and physical activity counseling in high-risk asymptomatic patients with metabolic syndrome–A primary care intervention. Journal of Food, Agriculture & Environment 2011;**9**(3&4):16-19.

24. Steptoe A, Day S, Doherty S, et al. Behavioural counselling in general practice for the promotion of healthy behaviour among adults at increased risk of coronary heart disease: randomised trialCommentary: Treatment allocation by the method of minimisation. Bmj 1999;**319**(7215):943-48.

25. Sartorelli DS, Sciarra EC, Franco LJ, et al. Beneficial effects of short-term nutritional counselling at the primary health-care level among Brazilian adults. Public health nutrition 2005;**8**(07):820-25.

26. Ma J, Berra K, Haskell WL, et al. Case management to reduce risk of cardiovascular disease in a county health care system. Archives of internal medicine 2009;**169**(21):1988-95.

27. Tibblin G, Åberg H. NON-PHARMACOLOGICAL TREATMENT OF HYPERTENSION IN TWO STEPS-1 YEAR REPORT FROM EIGHT HEALTH CENTRES. Acta Medica Scandinavica 1986;**220**(S714):105-12.

For peer review only

Supplementary table 2: Intervention components and behaviour change techniques employed.

Study (Year)	Study groups	Who delivered it	BCTs [1]	Mode of delivery	No. of sessions	Duration of sessions (in mins)
**Kranjčević, et al.** [1]	Intervention	GPs	1.3, 2.1, 9.1	Face to face and written materials	5	Unclear
	Control		Unclear	Unclear	Unclear	Unclear
**Vetter, et al.** [2]	Intervention 1	PCP and lifestyle coach.	1.1, 1.5, 2.3, 8.7, 9.1	Face to face and written materials	32	Visits: 5-7, counselling: 10-15.
	Intervention 2		1.1, 1.5, 2.3, 8.7, 9.1, 11.1	Face to face and written materials	32	Visits: 5-7, counselling: 10-15.
	Control		1.7	Face to face	8	Visits: 5-7.
**Lakerveld, et al.** [3]	Intervention		1.2, 1.6	Face to face and phone sessions.	9	Face to face sessions: 30.
	Control	Nurse	4.1, 5.1	Written materials.	Unclear	Unclear
**Hardcastle, et al.** [4]	Intervention	PA specialist and dietician	1.1, 1.5, 9.2	Face to face.	5	20-30.
	Control		5.1	Written materials.	Unclear	Unclear
**Tiessen, et al.** [5]	Intervention	Practice nurses.	2.2, 2.3, 2.4, 5.1	Face to face.	7	First session: 20 min, other sessions based on patient preference.
	Control		5.1	Face to face and written materials.	One	Unclear.
**Parra-Medina, et al.** [6]	Intervention	PCP, health educators and nurses	1.1, 1.2.	Face to face and telephone sessions and written materials.	Up to 15	First session: 60. Following sessions: 20.
	Control		1.1	Face to face and written materials.	One	5-10 mins.
**Drevenhorn, et al.** [7]	Intervention	Nurses	1.1, 1.5, 5.3, 9.2, 10.4, 11.2	Face to face	Unclear	Unclear
	Control		Unclear	Unclear	Unclear	Unclear
**Brett, et al.** [8]	Intervention		1.1, 1.3, 2.7	Face to face	5	Unclear
	Control	GPs	1.1, 1.3, 2.7	Face to face	2	Unclear
**Harris, et al.** [9]	Intervention	Health practitioner, dietitian or PT	1.1, 1.2, 2.3, 4.1, 6.1, 9.1	Face to face	6	90 mins/ session.
	Control		Unclear	Unclear	Unclear	Unclear
**Mendis, et al.** [10]	Intervention	Health-care workers	2.6, 4.1	Face to face and written materials	4	Unclear
	Control		Unclear	Unclear	Unclear	Unclear
**Koelewijn-van Loon, et al.** [11]	Intervention	Nurses	1.1, 1.2, 1.4, 1.5, 5.1, 9.2	Face to face and telephone sessions	3	Face to face: 10-20, telephone: 10.
	Control		5.1	Face to face	One	Unclear
**Eriksson, et al.** [12]	Intervention	Dietician, PT and assistants.	1.1, 1.2, 1.3, 1.4, 4.1, 5.1, 8.1, 8.7, 9.1, 9.2	Face to face	56	Unclear.
	Control		9.1	Face to face and written materials.	One	Unclear

For peer review only

**Phelan, et al.** [13]	Intervention 1	PCP	2.3, 11.1.	Face to face and written materials.	8	5-10.
	Intervention 2	Psychologist	1.5, 2.3, 9.1	Group sessions.	29	90
	Intervention 3	Psychologist, PCP	1.5, 2.3, 9.1, 11.1	Face to face, group sessions and written material.	37	Face to face: 5-10, group sessions: 90.
	Intervention 4	PCP	1.5, 2.3, 9.1, 11.1	Face to face and written materials.	8	5-10.
**Harting, et al.** [14]	Intervention	Practice assistant and dietician.	1.1, 1.4, 9.1, 11.1	Face to face, telephone sessions and written materials.	Unclear	Unclear
	Control		Unclear	Unclear	Unclear	Unclear
**Korhonen, et al.** [15]	Intervention	Healthcare centre personnel.	1.1, 1.3, 1.4, 2.3, 2.5,4.1, 9.1	Face to face.	7	Unclear
	Control		Unclear	Unclear	Unclear	Unclear
**Baron, et al.** [16]	Intervention	Nurse	5.1, 9.1	Face to face, group sessions and written material.	Unclear	30.
	Control		Unclear	Unclear	Unclear	Unclear
**Knutsen and Knutsen** [17]	Intervention	Physicians and dieticians	1.1, 4.1, 5.1, 6.1, 9.1	Face to face and telephone sessions.	8	Unclear
	Control		Unclear	Unclear	Unclear	Unclear
**Nilsson, et al.** [18]	Intervention	Nurse, dietician or PT.	1.1, 2.2, 3.1, 4.1, 6.1, 9.1, 12.5	Face to face, group sessions and videotapes.	Unclear	Unclear
	Control		2.2, 5.1	Face to face	One	Unclear
**Wood, et al.** [19]	Intervention	Nurses	1.1, 2.7, 5.1, 6.2, 9.1	Face to face and written materials	Unclear	First session: 90.
	Control		9.1	Face to face	One	45 mins
**OXCHECK Study Group** [20]	Intervention	Nurses	1.3, 2.7, 9.1,	Face to face	Unclear	Initial session: 45-60, following sessions: 10-20.
	Control		Unclear	None	None	None
**Lindholm, et al.** [21]	Intervention	Doctors and nurses	2.3, 4.1, 5.1, 6.2, 9.1	Face to face, group sessions and written materials	11	Five group sessions: 90, one group session: all day.
	Control		9.1	Face to face and written materials	5	Unclear
**Meland, et al.** [22]	Intervention	GPs	1.8, 2.3, 8.7, 9.1, 11.2	Face to face and written materials	4	Unclear
	Control		9.1	Face to face and written materials	4	Unclear
**Avram, et al.** [23]	Intervention	GPs	1.1, 9.1	Face to face and telephone sessions	21	Face to face sessions: 30.
	Control		Unclear	Written materials	None	None
**Steptoe, et al.** [24]	Intervention	Nurses	1.1, 1.4, 9.1, 11.1	Face to face and telephone sessions	2-3	Face to face sessions: 20.
	Control		Unclear	Unclear	Unclear	Unclear
**Sartorelli, et al.**	Intervention	Nutritionist	1.1, 1.4, 9.1	Face to face and group sessions and	4	Unclear

For peer review only

[25]				written materials.		
	Control		Unclear	Group session and written materials	1	Unclear
**Ma, et al.** [26]	Intervention	Nurses and dietitians	1.1, 1.2, 1.7, 9.1, 11.1, 11.2	Face to face	8-10	30-60
	Control		Unclear	Unclear	Unclear	Unclear
**Tibblin and Åberg** [27]	Intervention	Nurses and physicians	2.5, 6.1, 9.1	Face to face, group sessions and videotapes and audiotapes.	15	Unclear
	Control		2.5, 9.1	Face to face	15	Unclear

[1] as coded in Michie, Richardson et al.[28] taxonomy of behaviour change technique

Note: 1.1 Goal setting (behaviour); 1.2 Problem solving; 1.3 Goal setting (outcome); 1.4 Action planning; 1.5 Review behaviour goals(s); 1.6 Discrepancy between current behaviour and goal; 2.1 Monitoring of behaviour by others without feedback; 2.2 Feedback on behaviour; 2.3 self-monitoring of behaviour; 2.4 Self-monitoring of outcome(s) of behaviour; 2.5 Monitoring of outcomes of behaviour without feedback; 2.6 Biofeedback; 2.7 Feedback on outcome(s) of behaviour; 3.1 Social support (unspecified); 4.1 Instructions on how to perform a behaviour; 5.1 Information about health consequences; 5.3 Information about social and environmental consequences; 6.1 Demonstration of the behaviour; 6.2 Social comparison; 8.1 Behavioural practice/rehearsal; 8.7 Graded tasks; 9.1 Credible source; 9.2 Pros and cons; 10.4 Social reward; 11.1 Pharmacological support; 11.2 Reduce negative emotions; 12.5 Adding objects to the environment; PT Physiotherapist, PA Physical activity

For peer review only

References:

1. Kranjčević K, Marković BB, Lalić DI, et al. Is a targeted and planned GP intervention effective in cardiovascular disease prevention? A randomized controlled trial. Medical science monitor: international medical journal of experimental and clinical research 2014;**20**:1180.
2. Vetter ML, Wadden TA, Chittams J, et al. Effect of lifestyle intervention on cardiometabolic risk factors: results of the POWER-UP trial. International Journal of Obesity 2013;**37**:S19-S24.
3. Lakerveld J, Bot SD, Chinapaw MJ, et al. Motivational interviewing and problem solving treatment to reduce type 2 diabetes and cardiovascular disease risk in real life: a randomized controlled trial. Int J Behav Nutr Phys Act 2013;**10**(47):10.1186.
4. Hardcastle SJ, Taylor AH, Bailey MP, et al. Effectiveness of a motivational interviewing intervention on weight loss, physical activity and cardiovascular disease risk factors: a randomised controlled trial with a 12-month post-intervention follow-up. Int J Behav Nutr Phys Act 2013;**10**(40):1-16.
5. Tiessen AH, Smit AJ, Broer J, et al. Randomized controlled trial on cardiovascular risk management by practice nurses supported by self-monitoring in primary care. BMC family practice 2012;**13**(1):1.
6. Parra-Medina D, Wilcox S, Salinas J, et al. Results of the Heart Healthy and Ethnically Relevant Lifestyle trial: a cardiovascular risk reduction intervention for African American women attending community health centers. American journal of public health 2011;**101**(10):1914-21.
7. Drevenhorn E, Bengtson A, Nilsson PM, et al. Consultation training of nurses for cardiovascular prevention–a randomized study of 2 years duration. Blood pressure 2012;**21**(5):293-99.
8. Brett T, Arnold-Reed D, Phan C, et al. The Fremantle Primary Prevention Study: a multicentre randomised trial of absolute cardiovascular risk reduction. Br J Gen Pract 2012;**62**(594):e22-e28.
9. Harris MF, Fanaian M, Jayasinghe UW, et al. A cluster randomised controlled trial of vascular risk factor management in general practice. Med J Aust 2012;**197**(7):387-93.
10. Mendis S, Johnston SC, Fan W, et al. Cardiovascular risk management and its impact on hypertension control in primary care in low-resource settings: a cluster-randomized trial. Bulletin of the World Health Organization 2010;**88**(6):412-19.
11. Koelewijn-van Loon MS, van der Weijden T, van Steenkiste B, et al. Involving patients in cardiovascular risk management with nurse-led clinics: a cluster randomized controlled trial. Canadian Medical Association Journal 2009;**181**(12):E267-E74.
12. Eriksson MK, Franks PW, Eliasson M. A 3-year randomized trial of lifestyle intervention for cardiovascular risk reduction in the primary care setting: the Swedish Björknäs study. PloS one 2009;**4**(4):e5195.
13. Phelan S, Wadden T, Berkowitz R, et al. Impact of weight loss on the metabolic syndrome. International journal of obesity 2007;**31**(9):1442-48.
14. Harting J, van Assema P, van Limpt P, et al. Cardiovascular prevention in the Hartslag Limburg project: effects of a high-risk approach on behavioral risk factors in a general practice population. Preventive medicine 2006;**43**(5):372-78.
15. Korhonen M, Kastarinen M, Uusitupa M, et al. The effect of intensified diet counseling on the diet of hypertensive subjects in primary health care: a 2-year open randomized controlled trial of lifestyle intervention against hypertension in eastern Finland. Preventive medicine 2003;**36**(1):8-16.
16. Baron JA, Gleason R, Crowe B, et al. Preliminary trial of the effect of general practice based nutritional advice. Br J Gen Pract 1990;**40**(333):137-41.

For peer review only

17. Knutsen SF, Knutsen R. The Tromsø Survey: the Family Intervention study—the effect of intervention on some coronary risk factors and dietary habits, a 6-year follow-up. Preventive medicine 1991;**20**(2):197-212.
18. Nilsson PM, Lindholm LH, Scherstén BF. Life style changes improve insulin resistance in hyperinsulinaemic subjects: a one-year intervention study of hypertensives and normotensives in Dalby. Journal of hypertension 1992;**10**(9):1071-78.
19. Wood D, Kinmonth A, Davies G, et al. Randomised controlled trial evaluating cardiovascular screening and intervention in general practice: principal results of British family heart study. Bmj 1994;**308**(6924):313-20.
20. OXCHECK. Effectiveness of health checks conducted by nurses in primary care: final results of the OXCHECK study. BMJ: British Medical Journal 1995:1099-104.
21. Lindholm LH, Ekbom T, Dash C, et al. The impact of health care advice given in primary care on cardiovascular risk. BMJ 1995;**310**(6987):1105-09.
22. Meland E, Lærum E, Ulvik RJ. Effectiveness of two preventive interventions for coronary heart disease in primary care. Scandinavian journal of primary health care 1997;**15**(1):57-63.
23. Avram C, Iurciuc M, Craciun L, et al. Dietary and physical activity counseling in high-risk asymptomatic patients with metabolic syndrome–A primary care intervention. Journal of Food, Agriculture & Environment 2011;**9**(3&4):16-19.
24. Steptoe A, Day S, Doherty S, et al. Behavioural counselling in general practice for the promotion of healthy behaviour among adults at increased risk of coronary heart disease: randomised trialCommentary: Treatment allocation by the method of minimisation. Bmj 1999;**319**(7215):943-48.
25. Sartorelli DS, Sciarra EC, Franco LJ, et al. Beneficial effects of short-term nutritional counselling at the primary health-care level among Brazilian adults. Public health nutrition 2005;**8**(07):820-25.
26. Ma J, Berra K, Haskell WL, et al. Case management to reduce risk of cardiovascular disease in a county health care system. Archives of internal medicine 2009;**169**(21):1988-95.
27. Tibblin G, Åberg H. NON-PHARMACOLOGICAL TREATMENT OF HYPERTENSION IN TWO STEPS-1 YEAR REPORT FROM EIGHT HEALTH CENTRES. Acta Medica Scandinavica 1986;**220**(S714):105-12.
28. Michie S, Richardson M, Johnston M, et al. The behavior change technique taxonomy (v1) of 93 hierarchically clustered techniques: building an international consensus for the reporting of behavior change interventions. Annals of behavioral medicine 2013;**46**(1):81-95.

For peer review only

Supplementary table 3: Risk of bias assessment.

Study (Year)	Risk of bias					
	Sequence generation (randomisation methods) [a]	Allocation concealment [b]	Blinding of participants to study group allocation [c]	Blinding of trial personnel or outcome assessors [d]	Incomplete outcome data [e]	Selective reporting [f]
Kranjčević, K. et al (2014)	Unclear	Unclear	Unclear	Unclear	High	Low
Vetter et al. (2013)	Low	Low	High	Low	High	Low
Lakerveld et al. (2012)	Low	Low	High	High	High	Low
Hardcastle et al. (2013)	Low	Low	Low	Low	High	Low
Tiessen et al. (2012)	Low	Low	High	High	Unclear	Low
Parra-Medina et al. (2011)	Unclear	Unclear	Low	Low	High	High
Drevenhorn et al. (2012)	Unclear	Unclear	Unclear	Unclear	High	Low
Brett et al. (2012)	Low	High	High	High	Low	High
Harris et al. (2012)	Low	Low	High	Low	Low	Low
Mendis et al. (2010)	Unclear	Unclear	High	High	Low	Unclear
Koelewijn-van Loon et al. (2009)	Low	Low	High	High	High	Low
Eriksson et al. (2009)	Low	Low	High	High	Unclear	Low
Phelan et al. (2007)	Unclear	Unclear	Unclear	Unclear	Unclear	Unclear
Harting et al. (2006)	Unclear	Unclear	Unclear	Unclear	Unclear	Unclear
Korhonen et al. (2003)	High	High	High	Unclear	High	Low
Baron et al. (1990)	Unclear	Unclear	Unclear	Unclear	Unclear	Unclear
Knutsen and Knutsen (1991)	Low	Low	Unclear	Low	Low	Low
Nilsson et al. (1992)	Unclear	Unclear	Unclear	Low	Low	Low
Wood et al. (1994)	Unclear	Unclear	Unclear	Unclear	Unclear	Low
OXCHECK Study group (1995)	Unclear	Unclear	High	Low	Unclear	Low
Lindholm et al. (1995)	Unclear	Unclear	High	Unclear	Low	Low
Meland et al. (1997)	Unclear	Unclear	Unclear	Unclear	Unclear	Unclear
Avram et al. (2011)	Unclear	Unclear	Low	Low	Low	Unclear

Steptoe et al. (1999)	Low	Low	High	High	High	Low
Sartorelli et al. (2005)	Low	Low	Low	High	High	Unclear
Ma et al. (2009)	Low	Low	Low	Low	Unclear	Low
Åberg and Tibblin (1989)	Low	Low	Unclear	Unclear	Low	Unclear

[a] Assessment of whether or not methods used to generate the allocation sequence should produce comparable groups.
[b] Assessment of whether or not the method used to conceal allocation sequence is sufficient or not.
[c] Assessment of the methods used to blind study participants and personnel from knowing intervention allocation.
[d] Assessment of the methods used to blind study outcome assessors from knowing intervention allocation, and whether or not this method of blinding is sufficient.
[e] Assessment of whether incomplete outcome data were adequately dealt with. Studies missing outcome data for >20% of participants who underwent randomization were considered at high risk of bias, while studies missing <10% of participants who underwent randomization were considered at low risk of bias.
[f] Assessment of whether all outcome measures described in the introduction and methods section of the paper (and published protocols) were reported.

For peer review only

Supplementary table 4: Theory use evaluation using Theory Coding Scheme.

Study	Vetter et al. (2013)	Lakerveld et al. (2012)	Tiessen et al. (2012)	Parra-Medina et al. (2011)	Drevenhorn et al. (2012)	Harris et al. (2012)	Eriksson et al. (2009)	Steptoe et al. (1999)	Ma et al. (2009)
Theoretical base	Social cognitive and behavioural self-management theory	Theory of planned behaviour and theory of self-regulation.	Stages of change	Trans-theoretical model and social cognitive theory	Stages of changes model	Stages of change model	Stages of change model	Stages of change model	Social cognitive theory and trans-theoretical model
1) Theory/ model of behaviour mentioned	Yes	Yes	Yes	Yes	Yes	Yes	Yes	Yes	No
2) Targeted construct mentioned	Yes	Yes	No	Yes	Yes	Yes	Yes	Yes	No
3) Intervention based on single theory	No	No	Yes	No	Yes	Yes	Yes	Yes	No
4) theory used to select recipients	No	No	Yes	No	No	No	No	No	No
5) Theory used to select intervention techniques	Yes	Yes	Do not know	Yes	Yes	No	No	No	No
6) theory used to tailor intervention techniques to recipients	No	No	Do not know	Yes	Yes	Yes	No	No	No
7) <u>All</u> intervention techniques are explicitly linked to theory construct	No	Yes	No	Yes	Yes	No	No	No	No
8) <u>at least one</u> of the intervention techniques are explicitly linked to theory construct	Yes	No	No	No	No	No	No	No	No
9) Group of techniques are linked to a group of constructs	Yes	Yes	No	Yes	Yes	No	No	No	No
10) <u>All</u> theory relevant constructs are explicitly linked to at least one intervention technique.	No	No	No	No	Yes	No	No	No	No
11) <u>At least one</u> of the theory relevant constructs are explicitly linked to at least one intervention technique.	Yes	Yes	No	Yes	No	No	No	No	No
12) theory-relevant constructs are	No	B	No	No	No	No	No	Yes	No

For peer review only

measured									
13) Quality of measures	N/A	A	N/A	No	N/A	N/A	N/A	C and F	N/A
14) Randomization of participants condition	A, B, C and D	A and B	A,B,C and D	A	A and C	A and B	No	N/A	N/A
15) Changes in measured theory-relevant constructs	No	Yes	Do not know	Do not know	No	No	No	N/A	N/A
16) Mediational analysis of constructs	No	No	No	No	No	No	No	N/A	N/A
17) results discussed in relation to theory	No	Yes	No	No	No	No	No	N/A	No
18) Appropriate support for theory	No	No	Do not know	Do not know	No	No	No	N/A	No
19) Results used to refine theory	No	No	No	No	No	No	No	No	No

For peer review only

BMJ Open

Multiple health behaviour change interventions for primary prevention of cardiovascular disease in primary care: systematic review and meta-analysis

Journal:	*BMJ Open*
Manuscript ID	bmjopen-2016-015375.R1
Article Type:	Research
Date Submitted by the Author:	14-Mar-2017
Complete List of Authors:	Alageel, Samah; King's College London, Primary Care and Public Health<br>Gulliford, Martin; King's College London, UK<br>McDermott, Lisa; King's College London, Primary Care and Public Health Sciences<br>Wright, Alison; King's College London, Primary care and public health
**Primary Subject Heading**:	Public health
Secondary Subject Heading:	Cardiovascular medicine
Keywords:	cardiovascular diseases, health behaviour, PRIMARY CARE, primary prevention, meta-analysis

SCHOLARONE™ Manuscripts

For peer review only

Multiple health behaviour change interventions for primary prevention of cardiovascular disease in primary care: systematic review and meta-analysis

Samah Alageel MPH, Martin C Gulliford FFPH, Lisa McDermott PhD, Alison J. Wright PhD

Department of Primary Care and Public Health Sciences, King's College London

Correspondence: Samah Alageel
Department of Primary Care and Public Health Sciences,
Faculty of Life Sciences and Medicine.
King's College London, Addison House, Guy's Campus,
London SE1 1UL, United Kingdom
Email: samah.alageel@kcl.ac.uk
Tel: 0207 848 6631
Fax: 0207 848 6620

Word count:

Abstract	260
Text	4927
Tables	4
Figures	1

For peer review only

ABSTRACT

Background: It is uncertain whether multiple health behaviour change interventions (MHBC) are effective for the primary prevention of cardiovascular disease (CVD) in primary care. A systematic review and meta-analysis were performed to evaluate the effectiveness of MHBC interventions on CVD-risk and CVD risk factors; the study also evaluated associations of theoretical frameworks and intervention components with intervention effectiveness.

Methods: The search included randomised controlled trials of MHBC interventions aimed at reducing CVD-risk in primary prevention population up to 2017. Theoretical frameworks and intervention components were evaluated using standardised methods. Meta-analysis with stratification and meta-regression were used to evaluate intervention effects.

Results: We identified 31 trials (36,484 participants) with a minimum duration of 12 months follow-up. Pooled net change in systolic blood pressure (16 trials) was -1.86 (95% confidence interval -3.17 to -0.55, P= 0.01) mm Hg, diastolic blood pressure (15 trials) -1.53 (-2.43 to -0.62, P= 0.001) mm Hg, body mass index (14 trials) -0.13 (-0.26 to -0.01, P= 0.04) Kg/m^2 and serum total cholesterol (14 trials) -0.13 (-0.19 to -0.07, P <0.001) mmol/L. There was no significant association between interventions with a reported theoretical basis and improved intervention outcomes. No association was observed between intervention intensity (number of sessions and intervention duration) and intervention outcomes. There was significant heterogeneity for some risk factor analyses, leading to uncertain validity of some pooled net changes.

Conclusions: MHBC interventions delivered to CVD-free participants in primary care did not appear to have quantitatively important effects on CVD risk factors. Better reporting of interventions' rationale, content and delivery is essential to understanding their effectiveness.

For peer review only

Key words: Cardiovascular Diseases, Health Behaviour, Primary Health Care, Meta-analysis, Primary Prevention, Risk Factors.

For peer review only

Strengths and limitations:

- The review presents evidence of head to head meta-analysis of 31 published randomised controlled trials of MHBC interventions and cardiovascular risk with follow-up for 12 months or longer.
- The study employed standardised instruments to evaluate the impact of theory use and behaviour change techniques in MHBC interventions.
- The majority of trials included were conducted in Europe and United States and only English language publications were included.
- Not all studies evaluated all outcomes of interest and some lacked detail concerning intervention design and delivery.

For peer review only

INTRODUCTION

Cardiovascular disease (CVD) is the leading cause of death worldwide, accounting for over 30% of global mortality [1]. CVD is mediated by several antecedent behavioural risk factors, and its onset might be prevented or delayed by altering one or several risk factors [1]. Risk factors for CVD are inter-related and often coexist [2-4]. This observation has informed the development of multiple health behaviour change (MHBC) interventions for reduction of CVD-risk. Identifying individuals at high-risk of CVD in primary care, and encouraging lifestyle change to reduce risk factors, represents a widely used strategy for the primary prevention of cardiovascular diseases. Randomised controlled trials have been conducted in primary care to evaluate the effectiveness of MHBC interventions using lifestyle modification techniques instead of, or in addition to, pharmacological treatment to modify CVD risk factors. These trials have generally provided only equivocal evidence for reduction of CVD incidence through MHBC but the degree of effectiveness might be associated with level of risk [5-7]. Results from Ebrahim et al.'s [5] systematic review suggested that MHBC interventions have negligible effect on mortality in unselected populations, with a pooled odds ratio for coronary heart disease mortality of 0.99 (95% CI 0.92 to 1.07). Evidence of benefit was found in studies in high-risk populations including people with hypertension (OR 0.78, 0.68 to 0.89) or diabetes (OR 0.71, 0.61 to 0.83) [5]. However, general health checks were not found to reduce all cause-mortality, nor CVD- or cancer-related morbidity and mortality [8].

Previous reviews have assessed the effectiveness of MHBC interventions in reducing CVD morbidity and mortality [5 6 8], less is known about the effectiveness of these interventions in reducing CVD-risk and risk factor values in primary care.

For peer review only

In recent years, there has been growing appreciation of the role of employing psychological theory in behaviour change intervention design, and studying the impact of specific behaviour change techniques (BCT) on intervention outcomes [9]. Theories of the psychological determinants of behaviour can be used to inform the development and evaluation of behaviour change interventions [10]. Interventions are likely to be more effective when they systematically target psychological determinants of behaviour[11]. A review of internet-based interventions suggested that more intensive use of theory was associated with greater behaviour change [12], but another review found little evidence of an association between theory use and intervention effects on healthy eating or physical activity [13]. This equivocal evidence could arise if a high proportion of behaviour change interventions are not based on a theory or the theory is not applied extensively [14].

Behaviour change techniques (BCT) are 'the active components of an intervention designed to change behaviour' [15]. Identifying specific BCTs associated with greater impact on intervention effectiveness is essential for future intervention design [16]. Previous reviews suggested that interventions using the BCTs "provision of instructions," "self-monitoring of behaviour," "relapse prevention," and "prompt practice" led to greater reductions in weight among obese individuals [17], while interventions designed to modify physical activity and/or diet were more effective when they included self-monitoring plus one of the four following behaviour change techniques: prompting intention formation, specific goal setting, review of behavioural goals or providing feedback on performance[18]. Identifying BCTs associated with greater intervention effectiveness and exploring the impact of applying theory will contribute to the design of future MHBC interventions targeting CVD risk in primary care.

For peer review only

Objectives

This systematic review had three objectives: first, to assess the effectiveness of MHBC interventions, directed at changing two or more behaviours, at reducing CVD-risk and CVD risk factors in adults without existing cardiovascular conditions; secondly, to evaluate whether using theory to develop interventions is associated with intervention effectiveness; and thirdly, to evaluate the association between behaviour change techniques employed and intervention effects.

For peer review only

METHODS

Studies were selected according to the following criteria:

Participants

Trials that recruited an adult population (>18 years old) free of CVD were included. Following previous reviews [5], we included trials with less than 20% participants with CVD. Studies of patient populations with established disease, such as diabetes, were excluded.

Interventions

We included studies that evaluated behaviour change interventions aimed at reducing CVD-risk by intervening on two or more risk behaviours at the same time. Risk behaviours included: physical activity, diet, alcohol consumption, use of stress management and smoking. Comparators were usual care or less intensive interventions.

Settings

Interventions where participants were recruited, and interventions were delivered by trained healthcare professionals or primary care staff, in primary care premises (including general practice, family practice or primary care clinic).

Study design

Controlled trials, with individual or cluster randomisation, providing ≥12 month follow-up for outcome evaluation.

For peer review only

Outcome measures

Long term outcomes of MHBC interventions including CVD mortality and clinical events have been reported previously [5 6] and only one study in 2015 included clinical events as an outcome. Therefore long term outcomes were not included in this systematic review. Primary outcomes were changes in CVD-risk scores, body mass index (BMI) or body weight, blood pressure, and serum total cholesterol levels. We have excluded diabetes management trials, therefore, diabetes control outcomes were not included. Secondary outcomes were changes in physical activity, diet, smoking and alcohol consumption.

Language

Studies reported in English.

Search strategy

Multiple sources of ascertainment were used, including electronic databases (Medline, EMBASE, PsycINFO and CENTRAL) and searching reference lists of included papers. The search results and search terms of previous review [5] were used with searching extended from 2006 until February 2017. Search terms used included primary prevention, multiple risk factor, lifestyle intervention, health education and health promotion. Appendix A presents the search strategies used. Titles were screened by one reviewer (SA) and a second reviewer (MG) checked a random set of studies, approximately 10% of the search results, to assess agreement regarding whether they met the inclusion criteria. Disagreements were resolved through discussion, until full agreement was reached. The selection process is displayed in Figure 1.

For peer review only

Methodologic quality

Studies were evaluated using the Cochrane risk of bias tool [19]. This assesses six domains of bias including selection bias, performance bias, detection bias, attrition bias, reporting bias and other biases [19].

Data extraction

Interventions were coded by country, target behaviours, participant and intervention characteristics, mode of delivery and intervention outcomes. We attempted to contact study authors to provide additional information where necessary However, when information was not available, we assumed missing outcome data to occur at random.

In addition, Michie and Prestwich's [20] method of assessing the application of theory in the development and evaluation of behaviour change interventions was used. The Theory Coding Scheme (TCS) consists of 19 items that cover different aspects that may be informed by theory [20]. We used three measures to capture the extent of theory use, as employed in a previous review[13]: The first concerned whether the intervention was explicitly based on a theory or combination of theories or predictors (TCS item 5). Secondly, we assessed the degree to which each BCT reported as part of the intervention was linked to a theory-relevant construct (scored +2 for the ideal scenario of "yes" to TCS item 7 (all intervention techniques explicitly linked to at least one theory-relevant construct), +1 for studies coded "yes" for TCS item 8 (at least one, but not all, intervention techniques explicitly linked to at least one theory-relevant construct) and/or TCS item 9 (group of BCTs are linked to a group of constructs) and 0 for studies coded "no" for all of items 7-9. Finally, we rated the extent to which all constructs in the relevant theory had been explicitly targeted by BCTs. This was scored +2 for the ideal scenario of "yes" to TCS item 10 (all theory-relevant constructs explicitly linked to at least one BCT), +1 for "yes" to TCS item 9 (group of BCTs are linked to a group of constructs) and/or item 11 (at least one, but not all, theory relevant

For peer review only

constructs are explicitly linked to at least one BCT) and 0 for interventions coded “no” to all of items 9-11.

The theory-based taxonomy of 93 behaviour change techniques developed by Michie, Richardson et al.[9] was used to identify intervention techniques. The assessment was completed by two researchers (LM and SA) with good agreement for intervention groups (77.8% agreement) and control groups (92.6% agreement). Discrepancies were discussed and resolved to reach full agreement. Intervention characteristics and BCTs were also extracted from descriptions of the control group, because the chosen nature of the control group can influence the apparent effectiveness of interventions [21]. Where detail of interventions was lacking, we attempted to contact study authors to provide additional information.

Data analysis

Outcome data were combined in random effects meta-analyses using ‘metan’ commands in STATA. DerSimonian and Laird [22] random effect models were chosen due to the considerable heterogeneity for certain outcomes. For continuous outcomes we used mean changes in each trial arm to calculate net effects. We expressed effects for binary variables as risk differences. We quantified statistical heterogeneity using I^2 statistic. We have examined the influence of individual studies in outcomes with considerable heterogeneity (I^2>50%) by omitting one study at a time to see the extent to which heterogeneity could be explained by a study or group on studies (leave-one-out analysis).

Meta-regression analyses were used to examine the effect of medication use, number of interventions’ sessions, intervention duration, types of BCTs used and theory use on intervention outcomes. Intervention duration was calculated by multiplying the number of sessions and the sessions’ duration. Publication bias was assessed using Egger’s

For peer review only

regression test [23] using ‘metabias’ and ‘metafunnel’ commands in STATA. If bias existed, the “trim and fill”[24] method was used to adjust for publication bias. Mendis et al [25] Nigeria site’s study had unusually high summary estimates, and heterogeneity diminished substantially after this study was excluded. This study was therefore treated as an outlier and results were reported with the exclusion of this study.

For peer review only

RESULTS

The initial search identified 26656 references, with 55 relevant trials identified from the previous systematic review [5]. After removing duplicates, 21089 titles were screened. A total of 31 trials were included in this review (Figure 1).

Included studies

We identified a total of 31 trials of MHBC intervention for the primary prevention of CVD in primary care with 36484 participants. The duration of follow-up ranged from 12 months to 6 years (median 12 months). Intervention duration ranged from two months up to three years (median 12 months). Summary of included studies characteristics are presented in table 1 and supplementary table 1.

For peer review only

Table 1: Summary of characteristics of 27 trials included in the review. Figures are frequencies (column percent).

Characteristics		Freq. (%)
**Total**		31 (100)
**Country**	UK	6 (19.4%)
	Sweden	5 (16.1%)
	Netherlands	4 (12.9%)
	USA	4 (12.9%)
	Europe	7 (22.6%)
	Others	5 (16.1%)
**Number of participants**	Median (IQR)	419 (224-883)
**Gender**	Male only	1 (3.2)
	Female only	1 (3.2)
	Both	29 (93.5)
**Age**	Minimum age, median (IQR)	30 (20-40)
	Maximum age, median (IQR)	65 (60-74)
**Intervention outcomes**	CVD risk	14 (45.2)
	Body weight	25 (80.6)
	Blood pressure	26 (83.9)
	Serum cholesterol	26 (83.9)
	Diet	18 (58.1)
	Physical activity	21 (67.7)
	Alcohol	6 (19.4)
	Smoking	15 (48.4)
**Number of targeted behaviours**	2 behaviours	11 (35.5)
	3 behaviours	12 (38.7)
	4 behaviours	7 (22.6)
	5 behaviours	1 (3.2)
**Follow-up duration**	12 months	18 (58.1)
	>12 months	13 (41.9)

CVD, cardiovascular disease; IQR, interquartile range

For peer review only

Study characteristics

Diet and physical activity were targeted in 11 trials, with nine trials targeting diet, physical activity and smoking. Diet, physical activity, smoking and alcohol consumption were targeted in seven interventions and two interventions targeted diet, physical activity and stress management. Only one intervention targeted diet, physical activity, stress and alcohol consumption and one intervention targeted all five behaviours. A wide range of intervention modalities was investigated (Table 2 and supplementary table 2), including individual and group sessions, telephone conversations and provision of written materials. The majority of the included trials reported offering “usual care” to the control group, with few details provided. Seven trials offered face-to-face sessions and seven trials offered face-to-face sessions and written materials. Written materials alone were offered in three trials and no intervention was offered to the control group in three interventions.

For peer review only

Table 2: Summary of interventions characteristics for 27 trials included in the review. Figures are frequencies (column percents)

		Intervention N (%)	Control N (%)
**Type of staff delivering intervention**	GPs and physicians	10 (32.3)	
	Nurses	15 (48.4)	
	Dietitian	7 (22.6)	
	Others	12 (38.7)	
**Mode of intervention delivery**	Face to face sessions	30 (96.8)	14 (45.2)
	Group sessions	9 (29.0)	1 (3.2)
	Written materials	15 (48.4)	7 (22.6)
	Telephone sessions	8 (25.8)	-
	Unclear	-	13 (41.9)
**Number of intervention sessions**	1-4 sessions	5 (16.1)	9 (29.0)
	5-9 sessions	11 (35.5)	2 (6.5)
	10-15 sessions	4 (12.9)	1 (3.2)
	>15 sessions	5 (16.1)	1 (3.2)
	Unclear	6 (19.4)	18 (58.1)
**Number of behaviour change techniques (BCT)**	1-2 BCTs	5 (16.1)	14 (45.2)
	3-4 BCTs	10 (32.3)	1 (3.2)
	5-6 BCTs	12 (38.7)	-
	7-9 BCTs	3 (9.7)	-
	10 BCTs	1 (3.2)	-
	Unclear	-	16 (51.6)
**Frequently used behaviour change techniques**	Credible source (9.1)	22 (70.9)	6 (19.4)
	Goal setting (behaviour) (1.1)	19 (61.3)	2 (6.5)
	Information about health consequences (5.1)	9 (29.0)	5 (16.1)
	Instruction on how to perform a behaviour (4.1)	9 (29.0)	1 (3.2)
	Action planning (1.4)	9 (29.0)	-
	Self-monitoring of behaviour (2.3)	8 (25.8)	-

For peer review only

Risk of bias in included studies

Risk of bias assessment is presented in supplementary table 3. Half of the included trials (n=16) reported using intention-to-treat (ITT) analysis, while 15 studies did not state ITT procedures. Loss to follow-up ranged from 1.5% to 50.9%. Random allocation methods were not usually reported. In only 14 out 31 trials the method used was considered adequate. It is not possible to blind participants and personnel to treatment allocation in lifestyle intervention, which raises the possibility of bias inevitably. Only 5 trials have reported blinding of participants and personnel. Eleven trials have reported blinding outcomes assessors to treatment allocation, this too makes the assessment of outcomes likely biased (e.g. self-reported outcomes). Not all trials reported sufficient detail to assess risk of bias and these were rated as 'unclear'.

Treatment fidelity

Few studies reported using fidelity checks [26-30] to confirm that interventions were delivered as intended and this raises a question of whether the interventions were delivered as planned, and in a consistent manner.

Effect of interventions

Pooled effect sizes for all outcomes are presented in Table 3 and forest plots are presented in Appendix B.

For peer review only

Table 3: Pooled effects from meta-analysis of multiple health behaviour interventions on CVD-risk and CVD risk factors.

Outcome	N		Pooled effect size	95% confidence interval	P value	I^2 (%)	P value for heterogeneity	Tau^2
Systolic blood pressure (mmHg)	16		-1.86	-3.17 to -0.55	0.01	63.0	<0.001	3.91
Systolic blood pressure (mmHg) by medication use	10	Medication	-2.59	-4.48 to -0.69	0.01	68.3	0.001	5.31
	6	None [a]	-0.55	-1.69 to 0.59	0.35	3.4	0.40	0.09
Diastolic blood pressure (mmHg)	15		-1.53	-2.43 to -0.62	0.001	68.3	<0.001	1.92
Diastolic blood pressure (mmHg) by medication use	10	Medication [a]	-1.96	-2.79 to -1.11	<0.001	42.5	0.07	0.66
	5	None	-0.78	-2.50 to 0.93	0.37	73.0	<0.001	2.97
Serum total cholesterol (mmol/L)	14		-0.13	-0.19 to -0.07	<0.001	20.3	0.22	0.0
Serum total cholesterol (mmol/L) by medication use	8	Medication	-0.15	-0.26 to -0.03	0.01	43.8	0.09	0.01
	6	None [a]	-0.11	-0.18 to -0.03	0.01	0.0	0.60	0.0
Smoking (%)	11		-0.00	-0.02 to 0.01	0.66	13.4	0.31	0.0
Body mass index (Kg/m^2)	14		-0.13	-0.26 to -0.01	0.04	0.0	0.82	0.0
Body weight (Kg)	10		-0.91	-1.39 to -0.43	<0.001	12.1	0.33	0.08
CVD-risk using SCORE (%)	2		0.12	-0.37 to 0.61	0.61	0.0	0.87	0.0
Systolic blood pressure (mmHg) by theory use	5	Theory	-2.18	-5.92 to 1.56	0.25	72.3	0.01	13.0
	11	None [b]	-1.69	-3.01 to -0.29	0.02	61.3	<0.01	3.01
Diastolic blood pressure (mmHg) by theory use	5	Theory	-1.25	-2.43 to -0.06	0.04	0.4	0.40	0.01
	10	None [b]	-1.67	-2.83 to -0.52	<0.001	76.9	<0.001	2.42
Serum total cholesterol (mmol/L) by theory use	4	Theory	-0.03	-0.15 to 0.10	0.68	0.0	0.48	0.0
	10	None [b]	-0.13	-0.20 to -0.07	<0.001	0.0	0.29	0.0
Body mass index(Kg/m^2) by theory use	5	Theory	-0.15	-0.41 to 0.10	0.24	0.0	0.96	0.0
	9	None [b]	-0.13	-0.28 to 0.02	0.10	0.0	0.44	0.0
Body weight by (Kg) theory use	4	Theory	-0.24	-0.94 to 0.45	0.49	0.0	0.97	0.0
	8	None [b]	-1.32	-1.80 to -0.83	<0.001	0.0	0.53	0.0

N, number of trials; I^2, index of heterogeneity; a, medication use is not reported; b, theory use is not reported.

For peer review only

Changes in CVD risk factors

Blood pressure: Sixteen trials [25 27 31-44] reported changes in participants' systolic blood pressure (SBP) with no evidence of publication bias (Egger's test, P= 0.79). The weighted mean difference in SBP was -1.86 mm Hg (95% CI -3.17 to -0.55 mm Hg; P= 0.01). Diastolic blood pressure (DBP) was reported in 15 trials [25 27 31-33 35-44], with no evidence of publication bias (Egger's test, P= 0.19). Weighted mean difference in DBP was -1.53 mmHg (-2.43 to -0.62 mm Hg; P= 0.001). Out of the 12 interventions that evaluated blood pressure, seven reported that participants in all study groups were taking antihypertensive medications and three reported they were taking unspecified medications. There is no significant differences between the impact of trials that reported use of medication on SBP (β=-1.72, P= 0.23) and DBP (β= -1.46, P=0.12) compared to trials that did not report using medications.

Serum total cholesterol: Fourteen trials [27 31-33 35-37 39-45] evaluated serum total cholesterol and provided sufficient data for analysis (Egger's test, P= 0.55). Serum total cholesterol levels showed a small decrease in favour of intervention (-0.13 mmol/L; 95% -0.19 to -0.07; P<0.001). Six of the trials included in the analysis reported the use of lipid lowering medication and two reported the use of unspecified medication by all study groups. The weighted mean difference for total cholesterol was not different between trials that reported using medication and trials that have not stated using medications (β= 0.01, P=0.75) (Table 3).

Smoking: Eleven studies [25 26 29-32 34 37 44 46 47] reported smoking prevalence following the intervention. The pooled analysis showed no evidence of reductions in smoking behaviour (RD -0.00%; 95% CI -0.02 to 0.01; P=0.66). All studies included in the analysis relied on

For peer review only

self-reported smoking status and only two [29 44] reported using smoking cessation medication. There was no evidence of publication bias (P=0.47).

Weight and body mass index (BMI): Fourteen studies [25 27 31 33 35-44] reported on BMI as an outcome. The weighted mean change was -0.13 kg/m^2 (95% CI -0.26 to -0.01; P=0.04). The results of "trim and fill" method indicated that the weighted mean did not change despite the existence of publication bias (Egger's test P=0.002). Fewer studies (n=12) [27 33 35-37 40-44 47 48] reported on weight changes, showing a reduction of -0.91 kg (CI -1.39 to -0.43 kg; P< 0.001) with no evidence of publication bias (P=0.97).

Dietary behaviour: Sixteen trials [25-30 33 34 42-45 47-50] reported dietary behaviours as an outcome of the interventions. Outcomes of dietary interventions were measured using diverse methods, therefore, a meta-analysis was not conducted. Trials used a range of dietary self-report instruments to assess dietary behaviour, and none have used additional objective measures. Fruit and vegetable consumption was reported either as portions per day [25-27 43 47 50], or proportion of participants who met the recommendation for fruits and vegetable intake [26 33 34]. There was no positive effect of the intervention on fruits and vegetable consumption in most of the trials [26 27 34 47], and some trials did report improvement following the intervention [33 43 50], Fat intake was commonly measured as a dietary outcome either in terms of fat intake per day, [27 33 48 49] or as a fat score [29 34]. All the trials reported reductions in fat intake after the intervention, except Koelewijn-van Loon et al.[34] trial, where there was no significant difference between the intervention and control group.

Physical activity behaviour: Twenty trials reported changes in physical activity [26-34 36 37 39 40 42-44 46 47 50 51]. Physical activity was assessed via self-report. Due to the variety of measurements used, meta-analysis was not feasible. Some trials reported physical activity

For peer review only

as the proportion of participants who are physically active [29 31 39 46 50]. Other studies measured physical activity as the number of minutes per week, [27 34 42 43] or classified participants based on their weekly exercise [26 28 44 50]. Eight of these trials [27 29 30 36 37 40 42 44 47 50] resulted in an increase in reported physical activity following the intervention, and nine [26 28 31-33 39 43 46 48 51] trials concluded that the intervention had no impact on physical activity.

Alcohol consumption: Alcohol consumption was reported as an outcome in seven trials [30 34 40 46-48 50]. However, it was measured differently, which did not allow for pooled effect analysis. Two trials [40 46] reported reductions in alcohol consumption following the interventions, whereas the majority of the studies [30 34 47 48 50] did not find significant reductions in alcohol intake.

Cardiovascular disease risk: Studies used different risk scores to examine the effect of interventions on CVD-risk. Two studies [38 51] used the Framingham risk equation [52], two studies [30 53] used the Dundee risk score [54] and one study[44] used QRISK2 score[55]. These trials reported larger CVD-risk reductions in the intervention group compared to the control group. All of these trials had missing data making it not possible to analyse the pooled effect. Four studies [26 34 39 46] used the SCORE risk equation [56], however because of missing data we only included two studies [26 34] in the analysis, both conducted in the Netherlands. There was a non-significant increase in weighted mean difference of 0.12% CVD-risk (95% CI -0.37 to 0.61; P= 0.62).

Sensitivity analysis:

In outcomes of considerable heterogeneity (I^2>50%) we sought to identify possible causes by exploring the effect of included studies using leave-one-out sensitivity analysis. The absence of study Mendis et al. (China site) [25] and Koelewijn-van Loon, et al. [34] in analysing

For peer review only

the impact of interventions of systolic blood pressure reduces heterogeneity from I^2= 63% to I^2= 49.4% and generated a weighted mean difference (-1.86; CI -3.17 to -0.54; P=0.001) similar to the one obtained with all 16 trials. For diastolic blood pressure, removing Knutsen and Knutsen [31] from the analysis have resulted in reducing heterogeneity from I^2= 68.3% to I^2= 37.8% and produced a larger weighted mean difference (-1.93; CI -2.69 to -1.18; P<0.001).

Intervention components:

Intervention time and number of sessions: The number of sessions was reported in 24 trials, ranging from three to 56 sessions (median=6 sessions). No significant associations were detected between the number of sessions and SBP (β= -0.17, P=0.15), DBP (β= -0.15, P= 0.08), BMI (β= -0.01, P=0.57) and weight (β= 0.02, P=0.68). Interventions with more sessions were associated with slight reductions in serum total cholesterol (β=-0.01, P= 0.02). Thirteen of the included trials provided enough details to calculate intervention delivery duration, which ranged from 45 mins to 2.5 hrs (median=300 mins). No significant associations were detected between intervention duration and SBP (β=-0.00, P=0.26), DBP (β=-0.00, P= 0.45), BMI (β= -0.00, P= 0.53) and weight (β= -0.00, P=0.55). Hence, more sessions and longer intervention duration were not necessarily associated with greater intervention effectiveness.

Theory use: Of the 31 trials included, nine reported some use of psychological theory (or a combination of two theories) in relation to the intervention. The Transtheoretical Model [57] was used in eight trials [27 28 36-40 47], while Social Cognitive Theory [58] was used in four [27 28 38 59] interventions.

We tested the extent of theory use using Theory Coding Scheme (TCS) [20] in three ways (supplementary table 4). The first method was based on the use of theory in selecting intervention techniques (item 5 in TCS). Only four trials were coded yes for this item. The

For peer review only

second method was used to reflect the extent to which reported BCTs were linked to theory-relevant constructs (items 7 to 9). Only four trials were coded yes to at least one of these items. The third method was used to reflect the extent to which all theory-relevant constructs were targeted by BCTs (items 9 to 11). Only four trials were coded yes to at least one of these items. Therefore, we were not able to examine the impact of differing levels of theory use on intervention outcomes due to the small number of trials using theory extensively. However, we were able to test whether studies that merely reported using a theory had greater impact on outcomes using meta-regression. There was no significant association between studies which reported using a theory and SBP (β= -0.13, P= 0.89), DBP (β= -0.37, P= 0.73), and BMI (β= -0.03, P= 0.87). Studies that reported using a theory had increased weight (β= 1.07, P= 0.03, CI= 0.11, 2.04) and serum total cholesterol outcomes (β= 0.19, P= 0.04) compared to studies that did not report using a theory.

Effectiveness of specific behaviour change techniques: The number of behaviour change techniques (BCTs) in the intervention group varied, ranging from two to ten BCTs (median= 5). Behaviour change techniques in the control group were generally poorly described as the majority of trials (n= 16) did not appear to offer any BCTs.

Twenty nine different BCTs were identified from the included trials (supplementary table 2). The most commonly used BCTs in the intervention group were ‘credible source’ and ‘Goal setting (behaviour)’, which were used in 22 and 19 trials respectively. In the control group, ‘Credible source’ and ‘Information about health consequences’ were most commonly used, which were used in six and five interventions respectively.

We tested the potential impact of using specific BCTs on intervention outcomes (table 4). For SBP, one BCT had a significant influence on effect sizes. Interventions employing ‘Review of behaviour goal(s)’ resulted in an increase in SBP (β=3.45, P= 0.04) compared with those not using this BCT. For DBP and total cholesterol, there were no BCTs

For peer review only

significantly associated with the effectiveness of the interventions. The same was the case for BMI, but for weight, interventions that included 'Action planning' resulted in greater reductions than those that did not (β= -1.10, P= 0.04).

For peer review only

Table 4: Meta-regression results of intervention effects for studies using or not using particular behaviour change techniques.

Outcome	BCT	BCT included			BCT not included					
		MD	CI	N	MD	CI	N	β	CI	P
**Systolic blood pressure**	1.1 Goal setting (behaviour).	-1.12	-2.49 to 0.25	11	-3.87	-5.07 to -2.67	5	2.79	-0.19 to 5.78	0.07
	1.2 Problem solving.	-3.19	-9.21 to 2.83	3	-1.69	-2.99 to -0.39	13	-0.98	-5.12 to 3.16	0.62
	1.3 Goal setting (outcome).	-3.01	-6.99 to 0.97	3	-1.65	-3.12 to -0.18	13	-1.11	-4.95 to 2.73	0.55
	1.4 Action planning.	-2.84	-5.29 to -0.39	7	-1.39	-3.01 to 0.23	9	-1.39	-4.54 to 1.76	0.36
	**1.5 Review behaviour goal(s)**	**0.93**	**-2.10 to 3.95**	**4**	**-2.49**	**-3.82 to -1.16**	**12**	**3.45**	**0.13 to 6.76**	**0.04**
	4.1 Instruction on how to perform the behaviour.	-2.77	-4.89 to -0.68	5	-1.23	-2.89 to 0.44	11	-1.53	-4.53 to 1.47	0.29
	5.1 Information about health consequences.	-0.70	-2.13 to 0.73	5	-2.59	-4.43 to -0.76	11	1.75	-1.15 to 4.65	0.22
	9.1 Credible source.	-2.75	-4.34 to -1.17	9	-0.43	-2.81 to 1.96	7	-2.47	-5.51 to 0.58	0.10
	9.2 Pros and cons.	0.16	-3.89 to 4.20	4	-2.31	-3.59 to -1.02	12	2.67	-0.84 to 6.18	0.13
	11.2 Reduce negative emotions.	-3.52	-4.93 to -2.11	4	-1.05	-2.46 to 0.37	12	-0.22	-5.48 to 5.03	0.93
**Diastolic blood pressure**	1.1 Goal setting (behaviour).	-1.18	-2.31 to -0.04	10	-3.37	-3.78 to -0.96	5	1.35	-0.89 to 3.60	0.22
	1.4 Action planning.	-2.17	-4.13 to -0.20	6	-1.28	-2.30 to -0.25	9	-0.82	-3.02 to 1.38	0.44
	2.3 Self-monitoring of behaviour.	-1.29	-3.03 to 0.46	3	-1.58	-2.60 to -0.55	12	0.18	-2.73 to 3.08	0.89
	4.1 Instruction on how to perform the behaviour.	-1.12	-2.80 to 0.56	5	-1.84	-2.77 to -0.91	10	0.87	-1.17 to 2.91	0.38
	5.1 Information about health consequences.	-0.64	-2.52 to 1.24	4	-1.92	-2.79 to -1.05	11	1.46	-0.53 to 3.46	0.14
	9.1 Credible source.	-1.85	-3.44 to -0.26	9	-1.29	-1.91 to -0.68	6	-0.37	-2.51 to 1.77	0.72
	9.2 Pros and cons.	-1.46	-3.05 to 0.13	3	-0.93	-1.46 to 0.12	12	0.04	-2.73 to 2.80	0.98
	11.2 Reduced negative emotions.	-2.98	-4.71 to -1.25	4	-1.25	-2.25 to -0.25	11	-1.78	-4.46 to 0.89	0.17
**Serum total cholesterol**	1.1 Goal setting (behaviour).	-0.11	-0.17 to -0.5	9	-0.17	-0.34 to -0.01	5	0.09	-0.08 to 0.26	0.29
	1.3 Goal setting (outcome).	-0.21	-0.45 to 0.02	3	-0.10	-0.16 to -0.03	11	-0.12	-0.27 to 0.03	0.11
	1.4 Action planning.	-0.15	-0.34 to 0.05	6	-0.12	-0.18 to -0.06	8	-0.05	-0.22 to -0.12	0.52
	4.1 Instruction on how to perform the behaviour.	-0.18	-0.19 to -0.07	4	-0.10	-0.17 to -0.04	10	-0.09	-0.25 to 0.06	0.20
	5.1 Information about health consequences.	-0.11	-0.18 to -0.07	5	-0.15	-0.28 to -0.03	9	0.07	-0.08 to -0.22	0.35
	9.1 Credible source.	-0.12	-0.21 to -0.04	9	-0.14	-0.22 to -0.05	5	-0.00	-0.18 to 0.17	0.96
	9.2 Pros and cons.	-0.07	-0.31 to 0.16	3	-0.14	-0.20 to -0.07	11	0.05	-0.18 to 0.28	0.64
**Body mass index**	1.1 Goal setting (behaviour).	-0.24	-0.42 to -0.05	10	-0.03	-0.21 to 0.15	4	-0.20	-0.49 to 0.08	0.14
	1.3 Goal setting (outcome).	-0.09	-0.50 to 0.32	3	-0.14	-0.27 to -0.00	11	0.05	-0.42 to 0.52	0.83

For peer review only

	1.4 Action planning.	-0.32	-0.61 to -0.04	6	-0.09	-0.23 to 0.06	8	-0.24	-0.59 to 0.11	0.17
	1.5 Review behaviour goal(s)	-0.66	-1.51 to 0.20	3	-0.12	-0.25 to 0.01	11	-0.54	-1.48 to 0.41	0.25
	4.1 Instructions on how to perform the behaviour.	-0.07	-0.23 to 0.09	5	-0.24	-0.45 to -0.03	9	0.17	-0.12 to 0.46	0.23
	5.1 Information about health consequences.	-0.13	-0.36 to 0.10	4	-0.14	-0.29 to 0.02	10	0.01	-0.29 to 0.31	0.96
	9.1 Credible source.	-0.24	-0.43 to -0.06	8	-0.03	-0.21 to 0.15	6	-0.21	-0.49 to 0.07	0.13
	9.2 Pros and cons.	-0.47	-1.29 to 0.34	3	-0.13	-0.26 to 0.01	11	-0.35	-1.25 to 0.55	0.42
	11.2 Reduce negative emotions.	-0.33	-0.79 to 0.14	3	-0.12	-0.25 to 0.02	11	-0.21	-0.74 to 0.32	0.41
**Weight**	1.3 Goal setting (outcome)	-1.02	-1.73 to -0.31	4	-0.83	-1.53 to -0.12	8	-0.17	-1.33 to 0.99	0.75
	**1.4 Action planning.**	**-1.27**	**-1.74 to -0.79**	**7**	**-0.17**	**-0.94 to 0.58**	**5**	**-1.10**	**-2.11 to -0.09**	**0.04**
	1.5 Review behaviour goal(s)	-1.67	-4.77 to 1.40	3	-0.86	-1.42 to -0.29	9	-0.82	-4.41 to 2.77	0.62
	2.3 Self-monitoring of behaviour.	-0.91	-2.13 to 0.31	3	-0.89	-1.55 to -0.25	9	-0.04	-1.27 to 1.10	0.95
	4.1 Instruction on how to perform the behaviour.	-0.81	-1.57 to -0.05	5	-0.99	-1.77 to -0.22	7	0.17	-1.08 to 1.42	0.77
	9.1 Credible source.	-0.95	-1.52 to -0.38	8	-0.27	-1.89 to 1.35	4	-0.65	-2.66 to 1.36	0.49
	9.2 Pros and cons.	-0.91	-3.91 to 2.09	3	-0.88	-1.48 to -0.28	9	-0.01	-3.51 to 3.49	0.99

Note: BCT, behaviour change technique; MD, mean difference; CI, 95% confidence interval; N, number of trials; β, meta-regression coefficient

For peer review only

DISCUSSION

This systematic review is among the first to evaluate the impact of theory use and BCTs in MHBC interventions for reducing CVD-risk. Although pooled effects of interventions on risk factors were statistically significant but clinically modest. The results of this systematic review suggest that MHBC interventions evaluated to date for the primary prevention of CVD may generally have very limited effects in reducing CVD-risk and CVD risk factors in primary care populations.

Previous systematic reviews have investigated the effectiveness of interventions aimed at individual risk factors including diet, physical activity and body weight [6 60]. These reviews generally find that behaviour change interventions in primary care have minor impact on risk factors values. The Cochrane review up to 2011 reported modest reductions in CVD risk factors following MHBC interventions that were slightly greater than we report [5]. However, the Cochrane review did not restrict the intervention setting to primary care.

Estimated changes in CVD risk factors should be viewed with caution. In the present set of trials, the average duration of follow-up was 12 months and changes in risk factors observed may be unlikely to reflect changes occurring over longer periods. This review found reductions in blood pressure and total cholesterol following intervention, but in some instances this might be mediated by pharmacological treatment. There are clear benefits of drug treatments in lowering blood pressure and cholesterol in primary prevention populations [61 62].

Although this review focused on interventions for the primary prevention population, we also included trials that recruited a small minority of participants with some evidence of CVD.

For peer review only

Including these trials might have biased the results, as health promotion interventions might have more positive effects in people with established cardiovascular disease [63-65].

In order to account for heterogeneity, we focused on trial level covariates and identified characteristics that might be associated with more favourable outcomes. When coding BCTs, we were limited by the lack of detail provided in reports. We only coded what was explicitly referred to in intervention descriptions and could be fitted to BCT taxonomy definitions.

This review suggested no association between the number of intervention sessions or intervention duration and improved outcomes. Quantity of sessions would not necessarily have a beneficial impact on outcomes unless additional sessions deliver BCTs that effectively influence behaviours. Few reports provided sufficient information to permit calculating duration for analysis. Increasing use of the TIDieR checklist [66], requiring intervention reports to detail the number and duration of sessions offered to participants, will be helpful for future reviews.

Our analyses suggested that using certain BCTs has a moderator effect on intervention outcomes. In terms of biomarkers of CVD risk, no BCTs were identified as being particularly likely to influence cholesterol levels, while including review of behaviour goals appeared to be associated with slightly worse blood pressure outcomes.

“Action planning” was associated with greater weight loss, while “instruction on how to perform the behaviour” was not. Both of these findings differ to those of a previous review [17], perhaps because it focused only on interventions for obese individuals. The previous review also identified the BCTs of self-monitoring, relapse prevention/problem solving and prompt

For peer review only

practice as beneficial to weight loss, but too few of the interventions included in the present review incorporated these BCTs for it to be possible to test their influence. A review of interventions promoting healthy eating and exercise also found that including the BCT of self-monitoring was associated with bigger changes in these behaviours [18]. Therefore, one explanation for the relatively limited effectiveness of the interventions reviewed in the present review is that they failed to include BCTs that were more likely to lead to health-promoting changes. A second possibility is that not all BCTs were delivered as the intervention designers intended. This cannot be ruled out as monitoring of treatment fidelity was rarely described in the included studies.

This review showed no association between the use of psychological theory and improved intervention outcomes. However, only a limited range of theories were employed – mostly the Transtheoretical Model and Social Cognitive Theory. A previous review also found that interventions based on these theories were not significantly more effective than interventions not explicitly based on theory [13]. A second issue is that the links between the psychological determinants specified by a theory and the BCTs employed in interventions were sometimes poorly articulated, with little evidence cited to justify choice of BCTs to change specific constructs. Furthermore, it was not always clear which BCTs were being used to target which behaviours as part of the MHBC interventions. Both this and previous reviews [13 67] found that reported theory use in intervention design was not as extensive as it could be. It is possible that interventions based on other theories or that more explicitly link theoretical constructs to select BCTs might be more effective.

Future trials need to test interventions that provide explicit links between intervention components (i.e. theoretical basis, BCTs and intended mechanisms of action, intervention duration) and intervention outcomes as it is essential step towards understanding MHBC intervention effects. Higher priority should also be given to different population-level approaches to facilitate behaviour change.

For peer review only

Limitations

The results of this review must be viewed with caution because of several limitations. First, the observed effects were heterogeneous, therefore pooled estimates might be questionable. DerSimonian and Laird (DL) [22] random effects models were used. The DL method may lead to under-estimation of between trial variance leading to narrower confidence intervals in the presence of heterogeneity[68 69]. However, Thorlund, et al. [70] concluded that inferences concerning pooled effects were only infrequently influenced by the choice of between-trial variance estimator. The majority of trials included were undertaken in Europe (71%) and the United States (13%). Declines in CVD mortality and CVD-risk have been observed in these countries, and the results should be considered in the context of these trends. Groups of BCTs may have synergistic effects on behaviour [16]. However, due to the relatively small numbers of studies and under-description of the BCTs used in interventions, it was not possible to explore the impact of clusters of BCTs on CVD risk factors, as too few studies used the same clusters of BCTs and measured the same outcome. Furthermore, the differences between subgroups and covariates (i.e. theory use and BCTs) and effect size are observational and do not imply causality. Behavioural risk factors were assessed by self-report and so values were subject to social desirability and recall biases. Finally, as this review involved testing for the impact of MHBC interventions and intervention characteristics on intervention outcomes, we are aware of the need to adjust p-values based on the number of tests being made [71]. Although adjusting p-values reduces type 1 error, it increases the chances of false negatives [72]. Furthermore, tests were examining independent hypotheses, therefore p-values were not adjusted[73].

For peer review only

CONCLUSION

Existing multiple health behaviour change interventions delivered to individual participants in primary care appear to have limited effectiveness at reducing CVD-risk and CVD risk factors over twelve months or longer. Trial reports need to provide explicit explanation of the intervention theory, content and delivery, including fidelity and care provided to the control group in order to understand why an intervention may or may not prove effective. This is essential for future development and evaluation of effective CVD prevention interventions.

Funding:

MG was supported by the National Institute for Health Research (NIHR) Biomedical Research Centre at Guy's and St Thomas' NHS Foundation Trust and King's College London. SA was supported by the Government of Saudi Arabia.

Contributorship statement:

SA, AW and MG conceptualised and designed the study. SA and MG performed the paper search. SA and LM performed the coding. SA wrote the first draft and all authors have read and made improvements of the contents and the wording.

Competing interests:

There are no competing interests.

Data sharing statement:

No additional data are available.

For peer review only

References

1. WHO. Cardiovascular diseases (CVDs). Accessed August, 2016 http://www.who.int/mediacentre/factsheets/fs317/en/.

2. Poortinga W. The prevalence and clustering of four major lifestyle risk factors in an English adult population. *Preventive medicine* 2007;44(2):124-28. doi.org/10.1016/j.ypmed.2006.10.006.

3. Cairney J, Leatherdale ST, Faulkner GE. A longitudinal examination of the interrelationship of multiple health behaviors. *American journal of preventive medicine* 2014;47(3):283-89. doi.org/10.1016/j.amepre.2014.04.019.

4. Khaw K-T, Wareham N, Bingham S, et al. Combined impact of health behaviours and mortality in men and women: the EPIC-Norfolk prospective population study. *PLoS medicine* 2008;5(1):e12. doi.org/10.1371/journal.pmed.0050012.

5. Ebrahim S, Taylor F, Ward K, et al. Multiple risk factor interventions for primary prevention of coronary heart disease. *The Cochrane Library 2011.* doi.org/10.1002/14651858.CD001561.pub3.

6. Fleming P, Godwin M. Lifestyle interventions in primary care Systematic review of randomized controlled trials. *Canadian family physician* 2008;54(12):1706-13.

7. Álvarez-Bueno C, Cavero-Redondo I, Martínez-Andrés M, et al. Effectiveness of multifactorial interventions in primary health care settings for primary prevention of cardiovascular disease: a systematic review of systematic reviews. *Preventive medicine* 2015;76:S68-S75. doi.org/10.1016/j.ypmed.2014.11.028.

8. Krogsbøll LT, Jørgensen KJ, Larsen CG, et al. General health checks in adults for reducing morbidity and mortality from disease: Cochrane systematic review and meta-analysis. *BMJ* 2012;345:e7191. doi.org/10.1136/bmj.e7191.

9. Michie S, Richardson M, Johnston M, et al. The behavior change technique taxonomy (v1) of 93 hierarchically clustered techniques: building an international consensus for the reporting of behavior change interventions. *Annals of behavioral medicine* 2013;46(1):81-95. doi.org/10.1007/s12160-013-9486-6.

10. Campbell M, Fitzpatrick R, Haines A, et al. Framework for design and evaluation of complex interventions to improve health. *BMJ* 2000;321(7262):694-96. doi.org/10.1136/bmj.321.7262.694.

11. Michie S, Johnston M, Francis J, et al. From theory to intervention: mapping theoretically derived behavioural determinants to behaviour change techniques. *Applied psychology* 2008;57(4):660-80. doi.org/10.1111/j.1464-0597.2008.00341.x.

12. Webb T, Joseph J, Yardley L, et al. Using the internet to promote health behavior change: a systematic review and meta-analysis of the impact of theoretical basis, use of behavior change techniques, and mode of delivery on efficacy. *Journal of medical Internet research* 2010;12(1):e4. doi.org/10.2196/jmir.1376.

For peer review only

13. Prestwich A, Sniehotta FF, Whittington C, et al. Does theory influence the effectiveness of health behavior interventions? Meta-analysis. *Health Psychology* 2014;33(5):465. doi.org/10.1037/a0032853.

14. Prestwich A, Webb TL, Conner M. Using theory to develop and test interventions to promote changes in health behaviour: evidence, issues, and recommendations. *Current Opinion in Psychology* 2015;5:1-5. doi.org/10.1016/j.copsyc.2015.02.011.

15. Michie S, Atkins L, West R. The behaviour change wheel: a guide to designing interventions. Great Britain: Silverback Publishing 2015.

16. Michie S, Fixsen D, Grimshaw JM, et al. Specifying and reporting complex behaviour change interventions: the need for a scientific method. *Implement Sci* 2009;4(40):1-6. doi.org/10.1186/1748-5908-4-40.

17. Dombrowski SU, Sniehotta FF, Avenell A, et al. Identifying active ingredients in complex behavioural interventions for obese adults with obesity-related co-morbidities or additional risk factors for co-morbidities: a systematic review. *Health Psychology Review* 2012;6(1):7-32. doi.org/10.1080/17437199.2010.513298. doi.org/10.1080/17437199.2010.513298.

18. Michie S, Abraham C, Whittington C, et al. Effective techniques in healthy eating and physical activity interventions: a meta-regression. *Health Psychology* 2009;28(6):690. doi.org/10.1037/a0016136.

19. Higgins JP, Altman DG, Gøtzsche PC, et al. The Cochrane Collaboration's tool for assessing risk of bias in randomised trials. *BMJ* 2011;343. doi.org/10.1136/bmj.d5928.

20. Michie S, Prestwich A. Are interventions theory-based? Development of a theory coding scheme. *Health Psychology* 2010;29(1):1. doi.org/10.1037/a0016939.

21. Bishop FL, Fenge-Davies AL, Kirby S, et al. Context effects and behaviour change techniques in randomised trials: A systematic review using the example of trials to increase adherence to physical activity in musculoskeletal pain. *Psychology & health* 2015;30(1):104-21. doi.org/10.1080/08870446.2014.953529.

22. DerSimonian R, Laird N. Meta-analysis in clinical trials. *Controlled clinical trials* 1986;7(3):177-88.

23. Egger M, Smith GD, Schneider M, et al. Bias in meta-analysis detected by a simple, graphical test. *BMJ* 1997;315(7109):629-34.

24. Duval S, Tweedie R. A nonparametric "trim and fill" method of accounting for publication bias in meta-analysis. *Journal of the American Statistical Association* 2000;95(449):89-98.

25. Mendis S, Johnston SC, Fan W, et al. Cardiovascular risk management and its impact on hypertension control in primary care in low-resource settings: a cluster-randomized trial. *Bulletin of the World Health Organization* 2010;88(6):412-19.

For peer review only

26. Lakerveld J, Bot SD, Chinapaw MJ, et al. Motivational interviewing and problem solving treatment to reduce type 2 diabetes and cardiovascular disease risk in real life: a randomized controlled trial. *Int J Behav Nutr Phys Act* 2013;10(47):10.1186. doi.org/10.1186/1479-5868-10-47.

27. Hardcastle SJ, Taylor AH, Bailey MP, et al. Effectiveness of a motivational interviewing intervention on weight loss, physical activity and cardiovascular disease risk factors: a randomised controlled trial with a 12-month post-intervention follow-up. *Int J Behav Nutr Phys Act* 2013;10(40):1-16. doi.org/10.1186/1479-5868-10-40.

28. Parra-Medina D, Wilcox S, Salinas J, et al. Results of the Heart Healthy and Ethnically Relevant Lifestyle trial: a cardiovascular risk reduction intervention for African American women attending community health centers. *American journal of public health* 2011;101(10):1914-21. doi.org/ 10.2105/AJPH.2011.300151.

29. Harting J, van Assema P, van Limpt P, et al. Cardiovascular prevention in the Hartslag Limburg project: effects of a high-risk approach on behavioral risk factors in a general practice population. *Preventive medicine* 2006;43(5):372-78. doi.org/10.1016/j.ypmed.2006.06.016.

30. OXCHECK. Effectiveness of health checks conducted by nurses in primary care: final results of the OXCHECK study. *BMJ* 1995:1099-104. doi.org/10.1136/bmj.310.6987.1099.

31. Knutsen SF, Knutsen R. The Tromsø Survey: the Family Intervention study—the effect of intervention on some coronary risk factors and dietary habits, a 6-year follow-up. *Preventive medicine* 1991;20(2):197-212. doi.org/10.1016/0091-7435(91)90020-5.

32. Meland E, Lærum E, Ulvik RJ. Effectiveness of two preventive interventions for coronary heart disease in primary care. *Scandinavian journal of primary health care* 1997;15(1):57-63. doi.org/10.3109/02813439709043432.

33. Sartorelli DS, Sciarra EC, Franco LJ, et al. Beneficial effects of short-term nutritional counselling at the primary health-care level among Brazilian adults. *Public health nutrition* 2005;8(07):820-25. doi.org/10.1079/PHN2005737

34. Koelewijn-van Loon MS, van der Weijden T, van Steenkiste B, et al. Involving patients in cardiovascular risk management with nurse-led clinics: a cluster randomized controlled trial. *Canadian Medical Association Journal* 2009;181(12):E267-E74. doi.org/10.1503/cmaj.081591.

35. Brett T, Arnold-Reed D, Phan C, et al. The Fremantle Primary Prevention Study: a multicentre randomised trial of absolute cardiovascular risk reduction. *Br J Gen Pract* 2012;62(594):e22-e28. doi.org/ 10.3399/bjgp12X616337.

36. Steptoe A, Day S, Doherty S, et al. Behavioural counselling in general practice for the promotion of healthy behaviour among adults at increased risk of coronary heart disease: randomised trialCommentary: Treatment allocation by the method of minimisation. *BMJ* 1999;319(7215):943-48.

37. Eriksson MK, Franks PW, Eliasson M. A 3-year randomized trial of lifestyle intervention for cardiovascular risk reduction in the primary care setting: the Swedish Björknäs study. *PloS one* 2009;4(4):e5195. doi.org/10.1371/journal.pone.0005195.

For peer review only

38. Ma J, Berra K, Haskell WL, et al. Case management to reduce risk of cardiovascular disease in a county health care system. *Archives of internal medicine* 2009;169(21):1988-95. doi.org//10.1001_archinternmed.2009.381.

39. Tiessen AH, Smit AJ, Broer J, et al. Randomized controlled trial on cardiovascular risk management by practice nurses supported by self-monitoring in primary care. *BMC family practice* 2012;13(1):1. doi.org//10.1186/1471-2296-13-90.

40. Drevenhorn E, Bengtson A, Nilsson PM, et al. Consultation training of nurses for cardiovascular prevention–a randomized study of 2 years duration. Blood pressure 2012;**21**(5):293-99.

41. Wennehorst K, Mildenstein K, Saliger B, et al. A comprehensive lifestyle intervention to prevent type 2 diabetes and cardiovascular diseases: The german chip trial. *Prevention Science* 2016.

42. Gomez-Huelgas R, Jansen-Chaparro S, Baca-Osorio A, et al. Effects of a long-term lifestyle intervention program with Mediterranean diet and exercise for the management of patients with metabolic syndrome in a primary care setting. *European journal of internal medicine* 2015;26(5):317-23.

43. Duncan S, Goodyear-Smith F, McPhee J, et al. Family-centered brief intervention for reducing obesity and cardiovascular disease risk: A randomized controlled trial. *Obesity* 2016;24(11):2311-18.

44. Salisbury C, O'Cathain A, Thomas C, et al. Telehealth for patients at high risk of cardiovascular disease: pragmatic randomised controlled trial. *BMJ* 2016;353:i2647.

45. Baron JA, Gleason R, Crowe B, et al. Preliminary trial of the effect of general practice based nutritional advice. *Br J Gen Pract* 1990;40(333):137-41.

46. Kranjčević K, Marković BB, Lalić DI, et al. Is a targeted and planned GP intervention effective in cardiovascular disease prevention? A randomized controlled trial. *Medical science monitor: international medical journal of experimental and clinical research* 2014;20:1180. doi.org/10.12659/MSM.890242.

47. Harris MF, Fanaian M, Jayasinghe UW, et al. A cluster randomised controlled trial of vascular risk factor management in general practice. *Med J Aust* 2012;197(7):387-93. doi.org/10.5694/mja12.10313.

48. Korhonen M, Kastarinen M, Uusitupa M, et al. The effect of intensified diet counseling on the diet of hypertensive subjects in primary health care: a 2-year open randomized controlled trial of lifestyle intervention against hypertension in eastern Finland. *Preventive medicine* 2003;36(1):8-16. doi.org/10.1006/pmed.2002.1120.

49. Nilsson PM, Lindholm LH, Scherstén BF. Life style changes improve insulin resistance in hyperinsulinaemic subjects: a one-year intervention study of hypertensives and normotensives in Dalby. *Journal of hypertension* 1992;10(9):1071-78.

50. Avram C, Iurciuc M, Craciun L, et al. Dietary and physical activity counseling in high-risk asymptomatic patients with metabolic syndrome–A primary care intervention. *Journal of Food, Agriculture & Environment* 2011;9(3&4):16-19.

For peer review only

51. Lindholm LH, Ekbom T, Dash C, et al. The impact of health care advice given in primary care on cardiovascular risk. *BMJ* 1995;310(6987):1105-09.

52. Kannel WB, McGee D, Gordon T. A general cardiovascular risk profile: the Framingham Study. *The American journal of cardiology* 1976;38(1):46-51.

53. Wood D, Kinmonth A, Davies G, et al. Randomised controlled trial evaluating cardiovascular screening and intervention in general practice: principal results of British family heart study. *BMJ* 1994;308(6924):313-20. doi.org/10.1136/bmj.308.6924.313

54. Tunstall-Pedoe H. The Dundee coronary risk-disk for management of change in risk factors. BMJ 1991;303(6805):744-47.

55. Collins GS, Altman DG. Predicting the 10 year risk of cardiovascular disease in the United Kingdom: independent and external validation of an updated version of QRISK2. *BMJ* 2012;344:e4181.

56. Conroy R, Pyörälä K, Fitzgerald Ae, et al. Estimation of ten-year risk of fatal cardiovascular disease in Europe: the SCORE project. *European heart journal* 2003;24(11):987-1003. http://dx.doi.org/10.1016/S0195-668X(03)00114-3.

57. Prochaska JO, Norcross JC. Stages of change. *Psychotherapy: Theory, research, practice, training* 2001;38(4):443.

58. Bandura A. *Social foundations of thought and action: A social cognitive theory*: Prentice-Hall, Inc, 1986.

59. Vetter ML, Wadden TA, Chittams J, et al. Effect of lifestyle intervention on cardiometabolic risk factors: results of the POWER-UP trial. *International Journal of Obesity* 2013;37:S19-S24. doi.org/10.1038/ijo.2013.92.

60. Booth HP, Prevost TA, Wright AJ, et al. Effectiveness of behavioural weight loss interventions delivered in a primary care setting: a systematic review and meta-analysis. *Family practice* 2014;31(6):643-53. doi.org/10.1093/fampra/cmu064.

61. Trialists CT. Efficacy and safety of cholesterol-lowering treatment: prospective meta-analysis of data from 90 056 participants in 14 randomised trials of statins. *The Lancet* 2005;366(9493):1267-78. doi.org/10.1016/S0140-6736(05)67394-1.

62. Taylor F, Huffman MD, Macedo AF, et al. Statins for the primary prevention of cardiovascular disease. *The Cochrane Library* 2013. doi.org/10.1002/14651858.CD004816.pub4.

63. Oldridge NB, Guyatt GH, Fischer ME, et al. Cardiac rehabilitation after myocardial infarction: combined experience of randomized clinical trials. *JAMA* 1988;260(7):945-50. doi.org/10.1001/jama.1988.03410070073031.

64. Taylor RS, Brown A, Ebrahim S, et al. Exercise-based rehabilitation for patients with coronary heart disease: systematic review and meta-analysis of randomized controlled trials. *The American journal of medicine* 2004;116(10):682-92. doi.org/10.1016/j.amjmed.2004.01.009.

For peer review only

65. Mullen PD, Mains DA, Velez R. A meta-analysis of controlled trials of cardiac patient education. *Patient education and counseling* 1992;19(2):143-62. .doi.org/10.1016/0738-3991(92)90194-N.

66. Hoffmann TC, Glasziou PP, Boutron I, et al. Better reporting of interventions: template for intervention description and replication (TIDieR) checklist and guide. *BMJ* 2014;348:g1687. doi.org/10.1136/bmj.g1687.

67. Michie S, Jochelson K, Markham WA, et al. Low income groups and behaviour change interventions: a review of intervention content, effectiveness and theoretical frameworks. *Journal of Epidemiology and Community Health* 2009:jech. 2008.078725. doi.org/10.1136/jech.2008.078725.

68. Jackson D, Bowden J, Baker R. How does the DerSimonian and Laird procedure for random effects meta-analysis compare with its more efficient but harder to compute counterparts? *Journal of Statistical Planning and Inference* 2010;140(4):961-70.

69. Cornell JE, Mulrow CD, Localio R, et al. Random-effects meta-analysis of inconsistent effects: a time for change. *Annals of internal medicine* 2014;160(4):267-70.

70. Thorlund K, Wetterslev J, Awad T, et al. Comparison of statistical inferences from the DerSimonian–Laird and alternative random-effects model meta-analyses–an empirical assessment of 920 Cochrane primary outcome meta-analyses. *Research synthesis methods* 2011;2(4):238-53.

71. Bland JM, Altman DG. Multiple significance tests: the Bonferroni method. *BMJ* 1995;310(6973):170. doi.org/10.1136/bmj.310.6973.170.

72. Feise RJ. Do multiple outcome measures require p-value adjustment? *BMC medical research methodology* 2002;2(1):8.

73. Rothman KJ, Greenland S, Lash TL. *Modern epidemiology*: Lippincott Williams & Wilkins, 2008.

For peer review only

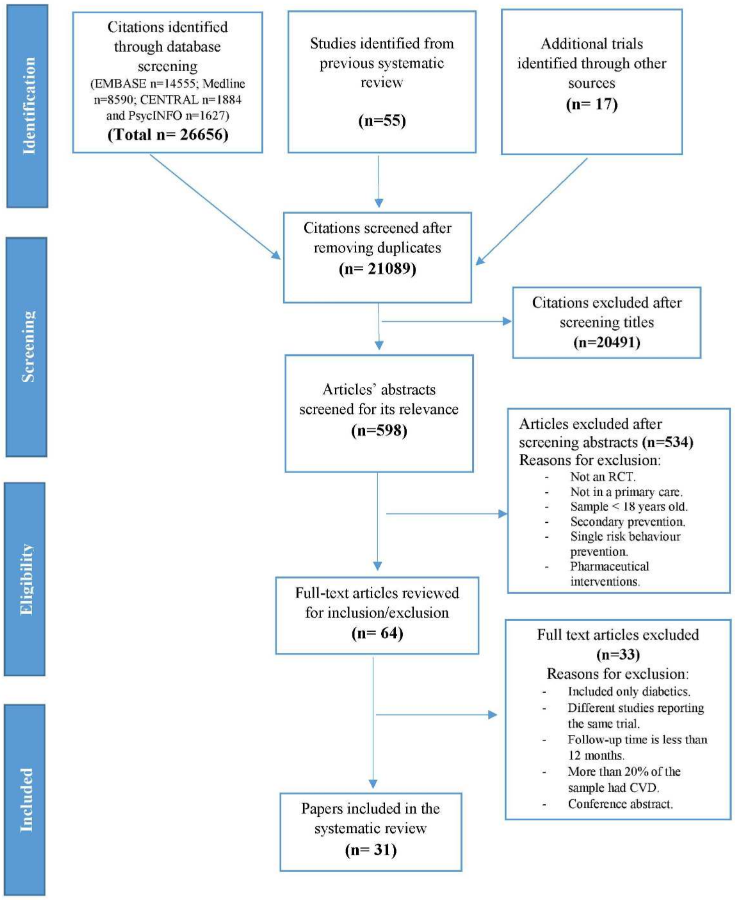

Figure 1: PRISMA flow diagram outlining the systematic review processes.

Figure 1: PRISMA flow diagram outlining the systematic review processes.

210x297mm (300 x 300 DPI)

Appendix A

Search strategy

CENTRAL search strategy

ID	Search	Hits
#1	MeSH descriptor CARDIOVASCULAR DISEASES this term only	480
#2	MeSH descriptor CORONARY DISEASE explode all trees	356
#3	cardiovascular in All Text	2052
#4	(coronary in All Text near/3 disease* in All Text)	9
#5	(heart in All Text near/3 disease* in All Text)	11
#6	MeSH descriptor HYPERTENSION this term only	643
#7	hypertension in All Text	1781
#8	(atherosclerosis in All Text or arteriosclerosis in All Text)	258
#9	(hyperlipidaemia in All Text or hyperlipidemia in All Text)	224
#10	MeSH descriptor ARTERIOSCLEROSIS explode all trees	79
#11	MeSH descriptor CHOLESTEROL explode trees all trees	209
#12	MeSH descriptor HYPERLIPIDEMIA explode all trees	33
#13	cholesterol in All Text	630
#14	multiple next risk next factor* in All Text	51
#15	coronary next risk next factor* in All Text	30
#16	(#1 or #2 or #3 or #4 or #5 or #6 or #7 or #8 or #9 or #10)	3105
#17	(#11 or #12 or #13 or #14 or #15)	682
#18	(#16 or #17)	3234
#19	MeSH descriptor HEALTH EDUCATION explode all trees	630
#20	MeSH descriptor HEALTH PROMOTION explode all trees	191
#21	MeSH descriptor HEALTH BEHAVIOR explode all trees	215
#22	MeSH descriptor PRIMARY PREVENTION this term only	1021
#23	MeSH descriptor COUNSELLING this term only	237
#24	counsel* in All Text	1186
#25	(health in All Text near/3 educat* in All Text)	31
#26	(patient in All Text near/3 educat* in All Text)	20
#27	(education* in All Text near/3 program* in All Text)	23
#28	(health in All Text near/3 promotion* in All Text)	2
#29	(health in All Text near/3 behaviour* in All Text)	11
#30	(health in All Text near/3 behavior* in All Text)	9
#31	primary next prevention in All Text	379
#32	(multiple next risk in All Text near/3 intervention* in All Text)	6
#33	(multifactor* in All Text near/3 intervention* in All Text)	9
#34	(multifactor* in All Text near/3 prevention in All Text)	1
#35	(risk next factor* in All Text near/3 reduc* in All Text)	10
#36	(risk next factor* in All Text near/3 manag* in All Text)	20
#37	(risk next factor* in All Text near/3 intervent* in All Text)	49

For peer review only

#38 (lifestyle in All Text near/3 intervention* in All Text) 34
#39 (lifestyle in All Text near/3 advice in All Text) 6
#40 (life-style in All Text near/3 intervention* in All Text) 12
#41 (life-style in All Text near/3 advice in All Text) 2
#42 (life-style in All Text near/3 alter* in All Text) 1
#43 (lifestyle in All Text near/3 alter* in All Text) 5
#44 (lifestyle in All Text near/3 educat* in All Text) 15
#45 (life-style in All Text near/3 educat* in All Text) 5
#46 (life-style in All Text near/3 chang* in All Text) 8
#47 (lifestyle in All Text near/3 chang* in All Text) 18
#48 (behavior* in All Text near/3 chang* in All Text) 24
#49 (behaviour* in All Text near/3 chang* in All Text) 37
#50 (health next care in All Text near/3 advice in All Text) 7
#51 (healthcare in All Text near/3 advice in All Text) 8
#52 nonpharmacologic* in All Text 46
#53 non-pharmacologic* in All Text 562
#54 (#19 or #20 or #21 or #22 or #23 or #24 or #25 or #26 or #27 or #28 or #29) 2311
#55 (#30 or #31 or #32 or #33 or #34 or #35 or #36 or #37 or #38 or #39) 451
#56 (#40 or #41 or #42 or #43 or #44 or #45 or #46 or #47 or #48 or #49 or #50 or #51 or #52 or #53) 646
#57 (#54 or #55 or #56) 2915
#58 (#18 and #57) 1293

Embase search strategy

1. cardiovascular disease/
2. exp ischemic heart disease/
3. (Coronary adj3 disease$).tw.
4. heart disease$.tw.
5. Hypertension/
6. hypertension.tw.
7. (cardiovascular adj3 (disease$ or fit of fitness)).tw.
8. exp arteriosclerosis/
9. exp hyperlipidemia/
10. hyperlipid?emia.tw.
11. cholesterol.tw.
12. arterioscler0$.tw.
13. atheroscler0$.tw.
14. coronary risk factor$.tw.
15. multiple risk factor$.tw.
16. cardiovascular risk factor$.tw.
17. or/1-16
18. exp health education/
19. exp health behavior/

For peer review only

20. primary prevention/
21. exp counseling/
22. (multifactor$ adj5 (intervent$ or prevent$)).tw.
23. ((life-style or life style or lifestyle or healthcare or health care) adj3 (intervention$ or educat$ or advice or alter$ or change$)).tw.
24. primary prevention.tw.
25. (risk factor$ adj3 (reduc$ or manage$ or managing or intervent$ or program$)).tw.
26. (educat$ adj3 (program$ or patient$)).tw.
27. (non pharmacologic$ or nonpharmacologic$).tw.
28. (risk factor$ adj3 modif$).tw.
29. ((lifestyle or life-style or life style) adj3 modif$).tw.
30. exp behavior therapy/
31. (behavi?r$ adj3 (intervention$ or program$ or modif$ or change$ or alter$)).tw.
32. (promot$ adj3 (health or healthcare or health care)).tw.
33. or/18-32
34. 17 and 33
35. random$.ti,ab.
36. factorial$.ti,ab.
37. (crossover$ or cross over$ or cross-over$).ti,ab.
38. placebo$.ti,ab.
39. (double$ adj blind$).ti,ab.
40. (singl$ adj blind$).ti,ab.
41. assign$.ti,ab.
42. allocat$.ti,ab.
43. volunteer$.ti,ab.
44. Crossover Procedure/
45. Double Blind Procedure/
46. Randomized Controlled Trial/
47. Single Blind Procedure/
48. or/35-47
49. exp animal/
50. nonhuman/
51. exp animal experiment/
52. or/49-51
53. exp human/
54. 52 not 53
55. 48 not 54
56. 55 and 34
57. limit 56 to yr="2006 -Current"

Medline search strategy

1. Cardiovascular Diseases/
2. exp coronary disease/
3. Hypertension/

For peer review only

4. exp Arteriosclerosis/
5. exp Hyperlipidemia/
6. (cardiovascular adj3 disease$).tw.
7. (cardiovascular adj3 (fit or fitness)).tw.
8. (Coronary adj3 disease$).tw.
9. heart disease$.tw.
10. hypertension.tw.
11. hyperlipid?emia.tw.
12. cholesterol.tw.
13. atherosclerosis.tw.
14. arteriosclerosis.tw.
15. coronary risk factor$.tw.
16. multiple risk factor$.tw.
17. cardiovascular risk factor$.tw.
18. or/1-17
19. health promotion/
20. exp health education/
21. exp health behavior/
22. exp counseling/
23. Primary Prevention/
24. (multifactor$ adj5 (intervent$ or prevent$)).tw.
25. ((lifestyle or life-style) adj3 (intervention$ or educat$ or advice$ or alter$ or change$)).tw.
26. ((lifestye or life-style or behavior?r$) adj3 (intervention$ or educat$ or advice$ or alter$ or change$)).tw.
27. ((healthcare or health care) adj3 advice).tw.
28. primary prevention.tw.
29. (risk factor$ adj3 (reduc$ or manage$ or managing or intervent$ or program$)).tw.
30. (educat$ adj3 (program$ or patient$)).tw.
31. ((health or healthcare or health care) adj3 (educat$ or advice or promot$)).tw.
32. (nonpharmacologic$ or non-pharmacologic$).tw.
33. ((lifestyle or life style or life-style or behavio?r$ or risk factor$) adj3 modif$).tw.
34. or/19-33
35. 18 and 34
36. randomized controlled trial.pt.
37. controlled clinical trial.pt.
38. Randomized controlled trials/
39. random allocation.sh.
40. double blind method.sh.
41. single-blind method.sh.
42. or/36-41
43. clinical trial.pt.
44. exp Clinical trial/
45. (clin$ adj25 trial$).ti,ab.

For peer review only

46. ((singl$ or doubl$ or trebl$ or tripl$) adj (blind$ or mask$)).ti,ab.
47. placebos.sh.
48. placebo$.ti,ab.
49. random$.ti,ab.
50. research design.sh.
51. or/43-50
52. exp animal/ not humans/
53. 42 or 51
54. 53 not 52
55. 54 and 35

PsycINFO search strategy:

1. cardiovascular disease.mp.
2. hypertension.mp.
3. (Coronary adj3 disease$).mp.
4. heart disease$.mp.
5. (cardiovascular adj3 (disease$ or fit of fitness)).mp. [mp=title, abstract, heading word, table of contents, key concepts, original title, tests & measures]
6. exp Arteriosclerosis/
7. hyperlipid?emia.mp.
8. cholesterol.mp.
9. arterioscler0$.mp.
10. atheroscler0$.mp.
11. coronary risk factor$.mp.
12. multiple risk factor$.mp.
13. cardiovascular risk factor$.mp.
14. or/1-13
15. exp health education/
16. exp health education/
17. exp health promotion/
18. exp preventive medicine/
19. exp counseling/
20. primary prevention.mp.
21. (multifactor$ adj5 (intervent$ or prevent$)).mp.
22. behavior change.mp.
23. exp Obesity/ or exp Food Intake/ or diet intervention.mp. or exp Weight Loss/ or exp Diets/ or exp Overweight/ or exp Weight Control/ or exp Nutrition/
24. exp Nicotine/ or exp Tobacco Smoking/ or exp Smoking Cessation/ or cigarette.mp. or exp Drug Dependency/
25. exp Alcohol Drinking Patterns/ or exp Drinking Behavior/ or exp Alcohol Drinking Attitudes/ or exp Binge Drinking/ or drinking.mp.
26. exp Physical Activity/ or exp Intervention/ or exp Exercise/ or exp Physical Fitness/ or exp Motor Performance/ or physical training.mp.
27. 23 and 24
28. 23 and 25

For peer review only

29. 23 and 26
30. 24 and 25
31. 24 and 26
32. 25 and 26
33. ((life-style or life style or lifestyle or healthcare or health care) adj3 (intervention$ or educat$ or advice or alter$ or change$)).mp.
34. primary prevention.mp.
35. (risk factor$ adj3 (reduc$ or manage$ or managing or intervent$ or program$)).sh.
36. (educat$ adj3 (program$ or patient$)).mp.
37. (non pharmacologic$ or nonpharmacologic$).mp.
38. (risk factor$ adj3 modif$).mp.
39. ((lifestyle or life-style or life style) adj3 modif$).mp.
40. (behavi?r$ adj3 (intervention$ or program$ or modif$ or change$ or alter$)).mp.
41. (promot$ adj3 (health or healthcare or health care)).mp.
42. 15 or 16 or 17 or 18 or 19 or 20 or 21 or 22 or 27 or 28 or 29 or 30 or 31 or 32 or 33 or 34 or 35 or 36 or 37 or 38 or 39 or 40 or 41
43. 14 and 42
44. random$.ti,ab.
45. factorial$.ti,ab.
46. (crossover$ or cross over$ or cross-over$).ti,ab.
47. placebo$.ti,ab.
48. (double$ adj blind$).ti,ab.
49. (singl$ adj blind$).ti,ab.
50. assign$.ti,ab.
51. allocat$.ti,ab.
52. volunteer$.ti,ab.
53. ("double-blind" or "random* assigned" or control).mp.
54. treatment effectiveness evaluation.mp.
55. treatment outcome clinical trial$.mp.
56. (controlled trial$ and clinical trial$).mp. [mp=title, abstract, heading word, table of contents, key concepts, original title, tests & measures]
57. 44 or 45 or 46 or 47 or 48 or 49 or 50 or 51 or 52 or 53 or 54 or 55 or 56
58. 43 and 57

For peer review only

Appendix B

Forest plots of pooled effect of multiple behaviour interventions on intervention outcomes.

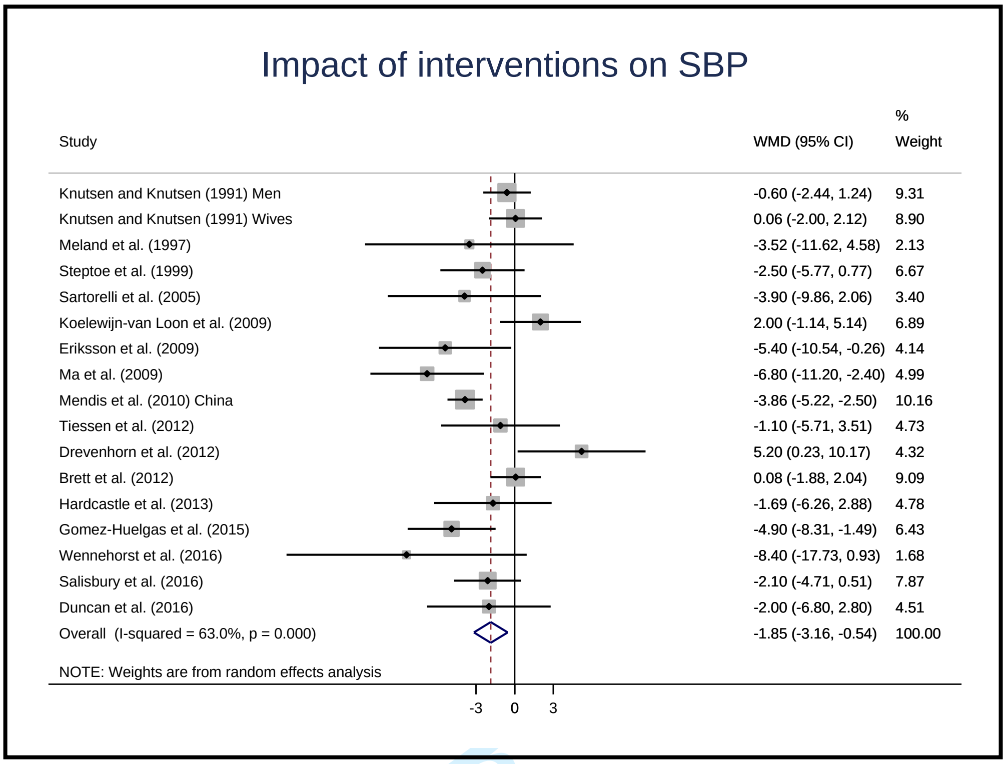

Pooled effect of multiple behaviour interventions on systolic blood pressure (mmHg). Random effects model used. MWD= mean weighted difference. 95% CI = 95% confidence intervals.

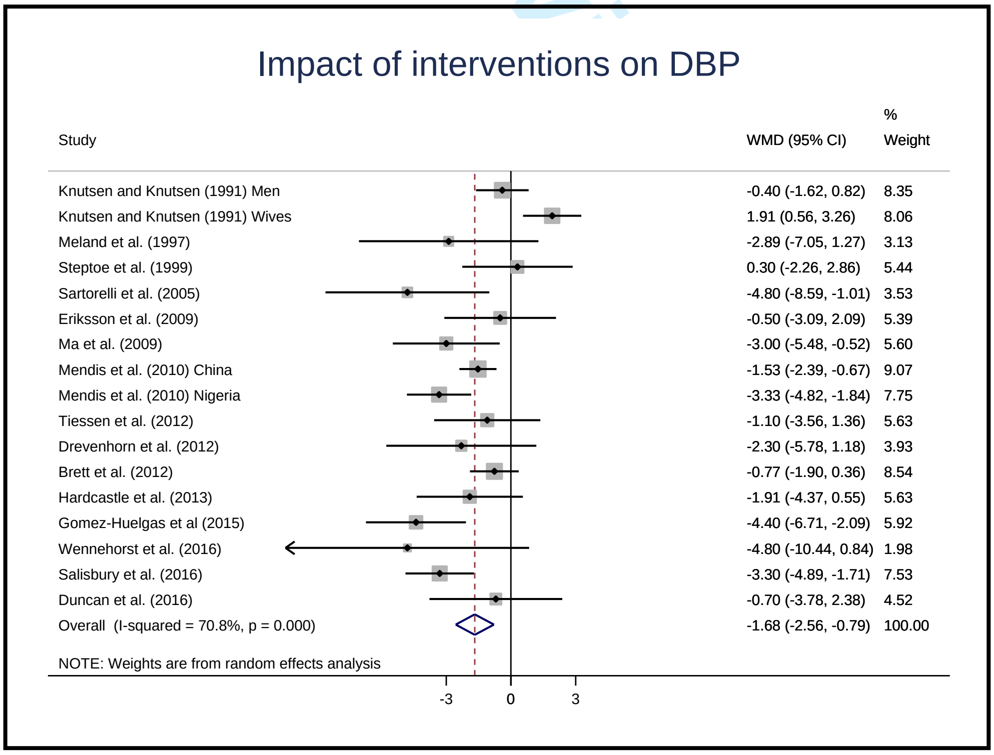

Pooled effect of multiple behaviour interventions on diastolic blood pressure (mmHg). Random effects model used. MWD= mean weighted difference. 95% CI = 95% confidence intervals.

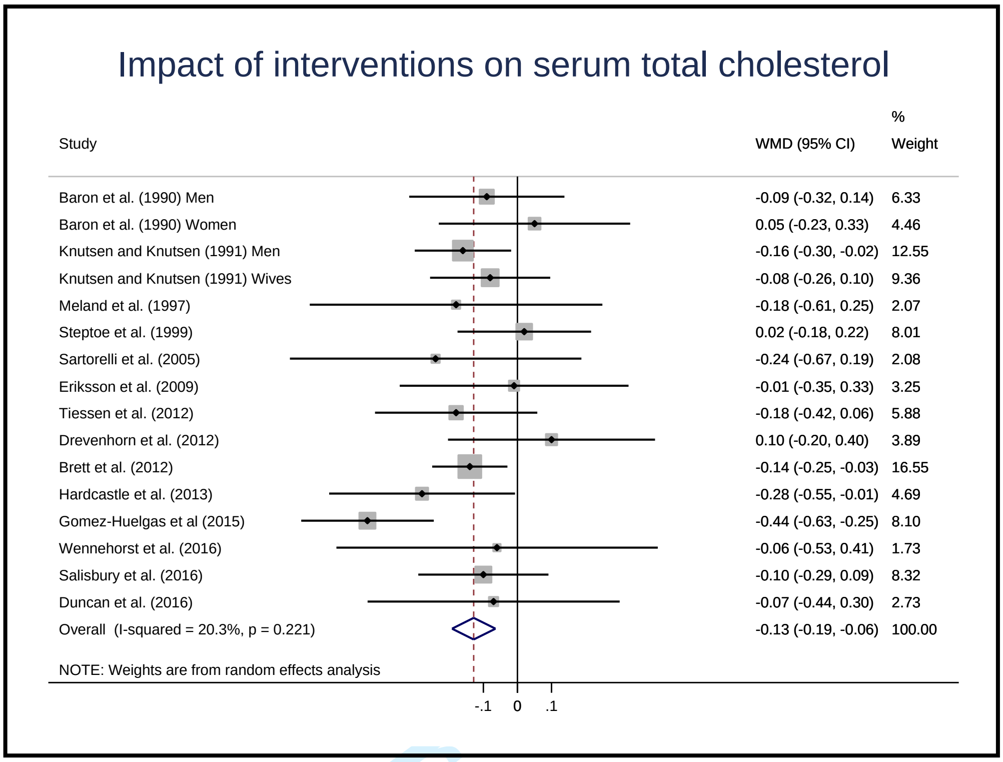

Pooled effect of multiple behaviour interventions on serum total cholesterol (mmol/L). Random effects model used. MWD= mean weighted difference. 95% CI = 95% confidence intervals.

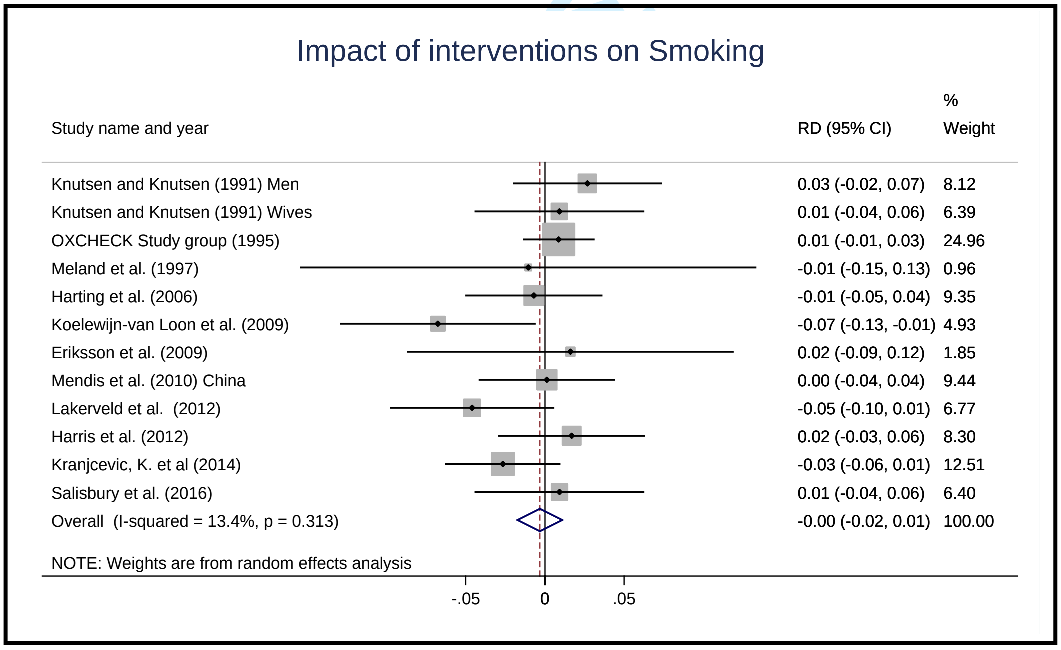

Pooled effect of multiple behaviour interventions on smoking prevalence. Random effects models used. RD= risk difference. 95% CI = 95% confidence intervals.

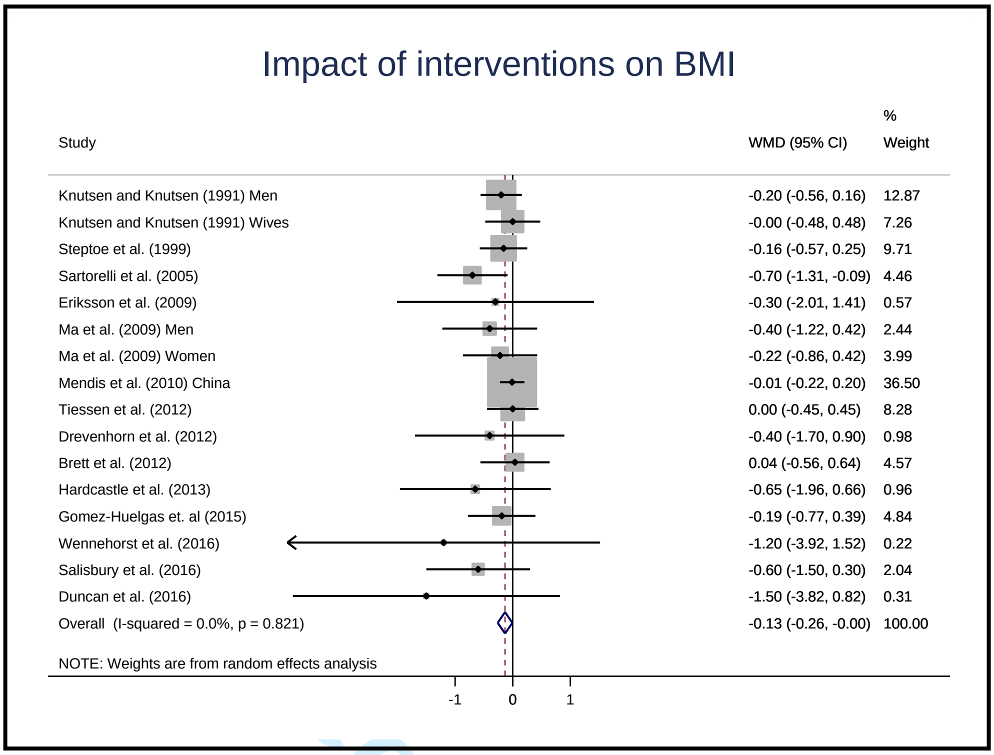

Pooled effect of multiple behaviour interventions on body mass index (Kg/m^2). Random effects model used. MWD= mean weighted difference. 95% CI = 95% confidence intervals.

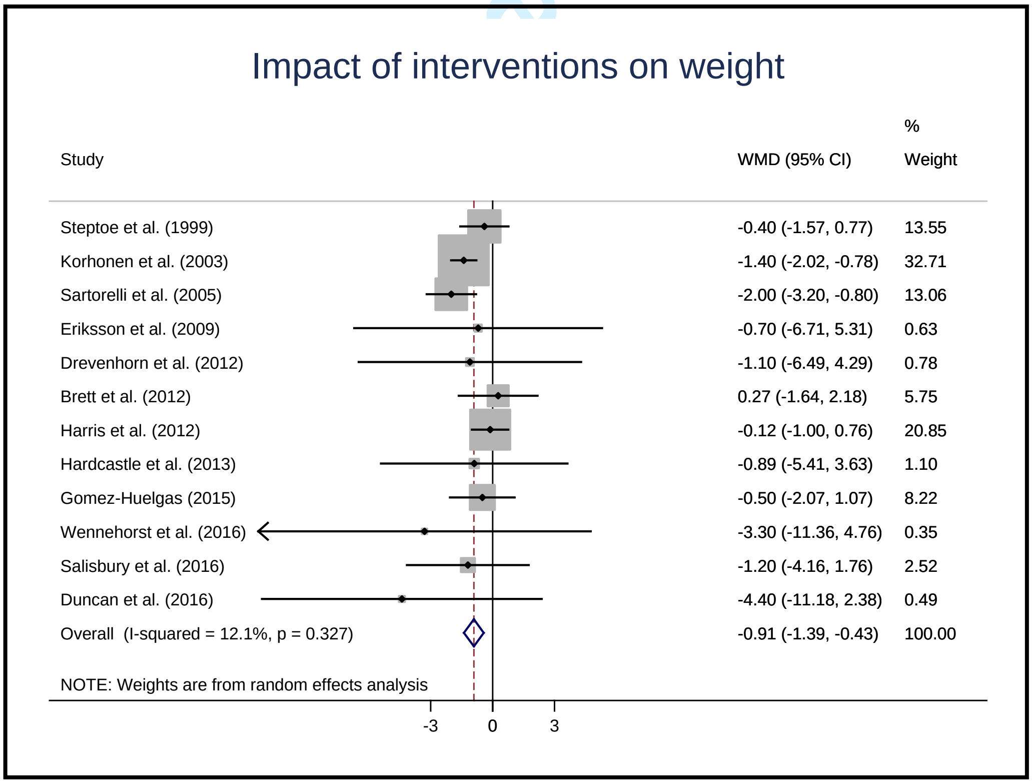

Pooled effect of multiple behaviour interventions on weight (Kg). Random effects model used. MWD= mean weighted difference. 95% CI = 95% confidence intervals.

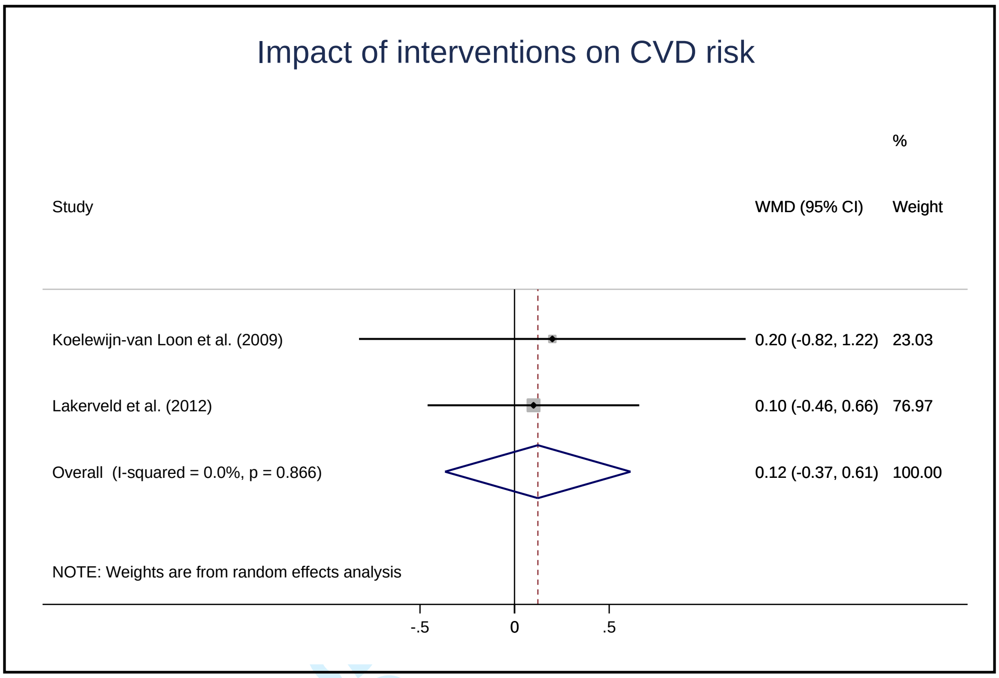

Pooled effect of multiple behaviour interventions on cardiovascular risk (SCORE). Random effect models used. MWD= mean weighted difference. 95% CI = 95% confidence intervals.

For peer review only

Supplementary table 1: Trial characteristics of included studies.

Study (Year)	Country	Number of Participants	Selection criteria	Targeted behaviours	Follow-up duration	Intervention reported outcomes
**Kranjčević, et al.** [1]	Croatia	1957	Men and women, aged ≥40.	Diet and PA.	18 months	CVD-risk, weight, BP, cholesterol, smoking, alcohol and PA.
**Vetter, et al.** [2]	United States	390	Men and women, aged≥21 years, BMI= 30-50kg/m^2, elevated waist circumference.	Diet and PA.	2 years	Weight, BP and cholesterol.
**Lakerveld, et al.** [3]	Netherlands	622	Men and women, aged: 30-50 years.	Diet, PA and smoking.	12 months	CVD-risk, smoking, diet and PA.
**Hardcastle, et al.** [4]	United Kingdom	334	Men and women, aged 18-65 years and have at least one CVD risk factor.	Diet and PA.	18 months	Weight, BP, cholesterol, diet and PA.
**Tiessen, et al.** [5]	Netherlands	201	Men aged: 50-75 years old and women aged: 55-75 years and CVD-risk (SCORE) ≥ 5%.	PA, diet and smoking.	12 months	CVD-risk, weight, BP, cholesterol, smoking and PA.
**Parra-Medina, et al.** [6]	United States	266	African-American women, aged≥35 years, baseline BP <160/95.	PA and diet.	12 months	Diet and PA.
**Drevenhorn, et al.** [7]	Sweden	153	Hypertensive patients, men and women aged <75 years, elevated BP, BMI ≥ 25, serum cholesterol ≥ 6.5 and/or serum triglycerides ≥ 2.3 and not reporting regular PA.	Smoking, alcohol, weight, PA and stress	2 years	Weight, BP, cholesterol, alcohol and PA.
**Brett, et al.** [8]	Australia	1200	Men and women aged 40-80 years, without a history of CVD.	Diet, PA and smoking.	12 months	CVD-risk, weight, BP and cholesterol.
**Harris, et al.** [9]	Australia	814	Men and women, aged 40-55 years with recorded diagnosis of hypertension and/or hyperlipidaemia or aged 56-64 years.	Diet, PA, smoking and alcohol.	12 months	CVD-risk, weight, BP, cholesterol, smoking, alcohol, diet and PA.
**Mendis, et al.** [10]	China	1209	Men and women aged 30-70 years with SBP in the range (140-179 mmHg).	Smoking cessation, PA and diet.	12 months	Weight, BP, smoking and diet.
	Nigeria	1188				
**Koelewijn-van Loon, et al.** [11]	Netherlands	615	One or more of the following: BP≥ 140 or on treatment for high BP; total cholesterol ≥ 6.5 or on treatment for high cholesterol; smoker aged ≥ 50 years (men) or ≥ 55 years (women); diabetes; a family history of CVD; and obese.	Smoking status, diet, PA and alcohol use.	12 months	CVD-risk, BP, cholesterol, smoking, diet and PA.
**Eriksson, et al.** [12]	Sweden	151	Men and women aged 18–65 years with hypertension, dyslipidaemia, type 2 diabetes or obesity.	Diet and PA.	3 years	Weight, BP, cholesterol, smoking and PA.
**Phelan, et al.** [13]	United States	224	Men and women aged 18–65 years and BMI of 30–45 kg/m^2.	Diet and PA.	12 months	Weight, BP, cholesterol and diet.

For peer review only

**Harting, et al.** [14]	Netherlands	1300	Men and women who have a greater than 20% risk (Framingham) of incurring a CVD event within 10 years.	Diet, PA and smoking.	18 months	Smoking, diet and PA.
**Korhonen, et al.** [15]	Finland	715	Men and women aged 25–74 years, with systolic BP 140–179 and/or diastolic BP 90–109 and/or on treatment for hypertension.	Diet and alcohol (also PA and smoking).	24 months	Weight, BP, cholesterol, alcohol, diet and PA.
**Baron, et al.** [16]	United Kingdom	368	Men and women aged 25 – 60 years.	Diet mainly, but changes in PA, alcohol and smoking were also mentioned.	12 months	Cholesterol and diet.
**Knutsen and Knutsen** [17]	Norway	1373 men, 1143 wives	Men aged 20 – 54 years and women aged 20-49 years, with no known CHD at baseline.	Diet changes, PA and smoking cessation.	6 years	CVD-risk, weight, BP, cholesterol, smoking and PA.
**Nilsson, et al.** [18]	Sweden	86	Men and women, born during the period 1925 – 1952, treated hypertensives.	Diet, smoking, PA and alcohol.	12 months	Weight, BP, cholesterol, smoking and diet.
**Wood, et al.** [19]	United Kingdom	7460 men, 5012 women	Men aged 40-59 and their families.	Smoking, weight, diet, alcohol, and PA.	12 months	CVD-risk, weight, BP, cholesterol and smoking.
**OXCHECK Study Group** [20]	United Kingdom	5559	Men and women aged 35-64.	Diet, smoking and PA.	3 years	CVD-risk, weight, BP, cholesterol, alcohol, diet, PA and smoking.
**Lindholm, et al.** [21]	Sweden	681	Men and women aged 30-59 years, had a moderate hyperlipidaemia, and at least two CVD risk factors.	Diet, smoking and PA.	18 months	CVD-risk, weight, BP, cholesterol, PA and smoking.
**Meland, et al.** [22]	Norway	127	Men aged 30 to 59 years.	Diet, smoking and PA.	12 months	CVD-risk, BP, cholesterol, PA and smoking.
**Avram, et al.** [23]	Romania	253	Men and women under 80 years, without history of CVD but defined as high risk individuals.	Diet and PA.	18 months	Weight, alcohol, diet and PA.
**Steptoe, et al.** [24]	United Kingdom	883	Men and women aged 18 – 69, total cholesterol of 6.5-9; smoker, BMI of 25-35 and lack of regular PA.	Smoking, diet and PA.	12 months	Weight, BP, cholesterol, diet and PA.
**Sartorelli, et al.** [25]	Brazil	104	Men and women aged 30-65 years, body mass index of 24-35 kg/m^2, and non-diabetic.	Diet and PA.	12 months	Weight, BP, cholesterol, diet and PA.
**Ma, et al.** [26]	United States	419	Men and women aged 35 to 85 years, had moderately to severely elevated levels of major modifiable CVD risk factors.	PA, diet and stress reduction.	15 months	CVD-risk, weight, BP and cholesterol.
**Tibblin and Åberg** [27]	Sweden	400	Men and women aged 30 - 69 years, on hypertensive drugs	Diet, PA and stress management.	12 months	Weight, BP and cholesterol.

For peer review only

**Gomez-Huelgas et al (2015)[28]**	Spain	601	Men and women aged 18-80 years, with metabolic syndrome.	Diet and PA.	3 years	Weight, BP, cholesterol, diet and PA.
**Wennehorst et al. [29]**	Germany	83	Men and women aged 18-80 years who had either prediabetes, type 2 diabetes, or were at risk of developing diabetes and/or cardiovascular diseases.	Diet and PA.	12 months	Weight, BP, cholesterol.
**Salisbury et al. [30]**	United Kingdom	641	Men and women aged between 40 and 74 years, had a high risk of a cardiovascular event in the next 10 years, and had one or more of the following modifiable risk factors (systolic blood pressure ≥140 mm Hg, body mass index ≥30, being a current smoker, or any combination of these).	Smoking status, diet, PA and alcohol use.	12 months	CVD-risk, weight, BP, cholesterol, diet, PA and smoking.
**Duncan et al.[31]**	New Zealand	320	Adults aged 35 to 65 years, a 5-year CVD risk of at least 7%, and/or a BMI of at least 33 kg/m2 for participants younger than 50 years	Diet and PA.	12 months	CVD-risk, weight, BP, cholesterol, diet and PA.

Note: BMI: body mass index, PA: physical activity, BP: blood pressure, CVD: cardiovascular diseas

For peer review only

References:

1. Kranjčević K, Marković BB, Lalić DI, et al. Is a targeted and planned GP intervention effective in cardiovascular disease prevention? A randomized controlled trial. Medical science monitor: international medical journal of experimental and clinical research 2014;**20**:1180.
2. Vetter ML, Wadden TA, Chittams J, et al. Effect of lifestyle intervention on cardiometabolic risk factors: results of the POWER-UP trial. International Journal of Obesity 2013;**37**:S19-S24.
3. Lakerveld J, Bot SD, Chinapaw MJ, et al. Motivational interviewing and problem solving treatment to reduce type 2 diabetes and cardiovascular disease risk in real life: a randomized controlled trial. Int J Behav Nutr Phys Act 2013;**10**(47):10.1186.
4. Hardcastle SJ, Taylor AH, Bailey MP, et al. Effectiveness of a motivational interviewing intervention on weight loss, physical activity and cardiovascular disease risk factors: a randomised controlled trial with a 12-month post-intervention follow-up. Int J Behav Nutr Phys Act 2013;**10**(40):1-16.
5. Tiessen AH, Smit AJ, Broer J, et al. Randomized controlled trial on cardiovascular risk management by practice nurses supported by self-monitoring in primary care. BMC family practice 2012;**13**(1):1.
6. Parra-Medina D, Wilcox S, Salinas J, et al. Results of the Heart Healthy and Ethnically Relevant Lifestyle trial: a cardiovascular risk reduction intervention for African American women attending community health centers. American journal of public health 2011;**101**(10):1914-21.
7. Drevenhorn E, Bengtson A, Nilsson PM, et al. Consultation training of nurses for cardiovascular prevention–a randomized study of 2 years duration. Blood pressure 2012;**21**(5):293-99.
8. Brett T, Arnold-Reed D, Phan C, et al. The Fremantle Primary Prevention Study: a multicentre randomised trial of absolute cardiovascular risk reduction. Br J Gen Pract 2012;**62**(594):e22-e28.
9. Harris MF, Fanaian M, Jayasinghe UW, et al. A cluster randomised controlled trial of vascular risk factor management in general practice. Med J Aust 2012;**197**(7):387-93.
10. Mendis S, Johnston SC, Fan W, et al. Cardiovascular risk management and its impact on hypertension control in primary care in low-resource settings: a cluster-randomized trial. Bulletin of the World Health Organization 2010;**88**(6):412-19.
11. Koelewijn-van Loon MS, van der Weijden T, van Steenkiste B, et al. Involving patients in cardiovascular risk management with nurse-led clinics: a cluster randomized controlled trial. Canadian Medical Association Journal 2009;**181**(12):E267-E74.
12. Eriksson MK, Franks PW, Eliasson M. A 3-year randomized trial of lifestyle intervention for cardiovascular risk reduction in the primary care setting: the Swedish Björknäs study. PloS one 2009;**4**(4):e5195.
13. Phelan S, Wadden T, Berkowitz R, et al. Impact of weight loss on the metabolic syndrome. International journal of obesity 2007;**31**(9):1442-48.
14. Harting J, van Assema P, van Limpt P, et al. Cardiovascular prevention in the Hartslag Limburg project: effects of a high-risk approach on behavioral risk factors in a general practice population. Preventive medicine 2006;**43**(5):372-78.
15. Korhonen M, Kastarinen M, Uusitupa M, et al. The effect of intensified diet counseling on the diet of hypertensive subjects in primary health care: a 2-year open randomized controlled trial of lifestyle intervention against hypertension in eastern Finland. Preventive medicine 2003;**36**(1):8-16.

16. Baron JA, Gleason R, Crowe B, et al. Preliminary trial of the effect of general practice based nutritional advice. Br J Gen Pract 1990;**40**(333):137-41.
17. Knutsen SF, Knutsen R. The Tromsø Survey: the Family Intervention study—the effect of intervention on some coronary risk factors and dietary habits, a 6-year follow-up. Preventive medicine 1991;**20**(2):197-212.
18. Nilsson PM, Lindholm LH, Scherstén BF. Life style changes improve insulin resistance in hyperinsulinaemic subjects: a one-year intervention study of hypertensives and normotensives in Dalby. Journal of hypertension 1992;**10**(9):1071-78.
19. Wood D, Kinmonth A, Davies G, et al. Randomised controlled trial evaluating cardiovascular screening and intervention in general practice: principal results of British family heart study. Bmj 1994;**308**(6924):313-20.
20. OXCHECK. Effectiveness of health checks conducted by nurses in primary care: final results of the OXCHECK study. BMJ: British Medical Journal 1995:1099-104.
21. Lindholm LH, Ekbom T, Dash C, et al. The impact of health care advice given in primary care on cardiovascular risk. BMJ 1995;**310**(6987):1105-09.
22. Meland E, Lærum E, Ulvik RJ. Effectiveness of two preventive interventions for coronary heart disease in primary care. Scandinavian journal of primary health care 1997;**15**(1):57-63.
23. Avram C, Iurciuc M, Craciun L, et al. Dietary and physical activity counseling in high-risk asymptomatic patients with metabolic syndrome–A primary care intervention. Journal of Food, Agriculture & Environment 2011;**9**(3&4):16-19.
24. Steptoe A, Day S, Doherty S, et al. Behavioural counselling in general practice for the promotion of healthy behaviour among adults at increased risk of coronary heart disease: randomised trialCommentary: Treatment allocation by the method of minimisation. Bmj 1999;**319**(7215):943-48.
25. Sartorelli DS, Sciarra EC, Franco LJ, et al. Beneficial effects of short-term nutritional counselling at the primary health-care level among Brazilian adults. Public health nutrition 2005;**8**(07):820-25.
26. Ma J, Berra K, Haskell WL, et al. Case management to reduce risk of cardiovascular disease in a county health care system. Archives of internal medicine 2009;**169**(21):1988-95.
27. Tibblin G, Åberg H. NON-PHARMACOLOGICAL TREATMENT OF HYPERTENSION IN TWO STEPS-1 YEAR REPORT FROM EIGHT HEALTH CENTRES. Acta Medica Scandinavica 1986;**220**(S714):105-12.
28. Gomez-Huelgas R, Jansen-Chaparro S, Baca-Osorio AJ, et al. Effects of a long-term lifestyle intervention program with Mediterranean diet and exercise for the management of patients with metabolic syndrome in a primary care setting. European Journal of Internal Medicine 2015;**26**(5):317-23.
29. Wennehorst K, Mildenstein K, Saliger B, et al. A comprehensive lifestyle intervention to prevent type 2 diabetes and cardiovascular diseases: The german chip trial. Prevention Science 2016:No Pagination Specified.
30. Salisbury C, O'Cathain A, Thomas C, et al. Telehealth for patients at high risk of cardiovascular disease: Pragmatic randomised controlled trial. BMJ (Online) 2016;**353 (no pagination)**(i2647).
31. Duncan S, Goodyear-Smith F, McPhee J, et al. Family-centered brief intervention for reducing obesity and cardiovascular disease risk: A randomized controlled trial. Obesity 2016;**24**(11):2311-18.

For peer review only

Supplementary table 2: Intervention components and behaviour change techniques employed.

Study (Year)	Study groups	Who delivered it	BCTs [1]	Mode of delivery	No. of sessions	Duration of sessions (in mins)
**Kranjčević, et al. [1]**	Intervention	GPs	1.3, 2.1, 9.1	Face to face and written materials	5	Unclear
	Control		Unclear	Unclear	Unclear	Unclear
**Vetter, et al. [2]**	Intervention 1	PCP and lifestyle coach.	1.1, 1.5, 2.3, 8.7, 9.1	Face to face and written materials	32	Visits: 5-7 mins, counselling: 10-15 mins.
	Intervention 2		1.1, 1.5, 2.3, 8.7, 9.1, 11.1	Face to face and written materials	32	Visits: 5-7 mins, counselling: 10-15 mins.
	Control		1.7	Face to face	8	Visits: 5-7 mins.
**Lakerveld, et al. [3]**	Intervention	Nurse	1.2, 1.6	Face to face and phone sessions.	9	Face to face sessions: 30 mins.
	Control		4.1, 5.1	Written materials.	Unclear	Unclear
**Hardcastle, et al. [4]**	Intervention	PA specialist and dietician	1.1, 1.5, 9.2	Face to face.	5	20-30 mins.
	Control		5.1	Written materials.	Unclear	Unclear
**Tiessen, et al. [5]**	Intervention	Practice nurses.	2.2, 2.3, 2.4, 5.1	Face to face.	7	First session: 20 min, other sessions based on patient preference.
	Control		5.1	Face to face and written materials.	One	Unclear.
**Parra-Medina, et al. [6]**	Intervention	PCP, health educators and nurses	1.1, 1.2.	Face to face and telephone sessions and written materials.	Up to 15	First session: 60 mins. Following sessions: 20 mins.
	Control		1.1	Face to face and written materials.	One	5-10 mins.
**Drevenhorn, et al. [7]**	Intervention	Nurses	1.1, 1.5, 5.3, 9.2, 10.4, 11.2	Face to face	Unclear	Unclear
	Control		Unclear	Unclear	Unclear	Unclear
**Brett, et al. [8]**	Intervention		1.1, 1.3, 2.7	Face to face	5	Unclear
	Control	GPs	1.1, 1.3, 2.7	Face to face	2	Unclear
**Harris, et al. [9]**	Intervention	Health practitioner, dietitian or PT	1.1, 1.2, 2.3, 4.1, 6.1, 9.1	Face to face	6	90 mins/ session.
	Control		Unclear	Unclear	Unclear	Unclear
**Mendis, et al. [10]**	Intervention	Health-care workers	2.6, 4.1	Face to face and written materials	4	Unclear
	Control		Unclear	Unclear	Unclear	Unclear
**Koelewijn-van Loon, et al. [11]**	Intervention	Nurses	1.1, 1.2, 1.4, 1.5, 5.1, 9.2	Face to face and telephone sessions	3	Face to face: 10-20 mins, telephone: 10 mins.
	Control		5.1	Face to face	One	Unclear
**Eriksson, et al. [12]**	Intervention	Dietician, PT and assistants.	1.1, 1.2, 1.3, 1.4, 4.1, 5.1, 8.1, 8.7, 9.1, 9.2	Face to face	56	Unclear.
	Control		9.1	Face to face and written materials.	One	Unclear

For peer review only

**Phelan, et al.** [13]	Intervention 1	PCP	2.3, 11.1.	Face to face and written materials.	8	5-10 mins.
	Intervention 2	Psychologist	1.5, 2.3, 9.1	Group sessions.	29	90 mins
	Intervention 3	Psychologist, PCP	1.5, 2.3, 9.1, 11.1	Face to face, group sessions and written material.	37	Face to face: 5-10 mins, group sessions: 90 mins.
	Intervention 4	PCP	1.5, 2.3, 9.1, 11.1	Face to face and written materials.	8	5-10 mins.
**Harting, et al.** [14]	Intervention	Practice assistant and dietician.	1.1, 1.4, 9.1, 11.1	Face to face, telephone sessions and written materials.	Unclear	Unclear
	Control		Unclear	Unclear	Unclear	Unclear
**Korhonen, et al.** [15]	Intervention	Healthcare centre personnel.	1.1, 1.3, 1.4, 2.3, 2.5,4.1, 9.1	Face to face.	7	Unclear
	Control		Unclear	Unclear	Unclear	Unclear
**Baron, et al.** [16]	Intervention	Nurse	5.1, 9.1	Face to face, group sessions and written material.	Unclear	30 mins.
	Control		Unclear	Unclear	Unclear	Unclear
**Knutsen and Knutsen** [17]	Intervention	Physicians and dieticians	1.1, 4.1, 5.1, 6.1, 9.1	Face to face and telephone sessions.	8	Unclear
	Control		Unclear	Unclear	Unclear	Unclear
**Nilsson, et al.** [18]	Intervention	Nurse, dietician or PT.	1.1, 2.2, 3.1, 4.1, 6.1, 9.1, 12.5	Face to face, group sessions and videotapes.	Unclear	Unclear
	Control		2.2, 5.1	Face to face	One	Unclear
**Wood, et al.** [19]	Intervention	Nurses	1.1, 2.7, 5.1, 6.2, 9.1	Face to face and written materials	Unclear	First session: 90 mins.
	Control		9.1	Face to face	One	45 mins
**OXCHECK Study Group** [20]	Intervention	Nurses	1.3, 2.7, 9.1,	Face to face	Unclear	Initial session: 45-60 mins, following sessions:10-20 mins.
	Control		Unclear	None	None	None
**Lindholm, et al.** [21]	Intervention	Doctors and nurses	2.3, 4.1, 5.1, 6.2, 9.1	Face to face, group sessions and written materials	11	Five group sessions: 90 mins, one group session: all day.
	Control		9.1	Face to face and written materials	5	Unclear
**Meland, et al.** [22]	Intervention	GPs	1.8, 2.3, 8.7, 9.1, 11.2	Face to face and written materials	4	Unclear
	Control		9.1	Face to face and written materials	4	Unclear
**Avram, et al.** [23]	Intervention	GPs	1.1, 9.1	Face to face and telephone sessions	21	Face to face sessions:30 mins.
	Control		Unclear	Written materials	None	None
**Steptoe, et al.** [24]	Intervention	Nurses	1.1, 1.4, 9.1, 11.1	Face to face and telephone sessions	2-3	Face to face sessions:20 mins.
	Control		Unclear	Unclear	Unclear	Unclear
**Sartorelli, et al.** [25]	Intervention	Nutritionist	1.1, 1.4, 9.1	Face to face and group sessions and written materials.	4	Unclear
	Control		Unclear	Group session and written materials	1	Unclear

For peer review only

**Ma, et al.** [26]	Intervention	Nurses and dietitians	1.1, 1.2, 1.7, 9.1, 11.1, 11.2	Face to face	8-10	30-60 mins
	Control		Unclear	Unclear	Unclear	Unclear
**Tibblin and Åberg** [27]	Intervention	Nurses and physicians	2.5, 6.1, 9.1	Face to face, group sessions and videotapes and audiotapes.	15	Unclear
	Control		2.5, 9.1	Face to face	15	Unclear
**Gomez-Huelgas et al.** [28]	Intervention	Nurses and physicians	1.3, 1.4, 2.5, 4.1, 9.1	Face to face, group sessions and written materials.	27	Health assessment: 15 mins, nursing visits: 30 mins.
	Control		2.5, 9.1	Face to face and written materials	24	10 mins.
**Wennehorst et al.** [29]	Intervention	Physician and nutritionist	1.4, 3.1, 4.1, 9.1, 11.2.	Face to face, group sessions and written materials.	16	2.5 hrs/ session.
	Control		Unclear	Unclear	Unclear	Unclear
**Salisbury et al.** [30]	Intervention	Health advisors	1.1, 1.6, 2.4, 5.1, 9.1, 11.1.	Computerised behavioural management programme and telephone sessions.	12	Telephone sessions: an average of 18 mins/session.
	Control		Unclear	Unclear	Unclear	Unclear
**Duncan et al.** [31]	Intervention	Trained health promoter	1.1, 1.4, 1.5, 2.3, 2.6, 3.1, 8.7.	Face to face group sessions and written materials.	5	60mins/ session.
	Control		2.6	Face to face	unclear	Unclear

[1] as coded in Michie, Richardson et al.[32] taxonomy of behaviour change technique

Note: 1.1 Goal setting (behaviour); 1.2 Problem solving; 1.3 Goal setting (outcome); 1.4 Action planning; 1.5 Review behaviour goals(s); 1.6 Discrepancy between current behaviour and goal; 1.7 Review outcome goal(s); 1.8 Behavioural contract; 2.1 Monitoring of behaviour by others without feedback; 2.2 Feedback on behaviour; 2.3 self-monitoring of behaviour; 2.4 Self-monitoring of outcome(s) of behaviour; 2.5 Monitoring of outcomes of behaviour without feedback; 2.6 Biofeedback; 2.7 Feedback on outcome(s) of behaviour; 3.1 Social support (unspecified); 4.1 Instructions on how to perform a behaviour; 5.1 Information about health consequences; 5.3 Information about social and environmental consequences; 6.1 Demonstration of the behaviour; 6.2 Social comparison; 8.1 Behavioural practice/rehearsal; 8.7 Graded tasks; 9.1 Credible source; 9.2 Pros and cons; 10.4 Social reward; 11.1 Pharmacological support; 11.2 Reduce negative emotions; 12.5 Adding objects to the environment; PT Physiotherapist, PA Physical activity

For peer review only

References:

1. Kranjčević K, Marković BB, Lalić DI, et al. Is a targeted and planned GP intervention effective in cardiovascular disease prevention? A randomized controlled trial. Medical science monitor: international medical journal of experimental and clinical research 2014;**20**:1180.
2. Vetter ML, Wadden TA, Chittams J, et al. Effect of lifestyle intervention on cardiometabolic risk factors: results of the POWER-UP trial. International Journal of Obesity 2013;**37**:S19-S24.
3. Lakerveld J, Bot SD, Chinapaw MJ, et al. Motivational interviewing and problem solving treatment to reduce type 2 diabetes and cardiovascular disease risk in real life: a randomized controlled trial. Int J Behav Nutr Phys Act 2013;**10**(47):10.1186.
4. Hardcastle SJ, Taylor AH, Bailey MP, et al. Effectiveness of a motivational interviewing intervention on weight loss, physical activity and cardiovascular disease risk factors: a randomised controlled trial with a 12-month post-intervention follow-up. Int J Behav Nutr Phys Act 2013;**10**(40):1-16.
5. Tiessen AH, Smit AJ, Broer J, et al. Randomized controlled trial on cardiovascular risk management by practice nurses supported by self-monitoring in primary care. BMC family practice 2012;**13**(1):1.
6. Parra-Medina D, Wilcox S, Salinas J, et al. Results of the Heart Healthy and Ethnically Relevant Lifestyle trial: a cardiovascular risk reduction intervention for African American women attending community health centers. American journal of public health 2011;**101**(10):1914-21.
7. Drevenhorn E, Bengtson A, Nilsson PM, et al. Consultation training of nurses for cardiovascular prevention–a randomized study of 2 years duration. Blood pressure 2012;**21**(5):293-99.
8. Brett T, Arnold-Reed D, Phan C, et al. The Fremantle Primary Prevention Study: a multicentre randomised trial of absolute cardiovascular risk reduction. Br J Gen Pract 2012;**62**(594):e22-e28.
9. Harris MF, Fanaian M, Jayasinghe UW, et al. A cluster randomised controlled trial of vascular risk factor management in general practice. Med J Aust 2012;**197**(7):387-93.
10. Mendis S, Johnston SC, Fan W, et al. Cardiovascular risk management and its impact on hypertension control in primary care in low-resource settings: a cluster-randomized trial. Bulletin of the World Health Organization 2010;**88**(6):412-19.
11. Koelewijn-van Loon MS, van der Weijden T, van Steenkiste B, et al. Involving patients in cardiovascular risk management with nurse-led clinics: a cluster randomized controlled trial. Canadian Medical Association Journal 2009;**181**(12):E267-E74.
12. Eriksson MK, Franks PW, Eliasson M. A 3-year randomized trial of lifestyle intervention for cardiovascular risk reduction in the primary care setting: the Swedish Björknäs study. PloS one 2009;**4**(4):e5195.
13. Phelan S, Wadden T, Berkowitz R, et al. Impact of weight loss on the metabolic syndrome. International journal of obesity 2007;**31**(9):1442-48.
14. Harting J, van Assema P, van Limpt P, et al. Cardiovascular prevention in the Hartslag Limburg project: effects of a high-risk approach on behavioral risk factors in a general practice population. Preventive medicine 2006;**43**(5):372-78.
15. Korhonen M, Kastarinen M, Uusitupa M, et al. The effect of intensified diet counseling on the diet of hypertensive subjects in primary health care: a 2-year open randomized controlled trial of lifestyle intervention against hypertension in eastern Finland. Preventive medicine 2003;**36**(1):8-16.
16. Baron JA, Gleason R, Crowe B, et al. Preliminary trial of the effect of general practice based nutritional advice. Br J Gen Pract 1990;**40**(333):137-41.

For peer review only

17. Knutsen SF, Knutsen R. The Tromsø Survey: the Family Intervention study—the effect of intervention on some coronary risk factors and dietary habits, a 6-year follow-up. Preventive medicine 1991;**20**(2):197-212.
18. Nilsson PM, Lindholm LH, Scherstén BF. Life style changes improve insulin resistance in hyperinsulinaemic subjects: a one-year intervention study of hypertensives and normotensives in Dalby. Journal of hypertension 1992;**10**(9):1071-78.
19. Wood D, Kinmonth A, Davies G, et al. Randomised controlled trial evaluating cardiovascular screening and intervention in general practice: principal results of British family heart study. Bmj 1994;**308**(6924):313-20.
20. OXCHECK. Effectiveness of health checks conducted by nurses in primary care: final results of the OXCHECK study. BMJ: British Medical Journal 1995:1099-104.
21. Lindholm LH, Ekbom T, Dash C, et al. The impact of health care advice given in primary care on cardiovascular risk. BMJ 1995;**310**(6987):1105-09.
22. Meland E, Lærum E, Ulvik RJ. Effectiveness of two preventive interventions for coronary heart disease in primary care. Scandinavian journal of primary health care 1997;**15**(1):57-63.
23. Avram C, Iurciuc M, Craciun L, et al. Dietary and physical activity counseling in high-risk asymptomatic patients with metabolic syndrome–A primary care intervention. Journal of Food, Agriculture & Environment 2011;**9**(3&4):16-19.
24. Steptoe A, Day S, Doherty S, et al. Behavioural counselling in general practice for the promotion of healthy behaviour among adults at increased risk of coronary heart disease: randomised trialCommentary: Treatment allocation by the method of minimisation. Bmj 1999;**319**(7215):943-48.
25. Sartorelli DS, Sciarra EC, Franco LJ, et al. Beneficial effects of short-term nutritional counselling at the primary health-care level among Brazilian adults. Public health nutrition 2005;**8**(07):820-25.
26. Ma J, Berra K, Haskell WL, et al. Case management to reduce risk of cardiovascular disease in a county health care system. Archives of internal medicine 2009;**169**(21):1988-95.
27. Tibblin G, Åberg H. NON-PHARMACOLOGICAL TREATMENT OF HYPERTENSION IN TWO STEPS-1 YEAR REPORT FROM EIGHT HEALTH CENTRES. Acta Medica Scandinavica 1986;**220**(S714):105-12.
28. Gomez-Huelgas R, Jansen-Chaparro S, Baca-Osorio AJ, et al. Effects of a long-term lifestyle intervention program with Mediterranean diet and exercise for the management of patients with metabolic syndrome in a primary care setting. European Journal of Internal Medicine 2015;**26**(5):317-23.
29. Wennehorst K, Mildenstein K, Saliger B, et al. A comprehensive lifestyle intervention to prevent type 2 diabetes and cardiovascular diseases: The german chip trial. Prevention Science 2016:No Pagination Specified.
30. Salisbury C, O'Cathain A, Thomas C, et al. Telehealth for patients at high risk of cardiovascular disease: Pragmatic randomised controlled trial. BMJ (Online) 2016;**353 (no pagination)**(i2647).
31. Duncan S, Goodyear-Smith F, McPhee J, et al. Family-centered brief intervention for reducing obesity and cardiovascular disease risk: A randomized controlled trial. Obesity 2016;**24**(11):2311-18.
32. Michie S, Richardson M, Johnston M, et al. The behavior change technique taxonomy (v1) of 93 hierarchically clustered techniques: building an international consensus for the reporting of behavior change interventions. Annals of behavioral medicine 2013;**46**(1):81-95.

For peer review only

Supplementary table 3: Risk of bias assessment.

Study (Year)	Risk of bias					
	Sequence generation (randomisation methods) [a]	Allocation concealment [b]	Blinding of participants and personnel to study group allocation [c]	Blinding of outcome assessors [d]	Incomplete outcome data [e]	Selective reporting [f]
Kranjčević, K. et al (2014)	Unclear	Unclear	Unclear	Unclear	High	Low
Vetter et al. (2013)	Low	Low	High	Low	High	Low
Lakerveld et al. (2012)	Low	Low	High	High	High	Low
Hardcastle et al. (2013)	Low	Low	Low	Low	High	Low
Tiessen et al. (2012)	Low	Low	High	High	Unclear	Low
Parra-Medina et al. (2011)	Unclear	Unclear	Low	Low	High	High
Drevenhorn et al. (2012)	Unclear	Unclear	Unclear	Unclear	High	Low
Brett et al. (2012)	Low	High	High	High	Low	High
Harris et al. (2012)	Low	Low	High	Low	Low	Low
Mendis et al. (2010)	Unclear	Unclear	High	High	Low	Unclear
Koelewijn-van Loon et al. (2009)	Low	Low	High	High	High	Low
Eriksson et al. (2009)	Low	Low	High	High	Unclear	Low
Phelan et al. (2007)	Unclear	Unclear	Unclear	Unclear	Unclear	Unclear
Harting et al. (2006)	Unclear	Unclear	Unclear	Unclear	Unclear	Unclear
Korhonen et al. (2003)	High	High	High	Unclear	High	Low
Baron et al. (1990)	Unclear	Unclear	Unclear	Unclear	Unclear	Unclear
Knutsen and Knutsen (1991)	Low	Low	Unclear	Low	Low	Low
Nilsson et al. (1992)	Unclear	Unclear	Unclear	Low	Low	Low
Wood et al. (1994)	Unclear	Unclear	Unclear	Unclear	Unclear	Low
OXCHECK Study group (1995)	Unclear	Unclear	High	Low	Unclear	Low
Lindholm et al. (1995)	Unclear	Unclear	High	Unclear	Low	Low
Meland et al. (1997)	Unclear	Unclear	Unclear	Unclear	Unclear	Unclear
Avram et al. (2011)	Unclear	Unclear	Low	Low	Low	Unclear

Steptoe et al. (1999)	Low	Low	High	High	High	Low
Sartorelli et al. (2005)	Low	Low	Low	High	High	Unclear
Ma et al. (2009)	Low	Low	Low	Low	Unclear	Low
Åberg and Tibblin (1989)	Low	Low	Unclear	Unclear	Low	Unclear
Gomez-Huelgas et al. (2015)	Unclear	Unclear	High	High	High	Unclear
Wennehorst et al. (2016)	Low	Low	Unclear	Unclear	High	Unclear
Salisbury et al. (2016)	Low	Low	High	Low	Low	Low
Duncan et al. (2016)	Low	Low	High	Low	High	Low

[a] Assessment of whether or not methods used to generate the allocation sequence should produce comparable groups.
[b] Assessment of whether or not the method used to conceal allocation sequence is sufficient or not.
[c] Assessment of the methods used to blind study participants and personnel from knowing intervention allocation.
[d] Assessment of the methods used to blind study outcome assessors from knowing intervention allocation, and whether or not this method of blinding is sufficient.
[e] Assessment of whether incomplete outcome data were adequately dealt with. Studies missing outcome data for >20% of participants who underwent randomization were considered at high risk of bias, while studies missing <10% of participants who underwent randomization were considered at low risk of bias.
[f] Assessment of whether all outcome measures described in the introduction and methods section of the paper (and published protocols) were reported.

For peer review only

Supplementary table 4: Theory use evaluation using Theory Coding Scheme.

Study	Theory used	Item 1	Item 2	Item 3	Item 4	Item 5	Item 6	Item 7	Item 8	Item 9	Item 10	Item 11	Item 12	Item 13	Item 14	Item 15	Item 16	Item 17	Item 18	Item 19
Kranjčević, K. et al (2014)	Theory use is not reported	N/A	N/A	N/A	N/A	N/A	N/A	N/A	N/A	N/A	N/A	N/A	N/A	N/A	N/A	N/A	N/A	N/A	N/A	N/A
Vetter et al. (2013)	Social cognitive and behavioural self-management theory	Yes	Yes	No	No	Yes	No	No	Yes	Yes	No	Yes	No	N/A	A, B,C and D	No	No	No	No	No
Lakerveld et al. (2012)	Theory of planned behaviour and theory of self-regulation.	Yes	Yes	No	No	Yes	No	Yes	No	Yes	No	Yes	B	A	A and B	Yes	No	Yes	No	No
Hardcastle et al. (2013)	Theory use is not reported	N/A	N/A	N/A	N/A	N/A	N/A	N/A	N/A	N/A	N/A	N/A	N/A	N/A	N/A	N/A	N/A	N/A	N/A	N/A
Tiessen et al. (2012)	Stages of change	Yes	No	Yes	Yes	Do not know	No	No	No	No	No	No	No	N/A	A, B,C and D	Do not now	No	No	Do not know	No
Parra-Medina et al. (2011)	Trans-theoretical model and social cognitive theory	Yes	Yes	No	No	Yes	Yes	Yes	No	Yes	No	Yes	No	No	A	Do not know	No	No	Do not know	No
Drevenhorn et al. (2012)	Stages of changes model	Yes	Yes	Yes	No	Yes	Yes	Yes	No	Yes	Yes	No	No	N/A	A	Do not know	No	No	Do not know	No
Brett et al. (2012)	Theory use is not reported	N/A	N/A	N/A	N/A	N/A	N/A	N/A	N/A	N/A	N/A	N/A	N/A	N/A	N/A	N/A	N/A	N/A	N/A	N/A

For peer review only - http://bmjopen.bmj.com/site/about/guidelines.xhtml

Harris et al. (2012)	Stages of changes model	Yes	Yes	Yes	No	No	Yes	No	No	No	No	No	No	N/A	A and B	No	No	No	No	No
Mendis et al. (2010)	Theory use is not reported	N/A	N/A	N/A	N/A	N/A	N/A	N/A	N/A	N/A	N/A	N/A	N/A	N/A	N/A	N/A	N/A	N/A	N/A	N/A
Koelewijn-van Loon et al. (2009)	Theory use is not reported	N/A	N/A	N/A	N/A	N/A	N/A	N/A	N/A	N/A	N/A	N/A	N/A	N/A	N/A	N/A	N/A	N/A	N/A	N/A
Eriksson et al. (2009)	Stages of change model	Yes	Yes	Yes	No	No	No	No	No	No	No	No	No	N/A	No	No	No	No	No	No
Phelan et al. (2007)	Theory use is not reported	N/A	N/A	N/A	N/A	N/A	N/A	N/A	N/A	N/A	N/A	N/A	N/A	N/A	N/A	N/A	N/A	N/A	N/A	N/A
Harting et al. (2006)	Theory use is not reported	N/A	N/A	N/A	N/A	N/A	N/A	N/A	N/A	N/A	N/A	N/A	N/A	N/A	N/A	N/A	N/A	N/A	N/A	N/A
Korhonen et al. (2003)	Theory use is not reported	N/A	N/A	N/A	N/A	N/A	N/A	N/A	N/A	N/A	N/A	N/A	N/A	N/A	N/A	N/A	N/A	N/A	N/A	N/A
Baron et al. (1990)	Theory use is not reported	N/A	N/A	N/A	N/A	N/A	N/A	N/A	N/A	N/A	N/A	N/A	N/A	N/A	N/A	N/A	N/A	N/A	N/A	N/A
Knutsen and Knutsen (1991)	Theory use is not reported	N/A	N/A	N/A	N/A	N/A	N/A	N/A	N/A	N/A	N/A	N/A	N/A	N/A	N/A	N/A	N/A	N/A	N/A	N/A
Nilsson et al. (1992)	Theory use is not reported	N/A	N/A	N/A	N/A	N/A	N/A	N/A	N/A	N/A	N/A	N/A	N/A	N/A	N/A	N/A	N/A	N/A	N/A	N/A
Wood et al. (1994)	Theory use is not reported	N/A	N/A	N/A	N/A	N/A	N/A	N/A	N/A	N/A	N/A	N/A	N/A	N/A	N/A	N/A	N/A	N/A	N/A	N/A
OXCHECK Study group (1995)	Theory use is not reported	N/A	N/A	N/A	N/A	N/A	N/A	N/A	N/A	N/A	N/A	N/A	N/A	N/A	N/A	N/A	N/A	N/A	N/A	N/A

For peer review only

For peer review only - http://bmjopen.bmj.com/site/about/guidelines.xhtml

Lindholm et al. (1995)	Theory use is not reported	N/A	N/A	N/A	N/A	N/A	N/A	N/A	N/A	N/A	N/A	N/A	N/A	N/A	N/A	N/A	N/A	N/A	N/A	N/A
Meland et al. (1997)	Theory use is not reported	N/A	N/A	N/A	N/A	N/A	N/A	N/A	N/A	N/A	N/A	N/A	N/A	N/A	N/A	N/A	N/A	N/A	N/A	N/A
Avram et al. (2011)	Theory use is not reported	N/A	N/A	N/A	N/A	N/A	N/A	N/A	N/A	N/A	N/A	N/A	N/A	N/A	N/A	N/A	N/A	N/A	N/A	N/A
Steptoe et al. (1999)	Stages of change model	Yes	Yes	Yes	No	No	No	No	No	No	No	No	Yes	C and F	N/A	N/A	N/A	N/A	N/A	N/A
Sartorelli et al. (2005)	Theory use is not reported	N/A	N/A	N/A	N/A	N/A	N/A	N/A	N/A	N/A	N/A	N/A	N/A	N/A	N/A	N/A	N/A	N/A	N/A	N/A
Ma et al. (2009)	Social cognitive theory and trans-theoretical model	Yes	No	No	No	No	No	No	No	No	No	No	No	N/A	N/A	N/A	N/A	No	No	No
Åberg and Tibblin (1989)	Theory use is not reported	N/A	N/A	N/A	N/A	N/A	N/A	N/A	N/A	N/A	N/A	N/A	N/A	N/A	N/A	N/A	N/A	N/A	N/A	N/A
Gomez-Huelgas et al. (2015)	Theory use is not reported	N/A	N/A	N/A	N/A	N/A	N/A	N/A	N/A	N/A	N/A	N/A	N/A	N/A	N/A	N/A	N/A	N/A	N/A	N/A
Wennehorst et al. (2016)	Theory use is not reported	N/A	N/A	N/A	N/A	N/A	N/A	N/A	N/A	N/A	N/A	N/A	N/A	N/A	N/A	N/A	N/A	N/A	N/A	N/A
Salisbury et al. (2016)	Theory use is not reported	N/A	N/A	N/A	N/A	N/A	N/A	N/A	N/A	N/A	N/A	N/A	N/A	N/A	N/A	N/A	N/A	N/A	N/A	N/A
Duncan et al. (2016)	Theory use is not reported	N/A	N/A	N/A	N/A	N/A	N/A	N/A	N/A	N/A	N/A	N/A	N/A	N/A	N/A	N/A	N/A	N/A	N/A	N/A

For peer review only

Item 1) Theory/ model of behaviour mentioned

Item 2) Targeted construct mentioned

Item 3) Intervention based on single theory

Item 4) Theory used to select recipients

Item 5) Theory used to select intervention techniques

Item 6) Theory used to tailor intervention techniques to recipients

Item 7) All intervention techniques are explicitly linked to theory construct

Item 8) At least one of the intervention techniques are explicitly linked to theory construct

Item 9) Group of techniques are linked to a group of constructs

Item 10) All theory relevant constructs are explicitly linked to at least one intervention technique.

Item 11) At least one of the theory relevant constructs are explicitly linked to at least one intervention technique.

Item 12) theory-relevant constructs are measured

Item 13) Quality of measures

Item 14) Randomization of participants' condition

Item 15) Changes in measured theory-relevant constructs

Item 16) Mediational analysis of constructs

Item 17) Results discussed in relation to theory

Item 18) Appropriate support for theory

Item 19) Results used to refine theory

For peer review only

PRISMA 2009 Checklist

Section/topic	#	Checklist item	Reported on page #
**TITLE**			
Title	1	Identify the report as a systematic review, meta-analysis, or both.	1
**ABSTRACT**			
Structured summary	2	Provide a structured summary including, as applicable: background; objectives; data sources; study eligibility criteria, participants, and interventions; study appraisal and synthesis methods; results; limitations; conclusions and implications of key findings; systematic review registration number.	2
**INTRODUCTION**			
Rationale	3	Describe the rationale for the review in the context of what is already known.	5 & 6
Objectives	4	Provide an explicit statement of questions being addressed with reference to participants, interventions, comparisons, outcomes, and study design (PICOS).	7
**METHODS**			
Protocol and registration	5	Indicate if a review protocol exists, if and where it can be accessed (e.g., Web address), and, if available, provide registration information including registration number.	N/A
Eligibility criteria	6	Specify study characteristics (e.g., PICOS, length of follow-up) and report characteristics (e.g., years considered, language, publication status) used as criteria for eligibility, giving rationale.	8 & 9
Information sources	7	Describe all information sources (e.g., databases with dates of coverage, contact with study authors to identify additional studies) in the search and date last searched.	9
Search	8	Present full electronic search strategy for at least one database, including any limits used, such that it could be repeated.	Appendix A
Study selection	9	State the process for selecting studies (i.e., screening, eligibility, included in systematic review, and, if applicable, included in the meta-analysis).	9 & 10
Data collection process	10	Describe method of data extraction from reports (e.g., piloted forms, independently, in duplicate) and any processes for obtaining and confirming data from investigators.	10
Data items	11	List and define all variables for which data were sought (e.g., PICOS, funding sources) and any assumptions and simplifications made.	10 & 11
Risk of bias in individual studies	12	Describe methods used for assessing risk of bias of individual studies (including specification of whether this was done at the study or outcome level), and how this information is to be used in any data synthesis.	10

For peer review only

Summary measures	13	State the principal summary measures (e.g., risk ratio, difference in means).	11 & 12
Synthesis of results	14	Describe the methods of handling data and combining results of studies, if done, including measures of consistency (e.g., I^2) for each meta-analysis.	11 & 12
Risk of bias across studies	15	Specify any assessment of risk of bias that may affect the cumulative evidence (e.g., publication bias, selective reporting within studies).	11 & 12
Additional analyses	16	Describe methods of additional analyses (e.g., sensitivity or subgroup analyses, meta-regression), if done, indicating which were pre-specified.	11 & 12
**RESULTS**			
Study selection	17	Give numbers of studies screened, assessed for eligibility, and included in the review, with reasons for exclusions at each stage, ideally with a flow diagram.	13 and figure 1
Study characteristics	18	For each study, present characteristics for which data were extracted (e.g., study size, PICOS, follow-up period) and provide the citations.	Supplementary table 1 & 2
Risk of bias within studies	19	Present data on risk of bias of each study and, if available, any outcome level assessment (see item 12).	Supplementary table 3
Results of individual studies	20	For all outcomes considered (benefits or harms), present, for each study: (a) simple summary data for each intervention group (b) effect estimates and confidence intervals, ideally with a forest plot.	Appendix B
Synthesis of results	21	Present results of each meta-analysis done, including confidence intervals and measures of consistency.	20
Risk of bias across studies	22	Present results of any assessment of risk of bias across studies (see Item 15).	21 - 26
Additional analysis	23	Give results of additional analyses, if done (e.g., sensitivity or subgroup analyses, meta-regression [see Item 16]).	21 - 26
**DISCUSSION**			
Summary of evidence	24	Summarize the main findings including the strength of evidence for each main outcome; consider their relevance to key groups (e.g., healthcare providers, users, and policy makers).	29
Limitations	25	Discuss limitations at study and outcome level (e.g., risk of bias), and at review-level (e.g., incomplete retrieval of identified research, reporting bias).	32
Conclusions	26	Provide a general interpretation of the results in the context of other evidence, and implications for future research.	33
**FUNDING**			
Funding	27	Describe sources of funding for the systematic review and other support (e.g., supply of data); role of funders for the systematic review.	33

For peer review only

From: Moher D, Liberati A, Tetzlaff J, Altman DG, The PRISMA Group (2009). Preferred Reporting Items for Systematic Reviews and Meta-Analyses: The PRISMA Statement. PLoS Med 6(7): e1000097. doi:10.1371/journal.pmed100009

For peer review only

For peer review only
